# Strengthening Structures in the Petiole–Lamina Junction of Peltate Leaves

**DOI:** 10.3390/biomimetics6020025

**Published:** 2021-04-02

**Authors:** Julian Wunnenberg, Annabell Rjosk, Christoph Neinhuis, Thea Lautenschläger

**Affiliations:** Department of Biology, Faculty of Science, Institute of Botany, Technische Universität Dresden, 01062 Dresden, Germany; julian.wunnenberg@tu-dresden.de (J.W.); christoph.neinhuis@tu-dresden.de (C.N.); thea.lautenschlaeger@tu-dresden.de (T.L.)

**Keywords:** peltate leaves, petiole, petiole–lamina junction, anatomy, strengthening structures

## Abstract

Peltate- or umbrella- shaped leaves are characterised by a petiole more or less centrally attached to the lamina on the abaxial side. The transition from the petiole to lamina in peltate leaves resembles a significant and abrupt geometrical change from a beam to a plate in a very compact shape. Since these leaves have not been subject of many studies, the distribution of that specific leaf morphology in the plant kingdom was investigated. Furthermore, the connection between the petiole and lamina of several peltate species was studied anatomically and morphologically, focusing on the reinforcing fibre strands. We found peltate leaves in 357 species representing 25 orders, 40 families and 99 genera. The majority are herbaceous perennials growing in shady, humid to wet habitats mainly distributed in the subtropical–tropical zones. Detailed anatomical investigation of 41 species revealed several distinct principles of how the transition zone between the petiole and lamina is organised. In-depth analysis of these different types accompanied by finite element-modelling could serve as inspiration for supporting structures in lightweight construction.

## 1. Introduction

Generally, leaves are important organs for plants, especially for photosynthesis and the production of organic compounds [1,2,3,4]. As such, the orientation of the leaves towards the sun is essential. Leaves are exposed to a multitude of mechanical stresses, caused by natural gravity and environmental factors such as wind and rain [1,5,6,7]. The petioles in particular must fulfil two important functions: (1) sustain their own weight and that of the lamina against gravity and (2) provide enough mechanical stability to withstand bending and twisting while being flexible enough not to be damaged [5,6,8,9]. Therefore, petioles can be described mechanically as elastic cantilevered beams [6,9,10].

To achieve the maximum rate of photosynthesis, the parameters of the leaf (shape, area, weight, petiole parameters, etc.) should be optimal [1]. However, there are many different leaf shapes in nature, each of which have different effects on the petioles [1,11]. The internal organisation of the petioles, including ground tissue and support or strengthening tissue and their respective proportions, are key parameters in mastering such mechanical requirements [1,9,11,12,13]. Among the tissues, parenchyma is the least differentiated with the lowest stiffness [1,9]. The highly variable collenchyma, also part of the ground tissue, is an important reinforcing structural element [1,12,14,15]. As both are viscoelastic tissues, stiffness and the contribution as strengthening tissues strongly depend on turgor pressure [1,9]. Besides the ground tissues, the petioles contain vascular tissue, formed by the phloem and xylem which serve as a pathway for water, nutrients and assimilates, both to and from the leaf blade [1,16]. The xylem also acts as a primary strengthening structure. Its stiffness is comparable to that of the sclerenchyma, which is the stiffest tissue in the petioles and often forms caps above the phloem or sclerenchymatic rings surrounding the vascular bundles [9,17].

**Figure 1:**
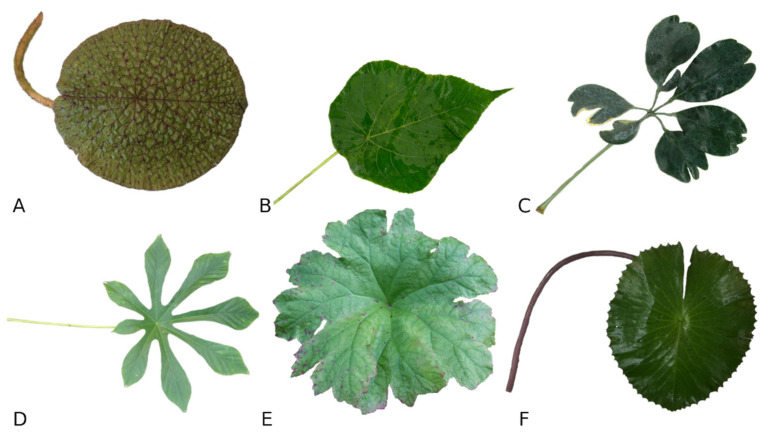
Variety of peltate leaf shapes (according to Troll, 1932 and Ebel, 1998 [18,20]). (**A**)—orbicular entire (*Euryale ferox*), (**B**)—ovate entire with pointed apex (*Macaranga tanarius*), (**C**)—palmately compound with leaflets (*Schefflera arboricola*), (**D**)—palmatipartite (*Cecropia peltata*), (**E**)—palmatifid (*Darmera peltata*), (**F**)—orbicular crenate toothed (*Nymphaea lotus*). (**A**,**E**)—central petiole insertion, (**B**–**D**,**F**)—eccentric insertion of petiole.

Most leaves consist of a petiole and the leaf blade, but the peltate leaf shape is not very common and has not been the subject of many studies [18]. In 1932, Wilhelm Troll provided one of the first explanations for the peltate leaf shape and defined them by their petiole insertion point [19]. The petioles are attached to the lamina on the abaxial side either in the centre or approximated to the leaf margin, distinguishing peltate leaves from the common marginal petiole insertion [19]. The unifacial petioles have a ring-shaped arrangement of the vascular bundles [19,20,21,22]. Although they are relatively small in number, the appearance of peltate leaves can be very diverse, including entire and centro-peltate leaves (e.g., *Nelumbo nucifera* or *Victoria cruziana*), as well as species with palmatifid (e.g., *Darmera peltata*), lobed (e.g., *Podophyllum peltatum*) and palmately compound leaves (e.g., *Schefflera arboricola*) (Figure 1) [18,20].

The reason for the evolution of peltate leaves is not yet fully understood. First theories suggested the facilitated orientation towards the sun and thus, the efficient light absorption [19,23]. Friedrich Ebel (1998) introduced a connection between peltation and certain chorological and habitat conditions [18]. In fact, herbaceous plants with peltate leaves are predominantly geophytes with rhizomes, stolons, bulbs, or tubers and almost all species grow in wet, humid or alternately wet locations preferentially close to lakes, ponds, swamps, meadows, streams, on river banks, in forests or amongst humid, shady to semi-shady rocks [18]. While genera with peltately leaved species are more or less evenly distributed in subtropical, meridional and temperate areas with ± oceanic climate (especially in eastern North and Central America and South East Asia), they are less common, relative to the total number of genera, in boreal zones, steppes, semi-deserts and desert habitats [18]. Only a few sources are available dealing with the distribution of species with peltate leaves. The first attempt to compile such a list was made by C. de Candolle in 1899 [24,25]. After several updates, Ebel (1989) was the last to provide a comprehensive list of peltate taxa distributed in 54 out of currently 416 angiosperm families (without distinguishing between the peltate leaf shape or variants and taxa with ascidiate leaves, e.g., Nepenthaceae, Cephalotaceae, Sarraceniaceae, or the Droseraceae) [18,26].

While some studies are available on the morphology and anatomy of peltate leaves and their petioles [19,20,21,22], the transition zone from the petiole to lamina has been analysed so far only in a single study by Sacher et al. (2019) [13]. Focusing on two peltate plant species (one from Araceae and one from Tropaeolaceae), the study identified two different variants of strengthening structures present in the compact transition zone [13]. These structures represent two types of branching of the vascular strands, which have been shown to act as a stabilising element. Such biological strengthening structures may have great potential as the inspiration for applications in lightweight construction [13].

This study intends to provide an overview about the occurrence and distribution of the peltate leaf shape among flowering plants and to analyse the morphology and anatomy of the petiole and the petiole–lamina transition zone of different peltate species in a wider approach focusing on characterising and categorising strengthening structures in the transition zone. With two types of strengthening structures found by Sacher et al. (2019) [13], our research aims to determine if there are more distinct principles of how the petiole–lamina junction in peltate leaves can be organised.

## 2. Materials and Methods

### 2.1. Screening

In search of plant species with peltate leaves, the living collection of the Botanical Garden of Technische Universität Dresden and the literature about the peltate leaf shape such as Uittien (1929), Troll (1932, 1939, 1955), Roth (1949, 1952) or Franck (1976) served as basis [19,20,21,22,25,27]. Internet sources as well as online libraries complemented the search for further species.

In this study, the term “peltate leaf” refers exclusively to the morphology of the visible foliage. It focused only on the vegetative, but not floral parts of the plant, such as stamens or carpels (Troll 1992) or peltate petals (Leinfellner 1958) [19,28].

The present work focused on species with an eccentric (margin approximated) or centrally attached petiole, with lobed, crenate or pinnate peltate leaves. All taxa have been compiled in a list down to species level together with information about their origin and distribution as well as their habit, habitat and petiole insertion.

The species for which fresh material was available were examined microscopically. Information about the habit, habitat and ecology of additional species were collected from different databases:

- eFloras, Missouri Botanical Garden and Harvard University Herbaria [29];
- POWO (Plants of the World Online), Royal Botanic Gardens, Kew [30];
- GBIF (Global Biodiversity Information Facility), GBIF Secretariat Copenhagen [31].

The nomenclature is based on information from Tropicos^®^ and © The Plant List (2013) [32,33]. The taxonomic classification follows the APG (Angiosperm Phylogeny Group) IV system of the Angiosperm phylogeny poster from 2019 [34]. The differentiation of peltation between centrally inserted and eccentrically inserted petioles is based on the definition in the “Manual of Leaf Architecture” by Ellis et al. (1999) [35]. The classification of the species distribution follows the list of the floral zones of the earth by Meusel et al. (1965–1992), edited and published by Jäger (2017) [36] and the floristic kingdoms by Pott (2014) [37].

### 2.2. Plant Material and Sampling

Plant samples were taken from the living collection of the Botanical Garden of the Technische Universität Dresden, Germany between September and December of 2019 and the Ecological–Botanical Garden of Universität Bayreuth, Germany in December 2019. All species were cultivated in the tropical greenhouses or under open-air conditions. Only non-damaged intact leaves and petioles were investigated (e.g., no frost or feeding damage) and a selection of small to large leaves. The leaf and the complete petiole were taken from the plant. To keep the samples as fresh as possible for direct analysis, they were transported to the laboratory in airtight containers and processed within a few hours. Samples of each species were preserved in 70% ethanol for later anatomical analysis.

### 2.3. Anatomy and Morphology

All fresh samples were scanned (Ricoh MP C3004ex, Ricoh Company, Ltd., Chuo, Tokyo, Japan) from both sides of the leaf using the program ImageJ (National Institutes of Health, Bethesda, MD, USA) to record the dimensions of leaves and petioles, including the length and width of the leaf and diameter of the petiole. In lobed or fully peltate leaves (almost circular), the midrib was used as orientation to determine the length of the leaf while the width (at the widest point) was measured perpendicular to the midrib. The leaf venation was separated from the intercostal areas of the lamina up to the second level of branching with a razor blade. The petioles were separated from the lamina and for both fresh and dry weight were determined using a precision scale (Mettler Toledo XA205DU, Mettler Toledo, Columbus, OH, USA). The fresh samples were dried for 5 days at 65 °C in a drying cabinet (Heraeus T12, Heraeus Instruments, Hanau, Germany) before measuring the dry weight.

Cross and longitudinal sections of leaves and petioles were prepared using a razor blade, stained with Astra-blue/Safranin (Morphisto GmbH, Frankfurt am Main, Germany) and observed with the reflected light microscope (Olympus SZX16, Olympus Corporation, Tokyo, Japan). Sections were photographed with a high-resolution microscope camera (Olympus DP6, Olympus Corporation, Tokyo, Japan). The number and area of the vascular bundles, the size of lignified areas and present types of tissue were measured for a small number of samples via images from the microscope camera using the image-processing program ImageJ (National Institutes of Health, Bethesda, MD, USA).

## 3. Results

### 3.1. Distribution of Peltate Species in the Plant Kingdom

The extent to which leaves can be described as peltate depends on the definition of the author. Therefore, the following list does not claim to be complete. Furthermore, several families or genera, with a high number of species with peltate leaves are not fully studied, e.g., *Begonia*, i.e., the list does not include all peltate leaved species of these genera or families.

The peltate leaf shape is found in all clades of angiosperms. In total, 357 species representing 99 genera, 40 families, and 25 orders were found (Table 1, for full list: see Supplementary Materials), including one order from the ANA grade (Nympheales) (ANA: Amborellales, Nymphaeales, Austrobaileyales), two from the magnoliids (Laurales and Piperales) and one order from the monocots (Alismatales). The remaining 21 orders belong to the eudicots. A fern genus with peltate leaved species could also be identified (Salviniales, *Marsilea*). Some families show a noticeably high number of peltate leaved species and were not studied completely, e.g., Piperaceae (two genera), Araceae (10 genera), Fabaceae (one genus) or Begoniaceae (one genus).

The analysis of additionally recorded parameters reveals that peltate species are preferentially distributed in the subtropical and tropical areas of the Neotropics and the Paleotropics. In South East Asia, the highest number of peltate leaved species are found in the paleotropic boreo-subtropical region, in Africa in the tropical zone, and in the Neotropics throughout the subtropical and tropical area. The southern austral zone contains considerably less peltate leaved genera than in the northern hemisphere, occurring mostly in special areas, such as the Capensis (*Oxalis*, *Ranunculus*, *Senecio*), New Zealand (*Ranunculus*) and the Juan Fernández Islands (*Gunnera*). *Tropaeolum*, *Gunnera* and *Ranunculus* are the only genera which are common in the Antarctic floristic kingdom. In the Holarctic, the main distribution areas are China (>15 genera) and meridional North America (6). The distribution of some genera also extends into the temperate and Boreal floral zone, while no representatives are recorded from the Arctic zone. The peltate leaved species of Europe are mainly found in the Mediterranean region in genera such as *Umbilicus*, *Hydrocotyle* or *Marsilea.*

### 3.2. Leaf Anatomy, Morphology and Characteristics of Selected Peltate Plant Species

**Figure 2:**
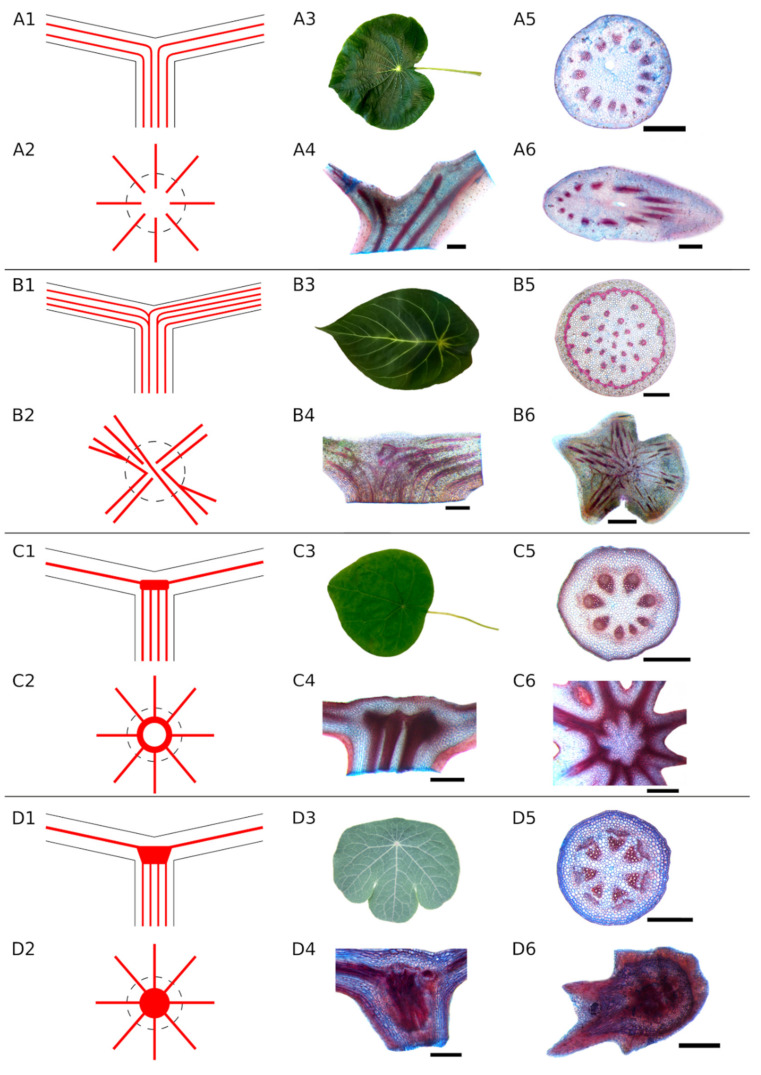

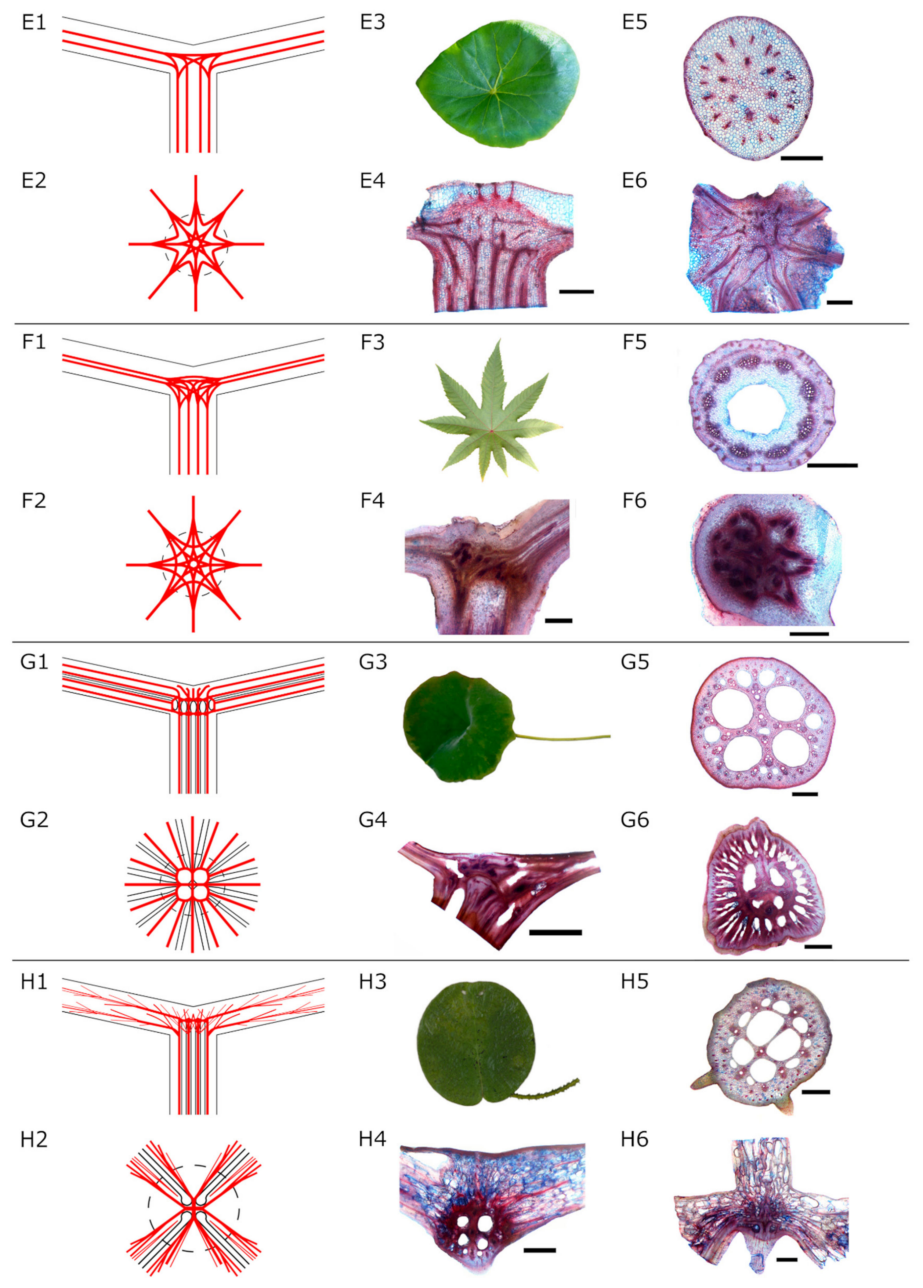
Types of strengthening structures in the petiole–lamina junction of peltate leaves (part 1). (**A**)—unbranched type (*Piper peltatum*, scale: 1 mm). (**B**)—monocot branching type (*Anthurium forgetii*, scale: 1 mm). (**C**)—ring-like structure (*Stephania delavayi*, scale: 500 µm). (**D**)—knot-like structure (*Tropaeolum tuberosum*, scale: (**D4**,**5**)—500 µm, (**D6**)—1 mm). (**E**)—net-like structure, intensity 1 (*Begonia nelumbiifolia*, scale: (**E4**), (**E6**)—2 mm, (**E5**)—1 mm). (**F**)—net-like structure, intensity 2 (*Ricinus communis*, scale: 1 mm). (**G**)—water plant radial branching type, (*Nelumbo nucifera*, scale: (**G4**)—5 mm, (**G5**)—1 mm, (**G6**)—2 mm). (**H**)—water plant diffuse branching type (*Victoria cruziana*, scale: (**H4**), (**H6**)—2 mm, (**H5**)—1 mm). 1—simplified illustration of strengthening structure type, longitudinal section of petiole and transition zone. 2—simplified illustration of strengthening structure type, top view of petiole (dashed line) and transition zone. 3—leaf morphology of peltate representative. 4—longitudinal section of the transition zone. 5—cross section of the petiole. 6—cross section of the transition zone.

The leaf anatomy of 41 peltate leaved plant species from 18 different families was studied and categorised (Figure 2). For reasons of clarity, per family a maximum of two species were selected. Profiles of the excluded taxa can be found in the Supplementary Materials.

#### 3.2.1. ANA Grade

##### Nymphaeaceae Salisbury

*Euryale ferox* Salisb.—This species, also named Foxnut, is often cultivated for its seeds and fruits. Habit: rhizomatous aquatic plant with annual leaves; large round leaf plates (1.3–2.7 m) on the surface, prickly on the top and bottom; under water non-spiny cordiform leaves of 4–10 cm; leaves abaxial dark violet, adaxial green, peltate, central petiole insertion; petiole covered with spines; flower up to 5 cm in diameter; petals elongated lanceolate, purple–violet exterior and white interior. Distribution: native in India, China, Japan and some countries in Southeast Asia. Ecology: lakes and ponds [30,38,39]. Petiole anatomy: large aerenchyma tubes, scattered vascular bundles (number of vascular bundles: 42), layers of collenchyma tissue below epidermis. Anatomy of the transition zone: parallel fibre strands from the petiole branch out in the transition zone forming a diffuse net of fibres in different sizes, fibres running into the lamina follow main veins and become increasingly finer, air channels are visible. Additional data: see Table 2.

*Victoria cruziana* Orb.—It is cultivated as an ornamental plant. Habit: rhizomatous, ground rooted aquatic herb with annual branches and leaves; all parts prickly except upper side of the leaves and most surfaces of the flowers; leaves bright greenish, up to 2 m wide, vertically oriented leaf margins (20 cm high); lily pads emerge from a rhizome by a long, flexible petiole; flowers large, floating, first white, after pollination turn to light pink, anchored by a long stalk arising from the rhizome. Ecology: grows in slowly moving and shallow water. Distribution: Northeast Argentina, Paraguay and Bolivia [30]. Petiole anatomy: large aerenchyma tubes, scattered vascular bundles (number of vascular bundles: 12), layers of collenchyma tissue below epidermis (Figure 2H5). Anatomy of the transition zone: parallel fibre strands from the petiole branch out in the transition zone forming a diffuse net of fibres in different sizes, fibres running into the lamina follow main veins and become increasingly finer, air channels are visible (Figure 2H4,H6). Additional data: see Table 2.

Additional studied species: *Cabomba aquatica* Aubl., *Nymphaea colorata* Peter, *Nymphaea lotus* L., *Victoria amazonica* (Poepp.) J.C. Sowerby.

**Table 2:** Leaf characteristics of peltate plant species. The number of petiole vascular bundles (v.b.), ratio of strengthening tissue (only lignified tissue) in % in petiole cross section, strengthening structure, the ratio of petiole area to lamina area in % and water content in % of petiole, lamina in total, intercostal areas and venation of 41 peltate species were analysed

Species	Number of v.b.	Proportion of Strength. Tissue (in Petiole, %)	Strengthening Structure	Sample Size (n)	Petiole Area/Lamina Area (%)	Water Content Petiole (%)	Water Content Lamina (%)	Water Content Intercostal (%)	Water Content Venation (%)
*Alocasia longiloba*	53	2.7	Sb						
*Amorphophallus konjac*	180	10.3	Ub						
*Anthurium forgetii*	60	9.7	Sb						
*Begonia kellermanii*	11	5.4	Ns1	5	0.14	99.3	98.7	98.9	95.9
*Begonia nelumbiifolia*	34	8.0	Ns1	4	0.06	95.2	89.7	89.7	89.4
*Begonia peltata*	24	4.2	Ns1						
*Begonia sudjanae*				4	0.08	94.8	92.3	92.4	92.2
*Cabomba aquatica*	2	1.1	?	5	0.81	93.4	85.4	85.1	88.3
*Caladium* hybrid	50	3.3	Sb	3	0.04	94.5	87.6	86.4	91.8
*Cecropia peltata*	23	11.1	Ns2	8	0.03	89.6	83.6	78.7	90.6
*Colocasia esculenta*	90	4.1	Sb	2	0.05	95.6	89.8	88.8	92.2
*Darmera peltata*	24	8.8	Ub	2	0.12	88.9	83.7	82.6	87.1
*Euryale ferox*	42	6.4	Wdb	5	0.28	96.8	93.8	92.0	95.3
*Hernandia nymphaeifolia*	11	8.5	Sb	6	0.07	88.5	81.1	80.2	88.5
*Hydrocotyle vulgaris*	3	2.2	Ks	4	0.30	89.7	84.9	84.7	85.3
*Jatropha podagrica*	10	10.8	Ns2	2	0.08				
*Kalanchoe nyikae*	3	3.1	Sb						
*Macaranga tanarius*	27	18.8	Ns2	3	0.04	77.5	76.9	76.8	77.0
*Marsilea strigosa*	1	3.9	Sb						
*Nelumbo nucifera*	63	19.3	Wrb	5	0.09	91.0	88.0	87.5	89.2
*Nymphaea colorata*	21	5.7	Wrb						
*Nymphaea lotus*				1	0.10	97.3	91.5	90.9	92.4
*Oxalis bowiei*	7	3.0	Ub						
*Passiflora coriacea*	6	13.2	Ub	2	0.08	85.6	86.2	86.1	86.6
*Peperomia argyreia*	8	1.5	Sb						
*Peperomia cyclaminoides*	6	1.1	Sb	2	0.79	96.1	94.1	94.1	94.1
*Peperomia sodiroi*	7	4.3	Sb	3	0.25	93.5	92.9	92.6	89.8
*Perichasma laetificata*	8	18.8	Rs						
*Pilea peperomioides*	6	10.7	Ub	7	0.16	95.0	95.1	95.1	95.5
*Piper peltatum*	13	8.2	Ub	3	0.03	91.7	85.0	83.8	87.8
*Podophyllum* hybrid	15	12.5	Ns1						
*Remusatia vivipara*	28	3.7	Sb	5	0.08	95.4	87.7	86.7	90.6
*Ricinus communis*	9	13.4	Ns2	4	0.05	86.4	77.6	76.7	81.0
*Rodgersia podophylla*	63	7.6	Ub	2	0.04	89.1	85.9	80.6	88.4
*Schefflera arboricola*	39	28.5	Ns1	1	0.05	90.6	75.8	75.6	76.7
*Stephania delavayi*	8	12.1	Rs	6	0.05	91.3	79.9	78.7	84.6
*Syneilesis palmata*	30	9.0	Sb	1	0.08				
*Tropaeolum tuberosum*	7	12.8	Ks	3	0.08	92.2	85.8	85.4	87.7
*Umbilicus rupestris*	8	6.8	Ub	1	0.44	97.5	97.0	97.0	96.9
*Victoria amazonica*	90	11.1	Wdb						
*Victoria cruziana*	12	7.9	Wdb	1	0.08				

**Figure 3:**
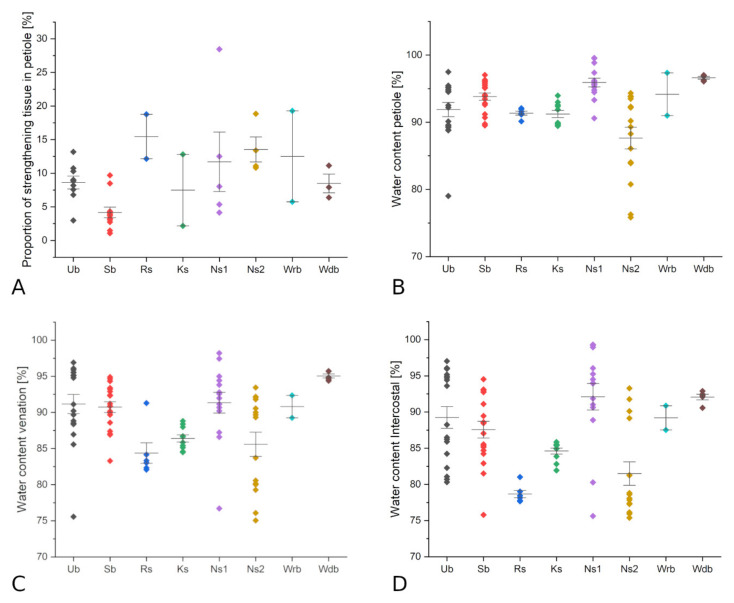
Proportion of (lignified) strengthening tissue in petiole (**A**) and water content of petiole (**B**), venation (**C**) and intercostal areas (**D**) in % of the different types of strengthening structures in peltate leaves (with mean and standard error). Abbreviations for strengthening structures: Ub—unbranched, Sb—simple branching, Ns1—net-like structure, intensity 1, Ns2—net-like structure, intensity 2, Ks—knot-like structure, Rs—ring-like structure, Wdb—water plant diffuse branching type, Wrb—water plant radial branching type.

Abbreviations for strengthening structures: Ub—unbranched, Sb—simple branching, Ns1—net-like structure, intensity 1, Ns2—net-like structure, intensity 2, Ks—knot-like structure, Rs—ring-like structure, Wdb—water plant diffuse branching type, Wrb—water plant radial branching type). Data shown in Figure 3A–D.

#### 3.2.2. Magnoliids

##### Hernandiaceae Blume

*Hernandia nymphaeifolia* (C. Presl) Kubitzki—Habit: tree or shrub, height: 3–20 m, spreading crown; petiole width: 1–3 cm, petiole length: 5–17 cm; leaves: 6–22 cm long, glabrous, peltate, ovate, entire margins, leaf base broadly rounded to rarely subcordate, leaf tip hastate, upper surface shiny green with eight to nine nerves; inflorescences with white- or cream-coloured flowers. Distribution: Southeast Asia, northern Australia and Madagascar. Ecology: sparse forests or bushland near sea level [30,40]. Petiole anatomy: circular arranged vascular bundles with sclerenchymatic caps on peripheral side (number of vascular bundles: 11), several layers of collenchyma under the epidermis. Anatomy of the transition zone: branching of some fibre strands, some fibre strands run unbranched into the apex of the leaf, fibre strands form a net-like structure in the transition zone and merge into large bundles following the leaf veins. Additional data: see Table 2.

##### Piperaceae Giseke

*Peperomia sodiroi* C. DC.—Habit: perennial, herbaceous, erect plant; stem glabrous; leaves oval, peltate, round at the base, tapered at the apical end, 5 cm wide, up to 6.5 cm long; petiole up to 9 cm long. Distribution: subtropical regions in Ecuador [30,41]. Petiole anatomy: circular arranged vascular bundles (number of vascular bundles: seven). Anatomy of the transition zone: fibre strands from the petiole spread out into lamina branching out minimally, forming a loose net-like structure and merging into large bundles following the leaf veins. Additional data: see Table 2.

*Piper peltatum*L.—Habit: shrub or subshrub, herbaceous, 0.5–2 m high; stem sparsely pilose or glabrous; leaves roundish-cordate, peltate, 15–30 cm wide, 20–40 cm long, apex pointed, base rounded to subcordate, glabrous, 10–15 veins radiating from the base of the petiole; petiole insertion point about a quarter to a third of the leaf length from the base, petioles 10–26 cm long, glabrous. Distribution: Mexico to Tropical America. Ecology: humid places at edges of meadows, forests, roadsides, stream edges, 0–500 (–800) m [42,43]. Petiole anatomy: circular arranged vascular bundles (number of vascular bundles: 13) around mucilage canal, collenchyma tissue below epidermis (Figure 2A5). Anatomy of the transition zone: fibre strands from the petiole spread unbranched into lamina (Figure 2A4,A6). Additional data: see Table 2.

Further studied species: *Peperomia argyreia* (Miq.) E. Morren., *Peperomia cyclaminoides* A.W. Hill.

#### 3.2.3. Monocotyledons

##### Araceae Jussieu

*Amorphophallus konjac* K. Koch—Habit: slightly glossy, depressed globose tuber, approx. 30 cm in diameter; in vegetative phase producing a single leaf, base colour of the petiole dirty pink, covered with dark green and smaller white dots (100 cm × 8 cm), lamina forms leaflets, strongly divided up to 2 m in diameter, leaflets (3–10 cm × 2–6 cm) adaxially dull green, elliptically tapering; inflorescence mostly long-stemmed, up to 110 cm × 5 cm in size, at the base dirty pale-brownish with black–green spots or dirty pale-white grey with some scattered black–green spots, mottled purple–red at the edge, spadix during female anthesis produces strong smell of rotting meat and small, clear, slightly viscous droplets, flower time April. Distribution: China (Yunnan), introduced to Korea, Philippines, Thailand, Tibet, Vietnam, other Chinese regions. Ecology: glades or forest margins and thickets, in secondary forests, altitude 200–3000 m [30,44]. Petiole anatomy: scattered vascular bundles (number of vascular bundles: 180), collenchyma strands near the epidermis. Anatomy of the transition zone: fibre strands from the petiole spread unbranched into petiolules of the leaflets. Additional data: see Table 2.

*Anthurium forgetii* N.E.Br.—Habit: small erect herb, up to 38 cm high; petioles 15–25 cm long, erect, glabrous, leaves sagging, 25–35 cm long, 15–22 cm wide, peltate to elliptic–ovate, dark green, leaf margin entire, apex pointed, rounded at the base, upper side with a velvety sheen and pale green prominent nerves, underside pale green, sometimes with a slight violet tinge. Distribution: Colombia. Ecology: Andes, altitude 1100 m [30]. Petiole anatomy: central scattered vascular bundles, peripheral ring of vascular bundles (number of vascular bundles: 60), both with sclerenchyma bundle sheaths, sclerenchyma associated with peripheral vascular bundles forms closed ring (Figure 2B5). Anatomy of the transition zone: fibre strands from the petiole branch and proceed into leaf veins, some remain unbranched and follow the main vein (Figure 2B4,B6). Additional data: see Table 2.

Further studied species: *Alocasia longiloba* Miq., *Caladium* hybrid, *Remusatia vivipara* (Roxb.) Schott, *Colocasia esculenta* (L.) Schott.

#### 3.2.4. Eudicotyledons

##### Araliaceae Jussieu

*Hydrocotyle vulgaris*L.—Habit: stoloniferous perennial plant; leaf lamina 1.5–4 cm in diameter, peltate, slightly notched, without incision at the base, petioles 3–18 cm long, about 1 mm thick, central leaf insertion. Distribution: generally oceanically distributed, Azores, Europe to Mediterranean. Ecology: fens, fen-meadows and swamps, dunes and damp hollows, occasionally in deeper water, also in wet heaths and ditches, generally calcifugal [30,45,46,47]. Petiole anatomy: three central vascular bundles surrounded by parenchyma. Anatomy of the transition zone: fibre strands from the petiole approach dense structure in transition zone, then separate into eight strands running into the leaf veins. Additional data: see Table 2.

*Schefflera arboricola* (Hayata) Merr.—Cultivated as an ornamental plant and used medicinally. Habit: shrub or rarely climbing up to 4 m height; petioles 6–20 cm long, pinnate leaves, leaves divide into 5–9 obovate–oblong to oblong or elliptical leaflets with 6–10 cm length and 1.5–3.5 cm width, leaflets are subleathery, glabrous on both sides, margins entire; inflorescence consists of terminal, glabrous corymbs with 3–8 cm. Distribution: Hainan (China), Taiwan, introduced in Florida (USA). Ecology: on banks of streams, in humid forests, altitude up to 900 m [30,48]. Petiole anatomy: vascular bundles arranged in two rings (number of vascular bundles: 39), vascular bundles in the inner ring arranged irregularly, sclerenchymatic bundle sheaths with large fibre caps envelop outer ring, layer of collenchyma under epidermis. Anatomy of the transition zone: transition zone between petiole and petiolules seems to be the predetermined breaking point, fibre strands branch out and then merge into petiolules. Additional data: see Table 2.

##### Asteraceae Bercht. and J. Presl

*Syneilesis palmata* (Thunb.) Maxim.—Habit: herbaceous perennial, robust, 60–75 cm height; leaf lamina up to 50 cm in diameter, peltate, palmately parted (7–9 lobes), ± orbicular, long petiole; flowers white, flowering time: summer. Distribution: Japan, Korea [30,49,50]. Petiole anatomy: vascular bundles arranged in 1–2 irregular rings, somewhat scattered, larger bundles peripheral, two central vascular bundles (number of vascular bundles: 30), large sclerenchymatic caps, several layers of collenchymatic tissue under epidermis. Anatomy of the transition zone: fibre strands from the petiole run mostly unbranched, with a few bundles splitting, through the transition zone into the lamina. Additional data: see Table 2.

##### Begoniaceae C. Agardh

*Begonia peltata* Otto and A. Dietr.—Habit: shrub with an erect stem; petioles 8–15 cm long, leaves approx. 15 cm long, taper from a broad base to a narrow tip, asymmetric, ovoid, peltate with an eccentric petiole insertion, palmately veined with just a few branches, petiole and leaves green with velutinous pubescence. Distribution: South Mexico to Honduras. Ecology: drought adapted plant, grows in dry canyons, in Guatemala it grows on the ground/on rocks in wet forests and thickets [30,51,52,53]. Petiole anatomy: vascular bundles arranged in two concentric rings (number of vascular bundles: 24), few layers of collenchymatic tissue below epidermis (Figure 2E5). Anatomy of the transition zone: fibre strands from petiole branch out in the transition zone forming a net and merge into fibre bundles, proceeding into the leaf veins (Figure 2E4,E6). Additional data: see Table 2.

*Begonia nelumbiifolia* Schltdl. and Cham—Habit: herbaceous perennial plant; leaf lamina glabrous, broadly ovate to peltate, base rounded, apex pointed, 15–40 cm long, 11–32 cm wide, seven to nine main nerves, petioles 11–63 cm long, 2–7 mm wide, hirsute; inflorescence rich in flowers, 7–28 cm in diameter, flowering time December to May. Distribution: Mexico to Colombia, introduced in Cuba, Dominican Republic and Puerto Rico. Ecology: altitude 100–400 m, along roadside verge, in Guatemala it grows in humid thickets and forests up to 1650 m [18,30,54,55,56]. Petiole anatomy: vascular bundles arranged in two concentric rings (number of vascular bundles: 34), few layers of collenchymatic tissue below epidermis. Anatomy of the transition zone: fibre strands from petiole branch out in the transition zone forming a net and merge into fibre bundles extending into the leaf veins. Additional data: see Table 2.

Further studied species: *Begonia kellermanii* C. DC., *Begonia sudjanae* C.-A. Jansson

##### Berberidaceae Jussieu

*Podophyllum*L.—Habit: deciduous, herbaceous perennials, 20–60 cm high, one leaf or flowering shoot per year emerge from a rhizome; leaves 10–38 cm, very diverse, but with a simple construction, petiole more or less centrally attached to the variably lobed lamina, leaf margins entire or serrated, venation palmate; inflorescence terminal, flowers 30–55 mm, six to nine petals, white or pink. Distribution: temperate eastern states of the USA, many countries in Southeast Asia including India and Japan [30,57].

*Podophyllum* hybrid—Habit: 60 cm high, peltate leaves, 10–40 cm, petiole green, almost centrally attached to the lamina, lamina palmatifid, two to five pointed lobes, at the base not lobed, light and dark green speckled, leaf margin entire. Petiole anatomy: vascular bundles arranged in a ring and central vascular bundles (number of vascular bundles: 15). Anatomy of the transition zone: fibre bundles branch out into smaller strands in the transition zone forming a net of fibre strands, merging into larger fibre bundles following the leaf veins. Additional data: see Table 2.

##### Crassulaceae J. Saint-Hilaire

*Kalanchoe nyikae* Engl.—Habit: perennial, erect or decumbent at the base, glabrous, glaucous plant, 60 cm to 2 m high; basal leaves almost circular, cuneate or slightly cordate at the base, margins entire, middle leaves peltate or auriculate, lamina 7–8 cm long, 6–7 cm wide, petioles 3–10 cm long, upper leaves lanceolate; inflorescences paniculate cymes up to 35 cm. Distribution: Kenya, Tanzania [30,58]. Petiole anatomy: several differently sized vascular bundles (number of vascular bundles: at least three). Anatomy of the transition zone: some fibre strands branch out; others extend into the lamina without branching. Additional data: see Table 2.

*Umbilicus rupestris* Dandy—Habit: geophyte, erect, glabrous, usually unbranched, 10–50 cm high; leaves crenate, roundish-peltate, 1.5–7 cm in diameter, central petiole insertion, petiole 4–25 cm long; inflorescence occupies 70%–90% of the stem length, racemose, flowers white, yellowish or reddish, often spotted, usually drooping. Distribution: Britain Islands, Mediterranean area, Arabian Peninsula. Ecology: temporarily humid locations, shady and humid growing places such as rocks and walls [18,30,58,59,60]. Petiole anatomy: smaller and larger vascular bundles arranged in one ring (number of vascular bundles: eight). Anatomy of the transition zone: vascular bundles from the petiole approach transition zone, run unbranched towards the lamina merging into larger bundles following the main veins. Additional data: see Table 2.

##### Eurphorbiaceae Jussieu

*Macaranga tanarius* (L.) Müll. Arg.—Habit: shrub to medium–high tree, evergreen; leaves alternate, simple, peltate, lamina circular to ovate, 8–32 cm × 5–28 cm, 9 radiating veins, margins entire, base rounded, apex acuminate, upper surface green, glabrous, lower surface paler, sometimes glabrous or occupied with sessile glands and simple hairs, petioles same as leaf length. Distribution: Southeast Asia and eastern Australia. Ecology: subtropical and tropical regions, common as a pioneer species in rainforest along the coast or cleared areas [30,61]. Petiole anatomy: vascular bundles arranged in two concentric rings and a central bundle (number of vascular bundles: 27), sclerenchyma associated with outer ring of vascular bundles, ring of sclerenchymatic tissue a few layers below epidermis, laticifers present. Anatomy of the transition zone: fibre strands from the petiole form a highly branched, dense network in the transition zone, then merge into larger bundles following the leaf veins into the lamina. Additional data: see Table 2.

*Ricinus communis*L.—This species is used in traditional medicine since the ancient Egyptian times. The oil of the seeds (castor oil, wonder oil) is used worldwide for a variety of medicinal purposes. Habit: shrubs or trees in tropical and subtropical regions up to 10 m high, in cooler regions often annual herbs 1–4 m in height; older stems brown or green, younger parts glaucous, annual plants often reddish or purplish; alternate leaves 30–50 cm, sometimes up to 100 cm large, papery, green, leaf shape palmately split with six to 11 ovate lobes, leaf margin serrated with unequal sized tips; inflorescence, up to 30 cm, fruit capsules dark red, seeds shiny brown, 8–11 mm; flowering and fruiting time throughout the year (tropical zones) or summer/late fall (temperate zones). Distribution: north-eastern Africa, cultivated as an ornamental and a medicine plant throughout subtropical and temperate zones worldwide. Ecology: sunny, warm places, altitude 0–700 m, in some regions up to 2300 m, all kinds of habitats usually on rich soil, resistant to termites and drought [30,62,63,64]. Petiole anatomy: one ring of vascular bundles (number of vascular bundles: nine), ring of collenchymatic tissue below epidermis, central medullary canal, laticifers present (Figure 2F5). Anatomy of the transition zone: fibre strands from the petiole form a highly branched, dense network of fibre strands in the transition zone, then merging into larger bundles following the leaf veins into the lamina (Figure 2F4,F6)). Additional data: see Table 2.

Further studied species: *Jatropha podagrica* Hook.

##### Menispermaceae Jussieu

*Perichasma laetificata* Miers—Habit: climbing plant; leaves simple, petiole 9–12 cm long, hirsute, petiole insertion 4–4.5 cm from base of the lamina, lamina peltate, ovate, 17.5–22 cm long, 13–16 cm broad, leaf margin undulate, rounded at the base, bristly underneath, upper surface glabrous, palmately nerved, eight to nine nerves. Distribution: western Central Africa, from Nigeria to Angola [30,65]. Petiole anatomy: one ring of vascular bundles (number of vascular bundles: eight), ± closed ring of sclerenchyma fibres around vascular bundles. Anatomy of the transition zone: parallel fibre strands from petiole form a closed ring structure in transition zone, from this ring, single fibre bundles branch into leaf veins. Additional data: see Table 2.

*Stephania delavayi* Diels—Habit: slender herbaceous plant, vines, 1–2 m high, rarely branching; petiole and lamina mostly of similar length 3–7 cm long, leaf width ± equal to leaf length, leaf margins entire, lamina peltate, triangularly round, leaf base and tip blunt, both surfaces glabrous, abaxially pink–green and adaxially pale green to dark green, leaf thin papery, palmate, nine to 10-veined. Distribution: South and Central China and Myanmar. Ecology: in shrubland along fences and roadsides [30,40]. Petiole anatomy: one ring of vascular bundles (number of vascular bundles: eight), indistinct sclerenchyma fibres around vascular bundles, collenchymatic tissue below epidermis (Figure 2C5). Anatomy of the transition zone: parallel fibre strands from petiole form a closed ring structure in transition zone, from this ring, single fibre bundles branch into leaf veins (Figure 2C4,C6). Additional data: see Table 2.

Further studied species: *Stephania venosa* (Blume) Spreng.

##### Nelumbonaceae A. Richard

*Nelumbo nucifera* Gaertn.—The Indian lotus is cultivated as an ornamental plant and for its edible rhizomes and seeds. The seeds remain viable for several hundred years under certain conditions. Habit: perennial, rhizome forming, aquatic plant; petiole length 2 m and more, leaf stalks bare or papillose, very hard, leaves bluish green on the upper side, whitish green underneath, circular to oval shape, leaves entire, forming a slight funnel, 25–90 cm in diameter, very thin, water-repellent; pedicels of the flowers are longer than petioles, flowers pink or whitish yellow, 10–23 cm in diameter. Distribution: Southeast Asia, Southeast Europe, Australia, eastern USA. Ecology: temperate to tropical climates, different wetland habitats (ponds, lakes, lagoons, swamps and flood plains) with a depth of up to 2.5 m, altitude 400 m [30,38,57]. Petiole anatomy: scattered vascular bundles (number of vascular bundles: 63), large aerenchyma tubes, collenchyma and partly lignified tissue below epidermis (Figure 2G5). Anatomy of the transition zone: fibre bundles run parallel to the air channels into the transition zone, forming a symmetric net surrounding the cavities, eventually extending radially into the lamina (Figure 2G4,G6). Additional data: see Table 2.

##### Oxalidaceae R. Brown

*Oxalis bowiei* Aiton ex G. Don—also commonly known as Bowie’s woodsorrel. Habit: bulbous perennial, stemless, pubescent; leaves basal, petiole 6–16 cm long, glandular pubescent, three leaflets, 3–6 cm long, obtuse, lobed, glandular pubescent, upper surface green, lower surface green to purplish; peduncles as long as leaves, inflorescences cymes, four to 12 flowers, flowers red, pink to deep rose pink. Distribution: Cape region of South Africa, introduced in California (USA), Korea and Western Australia. Ecology: disturbed areas, 300 m, occasionally grown horticulturally in mild temperate climates [30,63,66,67]. Petiole anatomy: central closed cylinder of vascular bundles (number of vascular bundles: seven) surrounded by thin layer of collenchyma, then parenchyma. Anatomy of the transition zone: cylinder of vascular bundles separate into three broad fibre strands merging into veins of the three leaflets. Additional data: see Table 2.

##### Passifloraceae Juss. ex Roussel

*Passiflora coricaea* Juss.—Habit: slender, climbing plant, perennial vine, 2–8 m or more, sparsely pilose on petioles, leaves, stems and stipules; petioles ca. 1–4 cm long, lamina elliptical, ca. 3–6 cm long, ca. 6–19 cm wide, with two or three lobes, petiole insertion point close to leaf margin, two lateral lobes 3–10 cm long, 2–7 cm wide, elliptical, pointed to weakly pointed, central lobe elliptical to obovate or present only as a blunt tip, leaf margins entire, three primary veins branching above the base; flowering stems (1–3 mm in diameter) greenish yellow to reddish purple, terete to somewhat compressed, base woody and cork-covered, flowers borne in leaf axils or in inflorescences, inflorescences 2.5–6.5 cm long, rarely up to 12 cm. Distribution: northern South America, Central America. Ecology: in shrubs and small trees in secondary successional areas, along edges of moist tropical forests, near rivers and streams, along the seashore, 0–1500 m [30,68]. Petiole anatomy: one central vascular bundle, five vascular bundles ± in a circle (three bundles in an arch, two bundles on the opposite side), layer of collenchymatic tissue below epidermis. Anatomy of the transition zone: fibre strands spread into lamina without branching. Additional data: see Table 2.

##### Saxifragaceae Jussieu

*Darmera peltata* (Torr. ex Benth.) Voss—Habit: herb with a perennial rhizome (1–5 cm in diameter); leaves emerging directly from rhizome, petiole 20–150 cm long, lamina 10–60 cm wide, sometimes up to 90 cm, peltate, centrally attached to petiole, leaf margins irregularly serrated, six to 15 lobed, upper side green, underside pale green, both sides ± glandular; flowering stems appear before leaves, erect, 30–100 cm high, sometimes up to 150 cm, inflorescences compound cymes, 30–150 cm large, 60 to 75 flowers, green to pinkish purple or white to rose, flowering time between April and July. Distribution: California and Oregon in the U.S., introduced in Czech Republic, France, Great Britain and Ireland. Ecology: between rocks in and around streams, altitude 30–1800 m [30,69]. Petiole anatomy: peripheral ring of vascular bundles and scattered central vascular bundles (number of vascular bundles: 24). Anatomy of the transition zone: fibre strands spread into the individual veins of the lamina without branching. Additional data: see Table 2.

*Rodgersia podophylla* A. Gray—Habit: perennial herb, 60–100 cm height; rhizome forming; stem glabrous; petiole 15–30 cm long, pilose, leaves palmately compound, five leaflets, obovate, 15–30 cm long, 10–25 cm wide, lobed apex (three to five lobes), adaxially glabrous, abaxially pilose along the veins, serrated margin, apex acuminate, cauline leaves smaller than basal leaves; many flowered inflorescence, sepals white, petals absent, flowering time June to July. Distribution: Japan, Korea, China, introduced into Czechoslovakia, Great Britain, Norway. Ecology: shaded slopes [30,70]. Petiole anatomy: vascular bundles arranged in several rings (number of vascular bundles: 63), sclerenchymatic bundle caps visible, sclerenchymatic caps surrounding outer ring of vascular bundles form an almost closed ring. Anatomy of the transition zone: fibre strands from the petiole run mostly unbranched, only few branching bundles, through the transition zone into leaflets. Additional data: see Table 2.

##### Tropaeolaceae Juss. ex de Candolle

*Tropaeolum tuberosum* Ruiz. and Pav.—This species, also known as “Mashua”, is used as a tuber crop plant in the cool–temperate Andes. Habit: herbaceous perennial, > 2 m high, 1 m in diameter; tuber white to yellow, occasionally red, flesh yellow; leaves peltate, three to five lobed, 4–6 cm long, 5–7 cm broad, palmate venation petioles glabrous, reddish, 4–20 cm long, plant uses long leaf stalks as tactile petioles for climbing; flowers long-stalked, solitary, variable in colour such as orange, yellow or scarlet. Distribution: higher Andes of Peru, Colombia, Ecuador and Bolivia, cultivated in parts of northern Argentina and Chile, also introduced in New Zealand. Ecology: altitude 2400–4300 m, annual mean temperatures 8–11°C [71,72,73]. Petiole anatomy: ring consisting of seven vascular bundles, distinct layers of collenchyma underneath the epidermis (Figure 2D5). Anatomy of the transition zone: fibre strands unite into dense, knot-like structure, individual fibre strands extend from there into leaf veins (Figure 2D4,D6). Additional data: see Table 2.

##### Urticaceae Jussieu

*Cecropia peltata* L.—Habit: tree, 15–25 m; leaves 10–60 cm in length and width, palmately divided into seven to 11 lobes, upper surface scaly, lower side hairy, petioles 20–50 cm long. Distribution: Mexico to northern South America, introduced in Africa and Asia. Ecology: wet to deciduous forests, as secondary vegetation, in pastures and roadsides, altitude 0–1800 m [30,74]. Petiole anatomy: peripheral ring of vascular bundles (number of vascular bundles: 23), layers of collenchyma below epidermis. Anatomy of the transition zone: fibre strands from the petiole form a highly branched, dense network, merging into larger bundles following the leaf veins into the lamina. Additional data: see Table 2.

*Pilea peperomioides* Diels—Habit: herbaceous perennial plant, erect or ascending, wild plants up to 23 cm, in cultivation up to 60 cm in height; robust stems, greenish to dark brownish; leaves spirally arranged, lamina pale green underneath, upper side green, somewhat succulent, three veins, three to four lateral veins on each side, petioles 2–17 cm long, leaves peltate, elliptic, 4–7 cm long and wide, base rounded, apex round or obtuse; inflorescence solitary, 18–28 cm, flowers pinkish cream, in clusters. Distribution: China (West Yunnan and Southwest Sichuan), introduced in Belgium. Ecology: on shady, damp rocks in forests or cliff-ledges, on humus-covered boulders, altitude 1500–3000 m, very rare in the wild, possibly endangered in its native habitat [30,75,76]. Petiole anatomy: few centrally arranged vascular bundles surrounded by parenchymatic tissue (number of vascular bundles: six). Anatomy of the transition zone: fibre strands spread out in transition zone, proceed unbranched into lamina. Additional data: see Table 2.

#### 3.2.5. Polypodiopsida

##### Marsileaceae Mirbel

*Marsilea strigosa* Willd.—Habit: aquatic, heterosporous fern with long stolons, thick creeping subterranean rhizome; leaves with four green, pubescent leaflets, 0.8–1.5 cm in diameter, petioles slender, 10–15 cm long, two types of leaf variants: (1) floating leaves, glabrous, with long slender petiole, (2) free standing leaves, rigid petiole, tomentose blade; reproduces clonally and sexually, producing stoloniferous structures and sporocarps, brown, sessile, spherical sporocarps (0.5–0.3 cm diameter) at the base of petioles. Distribution: Afghanistan, Algeria, Baleares, eastern European Russia, Egypt, France, Italy, Kazakhstan, Morocco, Portugal. Ecology: short life cycle, Mediterranean temporary pools, among amphibious vegetation on various non-calcareous substrates [30,77]. Petiole anatomy: vascular tissue in central cylinder. Anatomy of the transition zone: cylinder separates into several fibre strands that run into the leaflets. Additional data: see Table 2.

### 3.3. Classification of Strengthening Structures in the Petiole–Lamina Transition Zone

The microscopic examination of peltate leaves from different plant species revealed different types of transition zones formed by fibre strands at the joint between the lamina and petiole. The types vary from virtually absent or very simple to very complex structures, with gradual transitions in intensity as well as in the form and shape of the branching system. In total, seven different types with one subtype have been identified (including a simple unbranched type).

1. Unbranched type (A): The fibre strands in the petiole continue unbranched through the petiole–lamina junction into the lamina (Figure 2A). The strands follow the leaf veins.
2. Simple branching type (B): Some fibre strands in the petiole branch in the transition zone and then extend into the leaf veins while others proceed unbranched into the lamina (Figure 2B). The fibre strands branching in the transition zone can either run in into the same leaf vein of the lamina or follow a different direction into different leaf veins, forming a connection between, e.g., opposite sides of the lamina. All species with at least one visible branching fibre strand were assigned to this category.
3. Ring-like structure (Type C): The fibre strands from the petiole run parallel towards the petiole–lamina junction, then merge into a fibre ring without any visible branching. This ring is oriented parallel to the lamina and connects all the strands from the petiole with each other. Starting from the ring, the strands spread into the leaf veins (Figure 2C). This structure was found only in *Stephania* and *Perichasma*.
4. Knot-like structure (Type D): Isolated fibre strands from the petiole assemble to a dense knot-like structure in the transition zone. It is not possible to distinguish between individual fibre strands in this knot. A few stronger fibre strands spread out from these structures and continue into the lamina (Figure 2D).
5. Net-like structure (Type E and F): The individual fibre strands the petiole form a net-like structure in the transition zone. Individual fibre strands are still visible. Fibres branch and merge in different intensities, forming nets of different densities. Two subtypes were identified here: (1) slight to moderate branching (Figure 2E) and (2) intensive branching (Figure 2F) of the fibre strands. From this net, several fibre bundles spread out into the lamina following the larger veins.
6. Radially branching type (water plants, Type G): The fibre strands from the petiole are united in a dense structure with large fibre strands and big intercellular cavities for gas exchange. The fibre strands run parallel to the air channels branch in the transition zone and extend into the lamina in a radial pattern (Figure 2G).
7. Diffuse branching type (water plants, Type H): Parallel fibre strands from the petiole branch in the transition zone forming a diffuse net of fibres of different sizes (Figure 2H). Air channels are visible.

**Figure 4:**
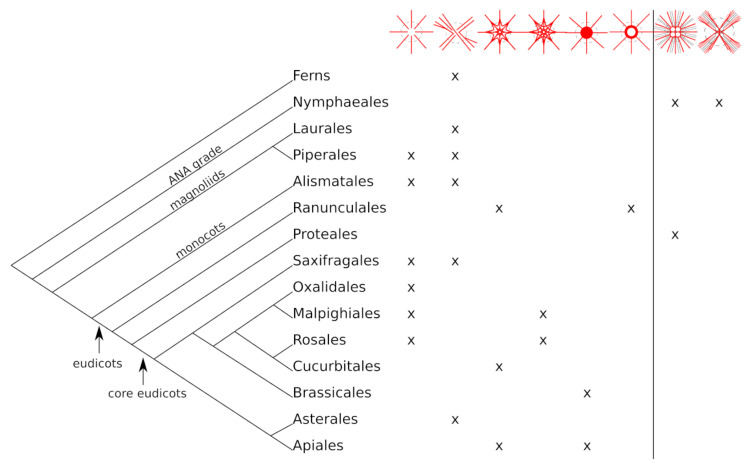
Distribution of the different strengthening structures in the petiole–lamina junction of peltate leaves within the plant kingdom. Strengthening structures (from left to right): unbranched, simple branching, net-like (intensities 1 and 2), knot-like, ring-like, radial (water plant), diffuse (water plant). The unbranched and simple branching type are the most common types found in six orders, followed by the net-like structure. All other structures only occur in one to two orders.

The unbranched and simple branching types are quite common among the analysed species. Eight species found in six orders throughout the plant kingdom, from magnoliids and monocots to eudicots, show no conspicuous strengthening structure in the joint of the petiole and lamina (Figure 4). The simple branching type occurs in twelve species and six orders with the Araceae (monocots) and Piperaceae (magnoliids) containing the majority of the twelve taxa (Araceae: five species, Piperaceae: three species). All five species from Araceae (five different genera) show this type of branching. The net-like structure could be found in five orders. While the slight to moderate branching intensity is found in Ranunculales (*Podophyllum*) and two core eudicot orders (Cucurbitales and Apiales, four species), the intensive branching is only present in two rosid orders (Malpighiales and Rosales, four species). The knot-like structure occurs in two orders from the core eudicots (Brassicales and Apiales, one species each). The ring-structure was only found in the Ranunculales, in the Menispermaceae. All three investigated species from the Menispermaceae show this strengthening structure in the petiole–lamina transition zone. The two branching types in water plants were found in Nymphaeales (four species). The radial branching type additionally appears in the Proteales (Nelumbonaceae, *Nelumbo nucifera*).

When comparing the proportion of lignified tissue in the petiole of the different types of strengthening structures, the unbranched, simple branching and water plant diffuse branching type seem to have lower amounts than the net-like structure, intensity 2 and the ring-like structure (Figure 3A). The water content in petiole, venation and intercostal areas shows a high variance in most categories. However, the net-like structure, intensity 2 and partly ring-like structure and knot-like structure seem to show lower water content than the other categories (Figure 3B–D).

## 4. Discussion

### 4.1. The Peltate Leaf Shape and Its Representatives in the Plant Kingdom

Since the lists published by de Candolle (1899), Troll (1932) and Ebel (1998), the distribution of the peltate leaf shape in the plant kingdom has not been focus of many studies [18,19,24]. This study aimed to provide an up to date, comprehensive list of peltate plant species including information about morphology, anatomy, distribution, and habitat. The peltate leaf shape was found in all clades of angiosperms including basal angiosperms in the ANA grade (Table 1). The order Nymphaeales with the Nymphaeaceae and Cabombaceae contains peltate aquatic plants differing in shape and size, but the leaves, without exception, are floating. While most of the peltate leaves of the genus *Nelumbo* in the Nelumbonaceae are raised above the water surface, *Nelumbo* also forms floating leaves [38,57]. Ebel (1998) noted that, in these two groups, the ratio of peltate to non-peltate genera is shifted in favour of the peltate leaved type [18]. In the entire clade of magnoliids, only two families with peltate species have been identified (Piperaceae, Hernandiaceae). In the Piperaceae, especially in the species-rich genus *Peperomia* with about 1600 species [78], many peltate representatives could be found with a marginal to central petiole insertion point (Table 1). As Ebel (1998) already observed [18], the *Peperomia* has its main distribution area in the Neotropics.

Peltate leaves are unusual among monocotyledons, being virtually limited to the Araceae [18]. Ten genera with peltate leaved species could be determined, which is more when compared to the other investigated families. As land plants, they are mostly found in subtropical areas (Table 1). Ebel (1998) suggests that the peltation in Araceae is likely related to the leaf venation, which is pinnate or palmate instead of the typical parallel venation of monocots [18,79,80] and observed in all peltate leaved Araceae. *Arisaema* and *Amorphophallus* occupy a special position, since both are not only centrally peltate but also form pinnate leaves, while the remaining genera show an eccentric peltation and have a simple entire lamina.

Peltation is most widespread in eudicots (Table 1). However, the occurrence is not limited to a few orders or families, as in monocots and magnoliids. In each order, one to four families with up to nine genera contain species with peltate leaves (e.g., Euphorbiaceae, Asteraceae, Ranunculaceae, Gesneriaceae, Menispermaceae, Table 1).

Among the studied species, leaves with the eccentric peltation are more common compared to those with a central attachment of the petiole (Table 1). Genera rich in centrally peltate species (e.g., *Hydrocotyle*, *Umbilicus*, *Oxalis*, *Podophyllum* and several genera of the Saxifragaceae) are native to meridional and temperate floral zones (or the southern hemispheric equivalent). In the neotropical and large parts of the palaeotropical floral zones, centrally peltate leaves are not as common as eccentric peltate leaves, indicating a connection between central peltation and geographic distribution, but the data does not give any explanation for this. Species with central peltation in the tropical and subtropical zones are mainly restricted to Nymphaeaceae, Oxalidaceae and Araliaceae. For Nymphaeaceae, the central peltation and the circular shape of the leaves can be explained as an adaptation to floating on the water surface, but this does not explain the peltate leaves in terrestrial plants.

The great majority of species with peltate leaves are perennial herbs. Already, Ebel (1998) noted that most of the taxa with peltate leaves are rhizomatous, tuberous or stoloniferous perennials growing in wet, humid or alternately wet habitats [18]. While Troll (1932) rejected a correlation between peltation and ecology [19], Friedrich Ebel (1998) pointed towards a connection between peltation and certain chorological and habitat conditions [18]. Data from this study suggest that there may be relations between the peltate leaf shape and species distribution, habitat or phylogeny supporting Ebel’s findings. Nevertheless, the observations at this point can only be regarded as indications.

### 4.2. Supporting a Peltate Lamina—Different Adaptations in the Petiole–Lamina Junction

The anatomical analysis of the petiole–lamina transition of 41 peltate leaved species from 18 families revealed seven different types of strengthening structures.

This study focused on reinforcing elements consisting of xylem elements and sclerenchymatic fibres, which are mainly found as vascular bundle sheaths or sclerenchyma caps associated with the vascular bundles [2,3,17]. However, in addition to these fibres, other strengthening tissue, such as collenchyma but also epidermal cell walls or the turgor pressure of living tissues, especially the parenchyma contribute to mechanical stability of plants [1,14,15,81]. As measurements of strengthening tissue were conducted only on a small number of samples and for some categories only a small number of leaves were available, the conclusions made in the discussion should be seen as tendencies.

#### 4.2.1. Simple Structures in the Petiole–Lamina Junction—The Unbranched and Simple Branching Structure

The unbranched structure, Type A, is a most common type (eight species, Table 2) among the examined peltate leaves. Since the leaves with unbranched fibre strands (Figure 2A) at first glance seem to have developed a less effective strengthening structure in the transition zone from the petiole to lamina, a closer look at *Amorphophallus konjac* points towards an explanation how, in this case, a very large lamina with up to 2 m in diameter [44] is stabilised: a combination of a large number of vascular bundles (180) with lignified xylem, on the one hand, and layers of collenchyma tissue associated with the outer vascular bundles on the other hand. These layers of collenchyma are a common mechanical tissue in the Araceae [15]. Another possibility to hold up and stabilise the peltate lamina can be observed in *Umbilicus rupestris* and *Pilea peperomioides*, both forming more or less succulent leaves and growing in shady, damp habitats [58,76]. Both species show high values in water loss (over 95%) displaying the high water content of the succulent leaves, low to medium amount of sclerenchymatic strengthening tissue in the petiole (up to 10.7%, Table 2) and no collenchyma. This suggests that the stability of the lamina of both species is mainly ensured via the turgor pressure in the parenchymatic tissues.

The simple branching structure (Type B) is characterized by the branching of some fibre strands while others run into the lamina unbranched. In its simplicity, it is a common structure in the transition zone from the petiole to lamina with 12 species from six orders sorted into this category (Figure 4). The families and genera sorted in this category show different anatomical features, but all show this type of branching. Certainly, further subcategories could be defined with more in-depth research. With eight of the 12 taxa sorted into this category, the majority of the simple branched species can be found in the more basal orders of the angiosperms.

Characteristic for this group is the relatively low proportion of strengthening tissue (1–10%, Table 2 and Figure 3A) suggesting that these species, similar to the unbranched species, need other additional strategies to support the peltate lamina.

Most of the studied Araceae exhibit the simple branching type. All of them show high numbers of scattered vascular bundles in the petiole (30–90, Table 2) and associated layers of collenchyma tissue (common in Araceae [15]), which stabilise the partly very large leaves with small ratios of petiole area to lamina area (0.04%–0.07%). The medium to high water content (80–95%) indicate that these species also strongly depend on turgor pressure to support the peltate laminae. In the petiole–lamina junction, the cross-linking of opposite sides of the lamina could be observed. Some of the fibres branch with individual arms extending into different parts of the lamina (Figure 2B), providing extra stability.

All studied *Peperomia* species (Piperaceae) display the simple branching type as well. In contrast to Araceae, the *Peperomia* species show significantly lower numbers of vascular bundles in the petiole arranged in a circle (6–8, Table 2). The studied neotropical *Peperomia* species are very low growing herbaceous perennials with small leaf blades [30], display high ratios of petiole to lamina area (0.25% for *P. sodiroi*, Table 2) and a high water content (about 95%). Given the growth conditions, habit and the large petiole to leaf ratio, *Peperomia* obviously does not need a complex strengthening structure in the petiole–lamina junction but supports most of its own weight by turgor pressure.

#### 4.2.2. The Net-Like Branching Structure

The net-like structures (Types E and F) could be observed in several species from different genera and families. Due to the different intensities of branching and interconnecting of the fibre strands, this category is divided into two different subtypes, based on the same reticulate structure.

The slightly branched reticulate structure (Type E) is mainly found in *Begonia*. This mega-diverse genus exceeding 1800 accepted species (over 2000 species estimated) and a pantropical distribution shows a variety of growth forms including herbs, shrubs, acaulescent species, succulent or woody stems or lianoid species [51,82]. The leaves of *Begonia* show a great diversity as well as with several sections of the genus containing peltate species [51]. Although differing in shape, size and texture, the leaves of Begonia reveal a similar structure in the petiole cross sections with collenchyma underneath the epidermis and the lignified xylem elements as additional strengthening elements. The branching and crosslinking of the xylem fibre strands in the petiole–lamina junction ensure the stabilisation of the peltate lamina (Figure 2E). The low proportions of strengthening tissue (4%–8%, Table 2) and high water content of the leaf (90%–95%) again indicate that these species strongly depend on turgor pressure to support their laminae with collenchyma tissue as counterpart.

Leaves with strongly branched fibre strands forming a reticulate structure (Type F) are mainly found in shrubs and trees form Euphorbiaceae such as *Jatropha podagrica*, *Macaranga tanarius* and *Ricinus communis* (Table 2). Despite morphological differences, e.g., leaf shape and origin from three different continents, anatomical similarities are apparent in the peripheral circle of vascular bundles with distinct xylem tissue and layers of mechanical tissue (collenchyma or sclerenchyma) associated with the vascular bundles. *Macaranga tanarius* shows a closed ring of sclerenchyma tissue associated with the outer vascular bundles, apparent in the highest proportion of strengthening tissue (18.8%) when compared to *R. communis* (13.4%) and *J. podagrica* (10.8%) (Table 2 and Figure 3A). In comparison to the low intensity of net-like branching in *Begonia,* the intensive crosslinking in Euphorbiaceae, becomes apparent in the higher ratio of strengthening tissue (Figure 3A). However, both subtypes show more or less similar ratios of petiole area to lamina area (Table 2). While the mostly herbaceous *Begonia* grow in the ground layers of the forest, the three Euphorbiaceae species grow into shrubs or trees and thus, the leaves are exposed to higher external forces such as wind and rainfall. Here, the high proportion of strengthening tissue and crosslinking might provide the needed stability. The fact that the species share the same type of transition structure supports the assumption that the structure is typical for Euphorbiaceae, rather than influenced by origin or environmental conditions.

#### 4.2.3. Dense Structures in the Petiole–Lamina Junction—The Ring- and Knot-Like Structures

The peculiar ring-like structure in the petiole–lamina transition (Type C) was observed only in Menispermaceae (*Perichasma laetificata* and *Stephania delavayi*, Figure 2C). Both show high proportions of sclerenchyma tissue in the petiole (*P. laetificata*: 18.77%, *S. delavayi*: 12.14%, Table 2 and Figure 3A). Both species are climbers but are native to different continents [30,40,65]. The complex ring-like structure in the transition zone and the high proportion of sclerenchyma probably is a specific for one family, as in the Euphorbiaceae.

The fibre-dense knot-like structure (Type D) was found in two species from two different orders, *Tropaeolum tuberosum* (Brassicales) and *Hydrocotyle vulgaris* (Apiales) (Figure 4). In both species the fibre strands merge into a dense knot in the transition zone. As a climbing plant, *T. tuberosum* is found in the Andean regions of South America [71]. *Tropaeolum tuberosum* is climbing with leaf-stalk tendrils [71] i.e., the petioles carry most of the plant’s weight. High amounts of strengthening tissue in the petiole (12.8%, Table 2) most probably ensure a secure connection between petiole and support. The anatomical analysis with light microscopy was not able to differentiate between individual fibre strands in the junction. Further analysis of such structures in other peltate species of *Tropaeolum* and *Hydrocotyle*, applying CT- or MRI-scanning, like in Sacher et al. (2019) will shed further light on the fibre orientation in this type of joint [13].

#### 4.2.4. Specifics of Water Plants

Aquatic plants with floating leaves are treated separately here, because of the specific growth under which it is generally not necessary to support the weight of the leaves (except *Nelumbo*, some *Nymphaea* species or *Marsilea*).

The analysis of the transition zone of several peltate leaves of water plants revealed two different types, (1) the radial branching type (Type G, *Nelumbo nucifera, Nymphaea colorata*) and (2) the diffuse branching type (Type H, *Victoria cruziana, V. amazonica, Euryale ferox*). Though plants with floating leaves are not exposed to wind or need the strengthening tissue to raise the lamina above the water, they nonetheless need to withstand other challenges such as resisting water turbulences, flooding, drought or ensuring gas exchange throughout the plant body. All investigated aquatic species show the characteristically large ventilation channels in the petioles (Figure 2G,H), which are slightly different in each species. All species have scattered vascular bundles, except for some larger ones that are located between the ventilation channels. In addition, some layers of collenchyma tissue have been found underneath the epidermis in each species. Compared to other peltate leaved species, *E. ferox* has a remarkably high ratio of petiole to lamina area (being 0.28%, Table 2). A wider petiole could be advantageous for the gas exchange between lamina and the submersed rhizome. In addition, a robust petiole securely connected to the lamina could prevent the lamina from tearing off due to water movement or other mechanical stresses.

The genus *Nelumbo* holds a special position among the analysed aquatic plants with floating leaves. *Nelumbo* produces floating leaves comparable to the other aquatic species but mostly leaves that are raised above the water [38,57]. The strengthening structure in the petiole–lamina junction is similar to the one of *Nymphaea*. The large amount of strengthening tissue in the petiole (19.3%, Table 2), when compared to *N. colorata* with floating leaves (5,7%, Table 2) indicates that higher amounts of strengthening tissue are needed to withstand the higher gravitational forces and external stresses such as wind and rain that are affecting the emergent lamina.

*Cabomba aquatica* could not be assigned to one of the previously described types. *Cabomba* predominantly forms submerged leaves while floating leaves appear only at flowering time [83]. The anatomy of the petiole, including large ventilation channels, is similar to the petioles of other aquatic plants. However, the central position of just two fibre strands is unique. The strands spread into an undefined dense structure in the transition zone and subsequently divide into four strands that finally unevenly branch within the lamina. With this central position of vascular bundles and fibres, further analysis is needed to clarify the detailed structure of the transitions zone of *C. aquatica*.

#### 4.2.5. Conclusions and Outlook

Since the analysis of peltate species from 18 different families revealed seven distinct types of structures in the petiole–lamina junction, it is probable to find more principles of strengthening structures in the remaining 22 families with peltate leaved plant species. Future studies will follow up on this hypothesis.

When comparing water content, amount of strengthening tissue and anatomical observations for the seven different principles, especially the dense structures (knot-like, ring-like), structures with intense branching (net-like, intensity 2) and with large numbers of vascular strands (simple branching) are likely to show a higher stiffness and rigidity than those structures with less strengthening tissue and mostly depending on turgor pressure. Biomechanical analyses of the different types will clarify this theoretical assumption and are already in progress.

Although the anatomical analysis, applying light microscopy, provided good results to obtain an insight into the different structures in the petiole–lamina transition zone, other methods are needed to allow for a more detailed analysis of the orientation, branching, and merging of the fibre strands. These investigations, i.e., CT (computed tomography)- and MRI (magnetic resonance imaging)-scanning are currently under way and will be combined with the biomechanical data of the different structural elements, eventually resulting in models that will be used for bioinspired structures in lightweight constructions such as carbon-concrete components.

## Figures and Tables

**Table 1:** List of selected peltate species among vascular plants.

Clade	Order	Family	Species	Continent	Distribution	Habit		Habitat	Floral Zone	Floristic Kingdom	Peltation
F	Salviniales	Marsileaceae	*Marsilea ancylopoda* A. Braun	North America, South America	Tropical America, Subtropical America	h	p	a	boreostrop, trop, austrostrop, austr	Neo	c
F	Salviniales	Marsileaceae	*Marsilea batardae* Launert	Europe	Portugal, Spain	h	p	a	sm, m	Hol	c
F	Salviniales	Marsileaceae	*Marsilea macrocarpa* C. Presl	Africa	C Africa, S Africa, Madagascar	h	p	a	trop, austrostrop	Pal	c
F	Salviniales	Marsileaceae	*Marsilea oligospora* Goodd.	North America	USA	h	p	a	t, sm, m	Hol	c
F	Salviniales	Marsileaceae	*Marsilea strigosa* Willd.	Europe	Mediterranean region, Russia, Kazakhstan	h	-	a	sm	Hol	c
ANA	Nymphaeales	Cabombaceae	*Brasenia schreberi* J.F.Gmel.	Africa, South America, Asia	worldwide	h	p	a	austr, strop, trop, m, sm, temp	Hol, Pal, Neo, Aus	e
ANA	Nymphaeales	Cabombaceae	*Cabomba aquatica* Aubl.	South America	S America	h	p	a	strop, trop	Neo	c
ANA	Nymphaeales	Nymphaeaceae	*Euryale ferox* Salisb. ex K.D.Koenig and Sims	Asia	Asia	h	a,p	a	boreostrop, m	Pal	c
ANA	Nymphaeales	Nymphaeaceae	*Nymphaea colorata* Peter	Africa	Tanzania	h	p	a	trop	Pal	e
ANA	Nymphaeales	Nymphaeaceae	*Nymphaea gigantea* Hook.	Australia	Australia	h	p	a	austrostrop	Aus	e
ANA	Nymphaeales	Nymphaeaceae	*Nymphaea lotus* L.	Africa	Africa	h	p	a	strop, trop	Pal	e
ANA	Nymphaeales	Nymphaeaceae	*Victoria amazonica* (Poepp.) J.C. Sowerby	South America	S America	h	p	a	trop	Neo	c
ANA	Nymphaeales	Nymphaeaceae	*Victoria cruziana* Orb.	South America	S America	h	p	a	austrostrop	Neo	c
MAG	Laurales	Hernandiaceae	*Hernandia nymphaeifolia* (C.Presl) Kubitzki	Asia, Africa	SE Asia, Madagascar	l	p	t	trop	Pal	e
MAG	Laurales	Hernandiaceae	*Hernandia sonora L.*	North America	Mexico, Caribbean	l	p	t	boreostrop	Neo	e
MAG	Piperales	Piperaceae	*Peperomia argyreia* E.Morr.	South America	Brazil	h	p	t	austrostrop	Neo	e
MAG	Piperales	Piperaceae	*Peperomia bracteata* A.W.Hill	North America	Guatemala, Mexico	h	p	t	boreostrop	Neo	c
MAG	Piperales	Piperaceae	*Peperomia cyclaminoides* A.W. Hill	South America	Bolivia	h	-	t	boreostrop	Neo	c
MAG	Piperales	Piperaceae	*Peperomia monticola* Miq.	North America	Mexico	h	p	t	boreostrop	Neo	e
MAG	Piperales	Piperaceae	*Peperomia sodiroi* C.DC.	South America	Ecuador	h	p	t	trop	Neo	e
MAG	Piperales	Piperaceae	*Piper peltatum* L.	North America, South America	S America, C America	h,l	p	t	strop, trop	Neo	e
MAG	Piperales	Piperaceae	*Piper fragile* Benth.	Asia	C Malesia, W Pacific	-	-	t	trop	Pal, Aus	e
MAG	Piperales	Piperaceae	*Piper peltatifolium* C.Y. Hao, H.S. Wu, Y.H. Tan	Asia	China	l	p	t	boreostrop	Pal	e
MON	Alismatales	Araceae	*Alocasia cuprea* K.Koch	Asia	Borneo	h	p	t	trop	Pal	e
MON	Alismatales	Araceae	*Alocasia fallax* Schott	Asia	Bangladesh, India	h	p	t	boreostrop	Pal	e
MON	Alismatales	Araceae	*Alocasia longiloba* Miq.	Asia	SE Asia	h	p	t	boreostrop, trop	Pal	e
MON	Alismatales	Araceae	*Alocasia peltata* M. Hotta	Asia	Borneo	h	p	t	trop	Pal	e
MON	Alismatales	Araceae	*Alocasia reversa* N.E. Br.	Asia	Borneo	h	p	t	trop	Pal	e
MON	Alismatales	Araceae	*Amorphophallus konjac* K.Koch	Asia	China, SE Asia	h	p	t	m	Hol	e
MON	Alismatales	Araceae	*Anthurium forgetii* N.E.Br.	South America	Colombia	h	p	t	trop	Neo	e
MON	Alismatales	Araceae	*Anthurium jureianum* Cath. and Olaio	South America	Brazil	h	p	t	austrostrop	Neo	e
MON	Alismatales	Araceae	*Ariopsis peltata* Nimmo	Asia	India	h	p	t	boreostrop, trop	Pal	e
MON	Alismatales	Araceae	*Ariopsis proanthera* N.E. Br.	Asia	Bangladesh, India, Myanmar, Nepal, Thailand	h	p	t	boreostrop, trop	Pal	e
MON	Alismatales	Araceae	*Arisaema caudatum* Engl.	Asia	India	h	p	t	boreostrop, trop	Pal	c
MON	Alismatales	Araceae	*Arisaema ciliatum* H.Li	Asia	China	h	p	t	m	Hol	c
MON	Alismatales	Araceae	*Arisaema fischeri* Manudev and Nampy	Asia	India	h	p	t	boreostrop, trop	Pal	c
MON	Alismatales	Araceae	*Arisaema peltatum* C.E.C. Fisch.	Asia	India	h	p	t	boreostrop, trop	Pal	e
MON	Alismatales	Araceae	*Arisaema subulatum* Manudev and Nampy	Asia	India	h	p	t	boreostrop, trop	Pal	c
MON	Alismatales	Araceae	*Caladium bicolor* Vent.	North America, South America	C America, S America	h	p	t	boreostrop, trop	Neo	e
MON	Alismatales	Araceae	*Caladium clavatum* Hett., Bogner and J. Boos	South America	Ecuador	h	p	t	trop	Neo	e
MON	Alismatales	Araceae	*Caladium humboldtii* (Raf.) Schott	South America	Brazil, Venezuela	h	p	t	trop	Neo	e
MON	Alismatales	Araceae	*Caladium smaragdinum* K. Koch and C.D. Bouché	South America	Colombia, Venezuela	h	p	t	trop	Neo	e
MON	Alismatales	Araceae	*Caladium steudnerifolium* Engl.	South America	Bolivia, Colombia, Ecuador, Peru	h	p	t	trop, austrostrop	Neo	e
MON	Alismatales	Araceae	*Colocasia boyceana* Gogoi and Borah	Asia	India	h	p	t	boreostrop, trop	Pal	e
MON	Alismatales	Araceae	*Colocasia esculenta* (L.) Schott	Asia	SE Asia	h	p	t	trop, boreostrop, m	Pal	e
MON	Alismatales	Araceae	*Colocasia fallax* Schott	Asia	SE Asia	h	p	t	m, boreostrop	Pal	e
MON	Alismatales	Araceae	*Colocasia hassanii* H. Ara	Asia	Bangladesh	h	p	t	boreostrop	Pal	e
MON	Alismatales	Araceae	*Colocasia mannii* Hook. f.	Asia	Bangladesh, India	h	p	t	boreostrop	Pal	e
MON	Alismatales	Araceae	*Remusatia hookeriana* Schott	Asia	China, India, Myanmar, Nepal, Thailand	h	p	t	m, boreostrop, trop	Hol, Pal	e
MON	Alismatales	Araceae	*Remusatia pumila* (D.Don) H.Li and A.Hay	Asia	China, Bangladesh, Nepal, Thailand	h	p	t	boreostrop	Pal	e
MON	Alismatales	Araceae	*Remusatia vivipara* (Roxb.) Schott	Africa, Asia	Africa, Australia, SE Asia	h	p	t	strop, trop	Pal, Aus	e
MON	Alismatales	Araceae	*Remusatia yunnanensis* (H. Li and A. Hay) A. Hay	Asia	China, Taiwan	h	p	t	m, boreostrop	Hol, Pal	e
MON	Alismatales	Araceae	*Steudnera assamica* Hook. f.	Asia	India	h	p	t	boreostrop	Pal	e
MON	Alismatales	Araceae	*Steudnera colocasiifolia* K.Koch	Asia	SE Asia, China	h	p	t	m, boreostrop	Hol, Pal	e
MON	Alismatales	Araceae	*Steudnera discolor* W. Bull	Asia	India, Bangladesh, Myanmar, Thailand	h	p	t	boreostrop	Pal	e
MON	Alismatales	Araceae	*Steudnera kerrii* Gagnep.	Asia	China, Laos, Thailand, Vietnam	h	p	t	boreostrop	Pal	e
MON	Alismatales	Araceae	*Steudnera virosa* (Roxb.) Prain	Asia	Bangladesh, India	h	p	t	boreostrop, trop	Pal	e
MON	Alismatales	Araceae	*Xanthosoma peltatum* G.S. Bunting	South America	Venezuela	h	p	t	trop	Neo	e
EUD	Proteales	Nelumbonaceae	*Nelumbo lutea* Willd.	North America	S USA	h	p	a	boreostrop, m	Hol	c
EUD	Proteales	Nelumbonaceae	*Nelumbo nucifera* Gaertn.	Asia	India	h	p	a	boreostrop	Pal	c
EUD	Ranunculales	Berberidaceae	*Podophyllum delavayi* Franch.	Asia	SC China	h	p	t	m	Hol	e
EUD	Ranunculales	Berberidaceae	*Podophyllum glaucescens* J.M.H.Shaw	Asia	SE China	h	p	t	m	Hol	e
EUD	Ranunculales	Berberidaceae	*Podophyllum peltatum* L.	North America	N America	h	p	t	m, sm, temp, b	Hol	c
EUD	Ranunculales	Berberidaceae	*Podophyllum pleianthum* Hance	Asia	SE China, Taiwan	h	p	t	m	Hol	c
EUD	Ranunculales	Berberidaceae	*Podophyllum versipelle* Hance	Asia	China, Vietnam	h	p	t	boreostrop	Pal	c
EUD	Ranunculales	Menispermaceae	*Cissampelos glaberrima* A. St.-Hil.	South America	Tropical South America	h,l	p	t	trop, austrostrop	Neo	e
EUD	Ranunculales	Menispermaceae	*Cissampelos grandifolia* Triana and Planch.	North America, South America	C America, Tropical S America	h,l	p	t	boreostrop, trop, austrostrop	Neo	e
EUD	Ranunculales	Menispermaceae	*Cissampelos hispida* Forman	Asia	Thailand	l	p	t	boreostrop, trop	Pal	e
EUD	Ranunculales	Menispermaceae	*Cissampelos owariensis* P. Beauv. ex DC.	Africa	Tropical Africa	h,l	p	t	boreostrop, trop	Pal	e
EUD	Ranunculales	Menispermaceae	*Cissampelos sympodialis* Eichler	South America	Brazil	h,l	p	t	trop, austrostrop	Neo	e
EUD	Ranunculales	Menispermaceae	*Coscinium blumeanum* Miers ex Hook.f. and Thomson	Asia	Malaysia, Thailand, Vietnam	l	p	t	boreostrop, trop	Pal	e
EUD	Ranunculales	Menispermaceae	*Cyclea cauliflora* Merr.	Asia	Philippines, Sulawesi	h,l	p	t	boreostrop, trop	Pal	e
EUD	Ranunculales	Menispermaceae	*Cyclea debiliflora* Miers	Asia	China, India, Vietnam	h,l	p	t	m, boreostrop, trop	Pal	e
EUD	Ranunculales	Menispermaceae	*Cyclea fissicalyx* Dunn	Asia	India	h,l	p	t	boreostrop, trop	Pal	e
EUD	Ranunculales	Menispermaceae	*Cyclea hypoglauca* (Schauer) Diels	Asia	China, Vietnam	h,l	p	t	m, boreostrop, trop	Pal	e
EUD	Ranunculales	Menispermaceae	*Cyclea peltata* (Lam.) Hook. f. and Thomson	Asia	SE Asia, India	h,l	p	t	boreostrop	Pal	e
EUD	Ranunculales	Menispermaceae	*Disciphania calocarpa* Standl.	North America	C America	l	p	t	boreostrop	Neo	e
EUD	Ranunculales	Menispermaceae	*Disciphania contraversa* Barneby	South America	Brazil	-	p	t	austrostrop	Neo	e
EUD	Ranunculales	Menispermaceae	*Disciphania hernandia* (Vell.) Barneby	South America	Brazil	h,l	p	t	trop, austrostrop	Neo	e
EUD	Ranunculales	Menispermaceae	*Menispermum dauricum* DC.	Asia	China, Mongolia, Russia, Japan, Korea	h,l	p	t	b, temp, sm, m, boreostrop	Hol, Pal	e
EUD	Ranunculales	Menispermaceae	*Perichasma laetificata* Miers	Africa	WC Africa	-	-	t	trop	Pal	e
EUD	Ranunculales	Menispermaceae	*Stephania abyssinica* (Quart.-Dill. and A.Rich.) Walp.	Africa	Africa	h	p	t	strop, trop	Pal	e
EUD	Ranunculales	Menispermaceae	*Stephania brevipes* Craib	Asia	Thailand, Vietnam	h	p	t	boreostrop, trop	Pal	e
EUD	Ranunculales	Menispermaceae	*Stephania delavayi* Diels	Asia	China, Myanmar	h	p	t	boreostrop	Pal	e
EUD	Ranunculales	Menispermaceae	*Stephania japonica* (Thunb.) Miers	Asia, Australia and Oceania	Tropical Asia, Subtropical Asia, Australia	h	p	t	boreostrop, trop, austrostrop	Pal, Aus	e
EUD	Ranunculales	Menispermaceae	*Stephania venosa* Spreng.	Asia	SE Asia	h	p	t	boreostrop, trop	Pal	e
EUD	Ranunculales	Ranunculaceae	*Asteropyrum cavaleriei* H.Lév. and Vaniot	Asia	China	h	p	t	m	Hol	e
EUD	Ranunculales	Ranunculaceae	*Asteropyrum peltatum* (Franch.) J.R.Drumm. and Hutch.	Asia	China, Myanmar	h	p	t	boreostrop	Pal	e
EUD	Ranunculales	Ranunculaceae	*Peltocalathos baurii* (MacOwan) Tamura	Africa	South Africa, Lesotho, Eswatini	h	p	t	austr	Cap	c
EUD	Ranunculales	Ranunculaceae	*Ranunculus clypeatus* (Ulbr.) Lourteig	South America	Peru	h	p	t	trop	Neo	e
EUD	Ranunculales	Ranunculaceae	*Ranunculus lyallii* Hook.f.	Australia	New Zealand	h	p	t	austr	Ant	c
EUD	Ranunculales	Ranunculaceae	*Thalictrum ichangense* Lecoy. ex Oliv.	Asia	China, Korea, Vietnam	h	p	t	boreostrop, m	Hol, Pal	e
EUD	Ranunculales	Ranunculaceae	*Thalictrum pringlei* S. Watson	North America	Mexico	h	p	t	boreostrop	Neo	e
EUD	Ranunculales	Ranunculaceae	*Thalictrum pseudoichangense* Q.E. Yang and G.H. Zhu	Asia	China	h	p	t	m, boreostrop	Hol, Pal	e
EUD	Ranunculales	Ranunculaceae	*Thalictrum roseanum* B.Boivin	North America	Mexico	h	p	t	boreostrop	Neo	e
EUD-C	Apiales	Apiaceae	*Klotzschia brasiliensis* Cham.	South America	Brazil	h	-	t	austrostrop	Neo	e
EUD-C	Apiales	Apiaceae	*Klotzschia glaziovii* Urb.	South America	Brazil	l	p	t	austrostrop	Neo	e
EUD-C	Apiales	Apiaceae	*Klotzschia rhizophylla* Urb.	South America	Brazil	h	-	t	austrostrop	Neo	e
EUD-C	Apiales	Apiaceae	*Petagnaea gussonei* (Spreng.) Rauschert	Europe	Sicilia	h	p	t	m	Hol	c
EUD-C	Apiales	Araliaceae	*Hydrocotyle bonariensis* Lam.	North America, South America	C America, S America	h	p	t	boreostrop, trop	Neo	c
EUD-C	Apiales	Araliaceae	*Hydrocotyle pusilla* R.Br. ex Rich.	North America, South America	C America, S America	h	p	t	boreostrop, trop	Neo	c
EUD-C	Apiales	Araliaceae	*Hydrocotyle umbellata* L.	North America, South America	C America, S America	h	p	t	boreostrop, trop	Neo	c
EUD-C	Apiales	Araliaceae	*Hydrocotyle vulgaris* L.	Europe	Europe	h	p	a	m, sm, temp, b	Hol	c
EUD-C	Apiales	Araliaceae	*Hydrocotyle yanghuangensis* (Hieron.) Mathias	South America	Ecuador	h	p	t	trop	Neo	e
EUD-C	Apiales	Araliaceae	*Oplopanax japonicus* Nakai	Asia	Japan	l	p	t	t, sm	Hol	e
EUD-C	Apiales	Araliaceae	*Schefflera actinophylla* (Endl.) Harms	Australia and Oceania	Australia, New Guinea	l	p	t	trop, austrostrop	Pal, Aus	e
EUD-C	Apiales	Araliaceae	*Schefflera arboricola* (Hayata) Merr.	Asia	Taiwan	l	p	t	m	Hol	e
EUD-C	Apiales	Araliaceae	*Schefflera digitata* J.R. Forst. and G. Forst.	Australia and Oceania	New Zealand	l	p	t	austrostrop, austr	Aus	e
EUD-C	Asterales	Asteraceae	*Ligularia nelumbifolia* Hand.-Mazz.	Asia	China	h	p	t	sm, m, boreostrop	Hol, Pal	e
EUD-C	Asterales	Asteraceae	*Prenanthes subpeltata* Stebbins	Africa	Ethiopia, Kenya, Rwanda, Democratic Republic of Congo	h	p	t	boreostrop, trop	Pal	e
EUD-C	Asterales	Asteraceae	*Psacalium laxiflorum* Benth.	North America	Mexico	h	p	t	boreostrop	Neo	c
EUD-C	Asterales	Asteraceae	*Psacalium megaphyllum* Rydb.	North America	Mexico	h	p	t	boreostrop	Neo	e
EUD-C	Asterales	Asteraceae	*Psacalium peltatum* Cass.	North America	Mexico	h	p	t	boreostrop	Neo	c
EUD-C	Asterales	Asteraceae	*Psacalium pinetorum* (Standl. and Steyerm.) Cuatrec.	North America	Guatemala	h	p	t	boreostrop	Neo	e
EUD-C	Asterales	Asteraceae	*Psacalium putlanum* B.L. Turner	North America	Mexico	h	p	t	boreostrop	Neo	e
EUD-C	Asterales	Asteraceae	*Roldana chapalensis* (S. Watson) H. Rob. and Bretell	North America	Mexico	l	p	t	boreostrop	Neo	e
EUD-C	Asterales	Asteraceae	*Roldana heterogama* (Benth.) H.Rob. and Brettell	North America	Costa Rica, Guatemala, Mexico, Panama	h,l	p	t	boreostrop	Neo	e
EUD-C	Asterales	Asteraceae	*Roldana subpeltata* (Sch. Bip.) H. Rob and Brettell	North America	Mexico	h,l	p	t	boreostrop	Neo	e
EUD-C	Asterales	Asteraceae	*Senecio oxyriifolius* DC.	Africa	C Africa, S Africa	h	p	t	austr, austrostrop, trop	Cap, Pal	e
EUD-C	Asterales	Asteraceae	*Syneilesis aconitifolia* (Bunge) Maxim.	Asia	China, Japan, Korea, Russia	h	p	t	t, sm, m, boreostrop	Hol, Pal	c
EUD-C	Asterales	Asteraceae	*Syneilesis palmata* (Thunb.) Maxim.	Asia	Korea, Japan	h	p	t	sm	Hol	c
EUD-C	Brassicales	Caricaceae	*Jacaratia digitata* (Poepp. and Endl.) Solms	South America	Bolivia, Brazil, Ecuador, Venezuela	l	p	t	strop, trop	Neo	e
EUD-C	Brassicales	Caricaceae	*Jacaratia spinosa* (Aubl.) A.DC.	South America	C America, S America	l	p	t	strop, trop	Neo	e
EUD-C	Brassicales	Tropaeolaceae	*Tropaeolum ciliatum* Ruiz and Pav.	South America	Chile	h	p	t	austrostrop	Neo, Ant	e
EUD-C	Brassicales	Tropaeolaceae	*Tropaeolum majus* L.	South America	Peru	h	p	t	austrostrop	Neo	e
EUD-C	Brassicales	Tropaeolaceae	*Tropaeolum minus* L.	South America	Ecuador, Peru	h	a	t	austrostrop, trop	Neo	e
EUD-C	Brassicales	Tropaeolaceae	*Tropaeolum pentaphyllum* Lam.	South America	Argentina, Brazil, Paraguay, Uruguay	h	p	t	austrostrop	Neo	e
EUD-C	Brassicales	Tropaeolaceae	*Tropaeolum tuberosum* Ruiz and Pav.	South America	Bolivia, Colombia, Ecuador, Peru	h	p	t	austrostrop, trop	Neo	e
EUD-C	Caryophyllales	Polygonaceae	*Coccoloba acapulcensis* Standl.	North America	C America	l	p	t	boreostrop	Neo	e
EUD-C	Caryophyllales	Polygonaceae	*Coccoloba tiliacea* Lindau	South America	Argentina, Bolivia	l	p	t	trop, austrostrop, austr	Neo	e
EUD-C	Caryophyllales	Polygonaceae	*Persicaria perfoliata* (L.) Gross	Europe, Asia	Turkey, India, E Asia, SE Asia	h	a	t	b, temp, sm, m, boreostrop, trop	Hol, Pal	e
EUD-C	Cornales	Loasaceae	*Nasa peltata* (Spruce ex Urb. and Gilg) Weigend	South America	Ecuador, Peru	h	-	t	trop	Neo	e
EUD-C	Cornales	Loasaceae	*Nasa peltiphylla* (Weigend) Weigend	South America	Colombia, Ecuador	-	-	t	trop	Neo	e
EUD-C	Cucurbitales	Begoniaceae	*Begonia caroliniifolia* Regel	North America	Mexico, Guatemala, Honduras	h	p	t	boreostrop	Neo	e
EUD-C	Cucurbitales	Begoniaceae	*Begonia goegoensis* N.E.Br.	Asia	Sumatra	h	p	t	trop	Pal	e
EUD-C	Cucurbitales	Begoniaceae	*Begonia kellermannii* C.DC.	South America	S America	h	p	t	trop	Neo	e
EUD-C	Cucurbitales	Begoniaceae	*Begonia nelumbiifolia* Cham. and Schltdl.	South America	S America	h	p	t	austrostrop	Neo	e
EUD-C	Cucurbitales	Begoniaceae	*Begonia sudjanae* C.-A. Jansson	Asia	Sumatra	h	p	t	trop	Pal	e
EUD-C	Ericales	Balsaminaceae	*Impatiens begonioides* Eb. Fisch. and Raheliv.	Africa	Madagascar	h	-	t	trop, austrostrop	Pal	e
EUD-C	Fabales	Fabaceae	*Lupinus angustifolius* L.	Europe, Africa, Asia	Mediterranean area, China	h	a	t	sm, m	Hol, Pal	e
EUD-C	Fabales	Fabaceae	*Lupinus arboreus* Sims	North America	W USA	h,l	p	t	sm, m	Hol	e
EUD-C	Fabales	Fabaceae	*Lupinus digitatus* Forssk.	Africa	N Africa, Senegal	h	a	t	m, boreostrop	Hol, Pal	e
EUD-C	Fabales	Fabaceae	*Lupinus gussoneanus* J. Agardh	Europe	Mediterranean area	h	a	t	sm, m	Hol	e
EUD-C	Fabales	Fabaceae	*Lupinus mutabilis* Sweet	South America	Bolivia, Colombia, Ecuador, Peru, Venezuela	h,l	p	t	trop	Neo	e
EUD-C	Gentianales	Apocynaceae	*Hoya imbricata* Decne.	Asia	Philippines, Sulawesi	h	p	t	boreostrop, trop	Pal	c
EUD-C	Gentianales	Apocynaceae	*Macropharynx abnorma* J.F. Morales, M.E. Endress and Liede	South America	Peru	h,l	p	t	trop	Neo	e
EUD-C	Gentianales	Apocynaceae	*Macropharynx conflictiva* (J.F. Morales) J.F. Morales, M.E. Endress and Liede	South America	Peru	h,l	p	t	trop	Neo	e
EUD-C	Gentianales	Apocynaceae	*Macropharynx gigantea* (Woodson) J.F. Morales, M.E. Endress and Liede	South America	Peru, Bolivia	h,l	p	t	trop, austrostrop	Neo	e
EUD-C	Gentianales	Apocynaceae	*Macropharynx macrocalyx* (Müll. Arg.) J.F. Morales, M.E. Endress and Liede	South America	Brazil, Paraguay	h,l	p	t	trop, austrostrop	Neo	e
EUD-C	Gentianales	Apocynaceae	*Macropharynx peltata* (Vell.) J.F. Morales, M.E. Endress and Liede	South America	Argentina, Brazil, Paraguay	h,l	p	t	trop, austrostrop	Neo	e
EUD-C	Gunnerales	Gunneraceae	*Gunnera antioquensis* L.E. Mora	South America	Colombia	h	p	t	trop	Neo	e
EUD-C	Gunnerales	Gunneraceae	*Gunnera brephogea* Linden and André	South America	Colombia, Ecuador, Peru	h	p	t	trop	Neo	c
EUD-C	Gunnerales	Gunneraceae	*Gunnera peltata* Phil.	South America	Juan Fernández Is.	h	p	t	austr	Ant	c
EUD-C	Gunnerales	Gunneraceae	*Gunnera quitoensis* L.E. Mora	South America	Ecuador	h	p	t	trop	Neo	e
EUD-C	Gunnerales	Gunneraceae	*Gunnera silvioana* L.E. Mora	South America	Colombia, Ecuador	h	p	t	trop	Neo	e
EUD-C	Lamiales	Gesneriaceae	*Cyrtandra toviana* F. Br.	Australia and Oceania	Marquesas Islands	l	p	t	trop	Pal	e
EUD-C	Lamiales	Gesneriaceae	*Cyrtandra wawrae* C.B. Clarke	Australia and Oceania	Hawaii	-	-	t	trop	Pal	e
EUD-C	Lamiales	Gesneriaceae	*Drymonia peltata* (Oliv.) H.E. Moore	North America	Costa Rica	-	-	t	boreostrop	Neo	e
EUD-C	Lamiales	Gesneriaceae	*Metapetrocosmea peltata* (Merr. and Chun) W.T. Wand	Asia	China	h	p	t	boreostrop	Pal	e
EUD-C	Lamiales	Gesneriaceae	*Paraboea peltifolia* D. Fang and L. Zeng	Asia	China	h	p	t	m, boreostrop	Hol, Pal	e
EUD-C	Lamiales	Gesneriaceae	*Paraboea yunfuensis* F. Wen and Y.G. Wie	Asia	China	h	p	t	m, boreostrop	Hol, Pal	e
EUD-C	Lamiales	Gesneriaceae	*Petrocosmea huanjiangensis* Yan Liu and W.B. Xu	Asia	China	h	p	t	m, boreostrop	Hol, Pal	e
EUD-C	Lamiales	Gesneriaceae	*Petrocosmea pubescens* D.J. Middleton and Triboun	Asia	Thailand	h	p	t	boreostrop, trop	Pal	e
EUD-C	Lamiales	Gesneriaceae	*Sinningia tuberosa* (Mart.) H.E. Moore	South America	Brazil	h	p	t	austrostrop	Neo	e
EUD-C	Lamiales	Gesneriaceae	*Streptocarpus mandrerensis* Humbert	Africa	Madagascar	h	-	t	trop, austrostrop	Pal	e
EUD-C	Lamiales	Gesneriaceae	*Streptocarpus peltatus* Randrian., Phillipson, Lowry and Mich. Möller	Africa	Madagascar	h	p	t	trop, austrostrop	Pal	e
EUD-C	Lamiales	Gesneriaceae	*Trichodrymonia peltatifolia* (J.L. Clark and M.M. More) M.M. Mora and J.L. Clark	North America	Panama	h	-	t	boreostrop	Neo	e
EUD-C	Lamiales	Lentibulariaceae	*Utricularia nelumbifolia* Gardn.	South America	Brazil	h	p	a	austrostrop	Neo	c
EUD-C	Lamiales	Lentibulariaceae	*Utricularia pubescens* Sm.	Africa, South America, Asia	S America, Africa, India	h	a	t	strop, trop	Neo, Pal	c
EUD-C	Malpighiales	Euphorbiaceae	*Endospermum moluccanum* (Teijsm. and Binn.) Kurz	Asia, Australia and Oceania	Indonesia, New Guinea, Solomon Islands	l	p	t	trop	Pal	e
EUD-C	Malpighiales	Euphorbiaceae	*Endospermum peltatum* Merr.	Asia	SE Asia	l	p	t	trop	Pal	e
EUD-C	Malpighiales	Euphorbiaceae	*Homalanthus grandifolius* Ridl.	Asia	Borneo, Sumatra	l	p	t	trop	Pal	e
EUD-C	Malpighiales	Euphorbiaceae	*Homalanthus macradenius* Pax and K. Hoffm.	Asia	Philippines	l	p	t	boreostrop, trop	Pal	e
EUD-C	Malpighiales	Euphorbiaceae	*Jatropha hernandiifolia* Vent.	North America	Dominican Republic, Haiti, Puerto Rico	l	p	t	boreostrop	Neo	e
EUD-C	Malpighiales	Euphorbiaceae	*Jatropha nudicaulis* Benth.	South America	Colombia, Ecuador	l	p	t	trop	Neo	e
EUD-C	Malpighiales	Euphorbiaceae	*Jatropha peltata* Sessé	North America	Mexico	h,l	p	t	boreostrop	Neo	e
EUD-C	Malpighiales	Euphorbiaceae	*Jatropha podagrica* Hook.	North America	C America	h,l	p	t	trop	Neo	e
EUD-C	Malpighiales	Euphorbiaceae	*Jatropha weberbaueri* Pax and K. Hoffm.	South America	Peru	-		t	trop	Neo	e
EUD-C	Malpighiales	Euphorbiaceae	*Macaranga bancana* (Miq.) Müll.Arg.	Asia	Thailand, Malesia	l	p	t	boreostrop, trop	Pal	e
EUD-C	Malpighiales	Euphorbiaceae	*Macaranga cuspidata Boivin ex Baill.*	Africa	Madagascar	l	p	t	trop, austrostrop	Pal	e
EUD-C	Malpighiales	Euphorbiaceae	*Macaranga magna* Turrill	Australia and Oceania	Fiji	l	p	t	austrostrop	Aus	e
EUD-C	Malpighiales	Euphorbiaceae	*Macaranga tanarius* (L.) Mull.Arg.	Asia, Australia and Oceania	SE Asia, Australia	l	p	t	strop, trop	Pal, Aus	e
EUD-C	Malpighiales	Euphorbiaceae	*Macaranga thompsonii* Merr.	Australia and Oceania	Mariana Islands	l	p	t	trop	Pal	e
EUD-C	Malpighiales	Euphorbiaceae	*Mallotus floribundus* (Blume) Müll. Arg.	Asia	SE Asia	l	p	t	boreostrop, trop	Pal	e
EUD-C	Malpighiales	Euphorbiaceae	*Mallotus lackeyi* Elmer	Asia	Borneo, Philippines	l	p	t	trop	Pal	e
EUD-C	Malpighiales	Euphorbiaceae	*Mallotus peltatus* (Geiseler) Müll. Arg.	Asia	China, India, SE Asia	l	p	t	m, boreostrop, trop	Pal	e
EUD-C	Malpighiales	Euphorbiaceae	*Mallotus surculosus* P.I. Forst.	Australia and Oceania	Australia	l	p	t	austrostrop	Aus	e
EUD-C	Malpighiales	Euphorbiaceae	*Mallotus thorelii* Gagnep.	Asia	Cambodia, China, Laos, Thailand, Vietnam	l	p	t	m, boreostrop, trop	Pal	e
EUD-C	Malpighiales	Euphorbiaceae	*Manihot fabianae* M. Mend.	South America	Bolivia	l	p	t	trop, austrostrop	Neo	e
EUD-C	Malpighiales	Euphorbiaceae	*Manihot mirabilis* Pax	South America	Paraguay	l	p	t	trop, austrostrop	Neo	e
EUD-C	Malpighiales	Euphorbiaceae	*Manihot peltata* Pohl	South America	Brazil	l	p	t	trop, austrostrop	Neo	e
EUD-C	Malpighiales	Euphorbiaceae	*Megistostigma peltatum* (J.J. Sm.) Croizat	Asia	Java, Sumatra	l	p	t	trop	Pal	e
EUD-C	Malpighiales	Euphorbiaceae	*Meineckia peltata* (Hutch.) G.L. Webster	Africa	Madagascar	l	p	t	trop, austrostrop	Pal	e
EUD-C	Malpighiales	Euphorbiaceae	*Ricinus communis* L.	Africa	Eritrea, Ethiopia, Somalia	h,l	a,p	t	boreostrop	Pal	e
EUD-C	Malpighiales	Passifloraceae	*Adenia penangiana* (Wall. ex G. Don) W.J. de Wilde	Asia	SE Asia	h	p	t	boreostrop, trop	Pal	e
EUD-C	Malpighiales	Passifloraceae	*Passiflora coriacea* Juss.	South America	C America, S America	h	p	t	boreostrop, trop	Neo	e
EUD-C	Malpighiales	Passifloraceae	*Passiflora guatemalensis* S. Watson	North America, South America	C America, Colombia, Venezuela	h	p	t	boreostrop, trop	Neo	e
EUD-C	Malpighiales	Passifloraceae	*Passiflora rubrotincta* Killip	South America	Bolivia	h	p	t	trop, austrostrop	Neo	e
EUD-C	Malpighiales	Passifloraceae	*Passiflora spectabilis* Killip	South America	Peru	h	p	t	trop	Neo	e
EUD-C	Malpighiales	Phyllanthaceae	*Astrocasia peltata* Standl.	North America	Costa Rica, Mexico	l	p	t	boreostrop	Neo	e
EUD-C	Malpighiales	Salicaceae	*Xylosma peltata* (Sleumer) Lescot	Australia and Oceania	New Caledonia	l	p	t	austrostrop	Aus	e
EUD-C	Malvales	Dipterocarpaceae	*Shorea peltata* Symington	Asia	Borneo, Malaysia, Sumatra	l	p	t	trop	Pal	e
EUD-C	Malvales	Malvaceae	*Brownlowia ferruginea* Kosterm.	Asia	Borneo	l	p	t	trop	Pal	e
EUD-C	Malvales	Malvaceae	*Brownlowia havilandii* Stapf	Asia	Borneo	l	p	t	trop	Pal	e
EUD-C	Malvales	Malvaceae	*Brownlowia helferiana* Pierre	Asia	Malaysia, Myanmar, Thailand	l	p	t	boreostrop, trop	Pal	e
EUD-C	Malvales	Malvaceae	*Brownlowia peltata* Benth.	Asia	Borneo, Myanmar, Thailand, Vietnam	l	p	t	boreostrop, trop	Pal	e
EUD-C	Malvales	Malvaceae	*Brownlowia stipulata* Kosterm.	Asia	Borneo	l	p	t	trop	Pal	e
EUD-C	Malvales	Malvaceae	*Pterospermum acerifolium (L.) Willd.*	Asia	India, SE Asia	h	p	t	boreostrop, trop	Pal	e
EUD-C	Myrtales	Melastomataceae	*Catanthera peltata* M.P. Nayar	Asia	Borneo	-		t	trop	Pal	e
EUD-C	Myrtales	Melastomataceae	*Conostegia peltata* (Almeda) Kriebel	North America	Panama	l	p	t	trop	Neo	e
EUD-C	Myrtales	Melastomataceae	*Gravesia peltata* H. Perrier	Africa	Madagascar	h	p	t	trop, austrostrop	Pal	e
EUD-C	Myrtales	Melastomataceae	*Leandra peltata* Wurdack	South America	Peru	l	p	t	trop	Neo	e
EUD-C	Myrtales	Melastomataceae	*Phyllagathis beccariana* (Cogn.) M.P. Nayar	Asia	Borneo	h	p	t	trop	Pal	e
EUD-C	Myrtales	Melastomataceae	*Phyllagathis peltata* Stapf ex Ridl.	Asia	Borneo	h	p	t	trop	Pal	e
EUD-C	Oxalidales	Oxalidaceae	*Oxalis articulata* Savigny	South America	Argentina, Brazil, Uruguay	h	p	t	austrostrop	Neo	c
EUD-C	Oxalidales	Oxalidaceae	*Oxalis bowiei* W.T.Aiton ex G.Don	Africa	South Africa	h	p	t	austr	Cap	c
EUD-C	Oxalidales	Oxalidaceae	*Oxalis decaphylla* Kunth	North America	Mexico	h	p	t	boreostrop	Neo	c
EUD-C	Oxalidales	Oxalidaceae	*Oxalis leucolepis* Diels	Asia	China, Nepal	h	p	t	boreostrop, m	Pal	c
EUD-C	Oxalidales	Oxalidaceae	*Oxalis triangularis* A.St.-Hil.	South America	South America	h	p	t	austrostrop, trop	Neo	c
EUD-C	Rosales	Moraceae	*Dorstenia belizensis* C.C. Berg	North America	Belize	h	p	t	boreostrop	Neo	e
EUD-C	Rosales	Moraceae	*Dorstenia erythrandra* C. Wright ex Griseb.	North America	Cuba, Dominican Republic, Haiti	h	p	t	boreostrop	Neo	e
EUD-C	Rosales	Moraceae	*Dorstenia jamaicensis* Britton	North America	Jamaica	h	p	t	boreostrop	Neo	e
EUD-C	Rosales	Moraceae	*Dorstenia nummularia* Urb. and Ekman	North America	Cuba	h	p	t	boreostrop	Neo	e
EUD-C	Rosales	Moraceae	*Dorstenia peltata* Spreng.	North America	Cuba, Dominican Republic	h	p	t	boreostrop	Neo	e
EUD-C	Rosales	Urticaceae	*Cecropia albicans* Trécil	South America	Peru	l	p	t	trop	Neo	e
EUD-C	Rosales	Urticaceae	*Cecropia distachya* Huber	South America	N South America	l	p	t	trop, austrostrop	Neo	e
EUD-C	Rosales	Urticaceae	*Cecropia elongata* Rusby	South America	Bolivia	l	p	t	trop, austrostrop	Neo	e
EUD-C	Rosales	Urticaceae	*Cecropia latiloba* Miq.	South America	N South America	l	p	t	trop, austrostrop	Neo	e
EUD-C	Rosales	Urticaceae	*Cecropia peltata* L.	South America	C America	l	p	t	boreostrop, trop	Neo	e
EUD-C	Rosales	Urticaceae	*Dendrocnide moroides* (Wedd.) Chew	Asia, Australia and Oceania	Australia, Lesser Sunda Islands, Vanuatu	l	p	t	trop, austrostrop, austr	Pal, Aus	e
EUD-C	Rosales	Urticaceae	*Dendrocnide peltata* (Blume) Miq.	Asia, Australia and Oceania	New Guinea, Malesia	l	p	t	trop	Pal	e
EUD-C	Rosales	Urticaceae	*Elatostema muluense* Rodda and A.K. Monro	Asia	Borneo	h	p	t	trop	Pal	e
EUD-C	Rosales	Urticaceae	*Elatostema peltifolium* (Ridl.) H.J.P. Winkl.	Australia and Oceania	New Guinea	h	-	t	trop	Pal	e
EUD-C	Rosales	Urticaceae	*Musanga cecropioides* R. Br. ex Tedlie	Africa	W and C Africa	l	p	t	trop	Pal	c
EUD-C	Rosales	Urticaceae	*Musanga leo-errerae* Hauman and J. Léonard	Africa	C Africa	l	p	t	trop	Pal	c
EUD-C	Rosales	Urticaceae	*Pilea nonggangensis* Y.G. Wei, L.F. Fu and A.K. Monro	Asia	China	h	p	t	m, boreostrop	Hol, Pal	e
EUD-C	Rosales	Urticaceae	*Pilea panzhihuaensis* C.J. Chen, A.K. Monro and L. Chen	Asia	China	h	p	t	m, boreostrop	Hol, Pal	e
EUD-C	Rosales	Urticaceae	*Pilea peltata* Hance	Asia	China, Vietnam	h	p	t	m, boreostrop, trop	Hol, Pal	e
EUD-C	Rosales	Urticaceae	*Pilea peperomioides* Diels	Asia	China	h	p	t	m	Hol	e
EUD-C	Rosales	Rosaceae	*Rubus peltatus* Maxim.	Asia	China, Japan	h	p	t	sm	Hol	e
EUD-C	Saxifragales	Crassulaceae	*Kalanchoe beharensis* Drake	Africa	Madagascar	h	p	t	trop, austrostrop	Pal	e
EUD-C	Saxifragales	Crassulaceae	*Kalanchoe nyikae* Engl.	Africa	Kenya, Tanzania	h	p	t	trop	Pal	e
EUD-C	Saxifragales	Crassulaceae	*Umbilicus botryoides* Hochst. ex A.Rich.	Africa	Africa	h	p	t	boreostrop, trop	Pal	c
EUD-C	Saxifragales	Crassulaceae	*Umbilicus horizontalis* DC.	Africa, Europe	S Europe, N Africa	h	p	t	m	Hol	c
EUD-C	Saxifragales	Crassulaceae	*Umbilicus luteus* Webb and Berthel.	Europe	Mediterranean region	h	p	t	m, sm	Hol	c
EUD-C	Saxifragales	Crassulaceae	*Umbilicus rupestris* (Salisb.) Dandy	Africa, Europe	Europe, Africa	h	p	t	m, sm	Hol	c
EUD-C	Saxifragales	Saxifragaceae	*Astilboides tabularis* Engl.	Asia	China	h	p	t	sm, temp	Hol	c
EUD-C	Saxifragales	Saxifragaceae	*Chrysosplenium peltatum* Turcz.	Asia	Mongolia, Russia	h	p	t	t, sm, m	Hol	e
EUD-C	Saxifragales	Saxifragaceae	*Darmera peltata* (Torr.) Voss	North America	USA	h	p	t	m	Hol	c
EUD-C	Saxifragales	Saxifragaceae	*Peltoboykinia tellimoides* (Maxim.) H. Hara	Asia	China, Japan	h	p	t	t, sm, m	Hol, Pal	e
EUD-C	Saxifragales	Saxifragaceae	*Peltoboykinia watanabei* (Yatabe) H. Hara	Asia	Japan	h	p	t	t, sm, m	Hol	e
EUD-C	Saxifragales	Saxifragaceae	*Rodgersia aesculifolia* Batalin	Asia	China, Mongolia	h	p	t	sm, m, boreostrop	Hol, Pal	c
EUD-C	Saxifragales	Saxifragaceae	*Rodgersia podophylla* A.Gray	Asia	Japan, Korea	h	p	t	sm	Hol	c
EUD-C	Solanales	Convolvulaceae	*Decalobanthus elmeri* (Merr.) A.R. Simoes and Staples	Asia	Borneo	l	p	t	trop	Neo	e
EUD-C	Solanales	Convolvulaceae	*Decalobanthus peltatus* (L.) A.R. Simoes and Staples	Asia, Australia and Oceania	SE Asia, Australia, Madagascar	l	p	t	trop, austrostrop	Pal, Aus	e
EUD-C	Solanales	Solanaceae	*Nothocestrum peltatum* Skottsb.	Australia and Oceania	Hawaii	l	p	t	trop	Pal	e

The species are sorted by clade and order/family (alphabetically). A maximum of five species per genus are shown (for full list: see Supplementary Materials). Further information shown: distribution, habit, habitat, floristic zone and kingdom, peltation. Abbreviations: in clade: F (ferns), ANA (ANA grade), MAG (magnoliids), MON (monocots), EUD (eudicots), EUD-C (core eudicots), in distribution: N (northern), E (eastern), S (southern), W (western), C (central), in habit: h (herbaceous), l (lignified), a (annual), p (perennial), in habitat: a (aquatic), t (terrestrial), in floristic kingdom: Hol (Holarctic), Pal (Palaeotropics), Neo (Neotropics), Aus (Australis), Cap (Capensis), Ant (Antarctic), circpol (all continents in the floral zone), in floral zone: arct (arctic), b (boreal), temp (temperate), sm (submeridional), m (meridional), boreostrop (boreo-subtropical), strop (subtropical), austrostrop (austro-subtropical), trop (tropical), austr (austral), antarct (antarctic), in peltation: e (eccentric), c (central).

## Data Availability

Data is contained within the article or Supplementary Materials.

## Supplementary Materials

The following are available online at https://www.mdpi.com/article/10.3390/biomimetics6020025/s1, Document S1: Profiles of further studied species, Table S1: List of peltate species among vascular plants. Figure S1: Leaf morphology and anatomy of *Cabomba aquatica* Aubl., Figure S2: Leaf morphology and anatomy of *Nymphaea colorata* Peter, Figure S3: Leaf morphology and anatomy of *Victoria amazonica* (Poepp.) J.C. Sowerby, Figure S4: Leaf morphology and anatomy of *Peperomia argyreia* (Miq.) E. Morren, Figure S5: Leaf morphology and anatomy of *Caladium* hybrid, Figure S6: Leaf morphology and anatomy of *Remusatia vivipara* (Roxb.) Schott, Figure S7: Leaf morphology and anatomy of *Colocasia esculenta* (L.) Schott, Figure S8: Leaf morphology and anatomy of *Jatropha podagrica* Hook., Figure S9: Leaf morphology and anatomy of *Stephania venosa* (Blume) Spreng.
