# Supplementary material for: Strengthening Structures in the Petiole–Lamina Junction of Peltate Leaves
Source: Biomimetics (Basel). 2021 Apr 2;6(2):25. doi: 10.3390/biomimetics6020025 (PMC8167582; doi:10.3390/biomimetics6020025)
Supplement: Supplementary file 1 [file biomimetics-06-00025-s001.zip › Document S1.pdf]

**Additional species profiles:**

***Cabomba aquatica*** **Aubl.** (Figure S1) **–** Habit: aquatic plant, completely submersed except for flowers and a few floating leaves; floating leaves peltate, elliptic, 1-2 cm wide, often absent, submersed leaves divided into long narrow segments, 5-7 parts at the end of the petiole, each part several times divided, terminal divisions 0.1-0.4 mm wide, linear, without nerves or markings; flowers yellow, 4.5-10 mm long. Distribution: Guyana to lower Amazon river (Bolivia, Brazil, Colombia, French Guiana, Guyana, Peru, Suriname, Venezuela), introduced into Bangladesh, Malaya. Ecology: alt. 50-1000 m [1,2]. Petiole anatomy: central cylinder with vascular bundles, aerenchyma with large air channels surrounding central cylinder. Anatomy of the transition zone: vascular bundles from central cylinder branch out into the lamina. Additional data: see Table 2.

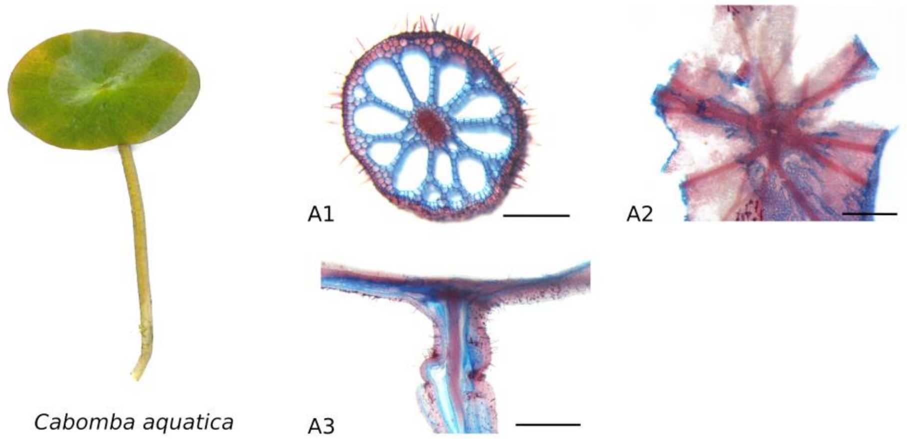


Figure S1: Leaf morphology and anatomy of *Cabomba aquatica* Aubl. A1 – cross section of the petiole. A2 – cross section of the transition zone. A3 – longitudinal section of the transition zone. Scales: 500 µm (A1, A2), 1 mm (A3).

***Nymphaea colorata*** **Peter** (Figure S2) **–** Synonym: *Nymphaea nouchali* var. *zanzibariensis* (Casp.) Verdc. – Habit: more or less robust aquatic herb, perennial; cone-like, tuberous rhizome; leaves rounded to elliptic, 5-45 cm long, 5-40 cm wide, apex rounded, base incised to cordate, narrowly peltate, leaf margin lobulated, lower surface green, purple, red or spotted or dark margin; flowers usually blue, small to very large. Distribution: tropical eastern Africa, southern Africa (Botswana, South Africa, Comoros, Kenya, Madagascar, Mozambique, Namibia, Swaziland, Tanzania, Zambia. Ecology: ponds, lowland streams, alt. 0-1400 m [1,3]. Petiole anatomy: vascular bundles arranged around and in between large air channels (# of vascular bundles: 21), collenchyma below epidermis. Anatomy of the transition zone: fibre bundles run parallel to the air channels into transition zone, forming a symmetric net surrounding the cavities and extend in a radial pattern into the lamina. Additional data: see Table 2.

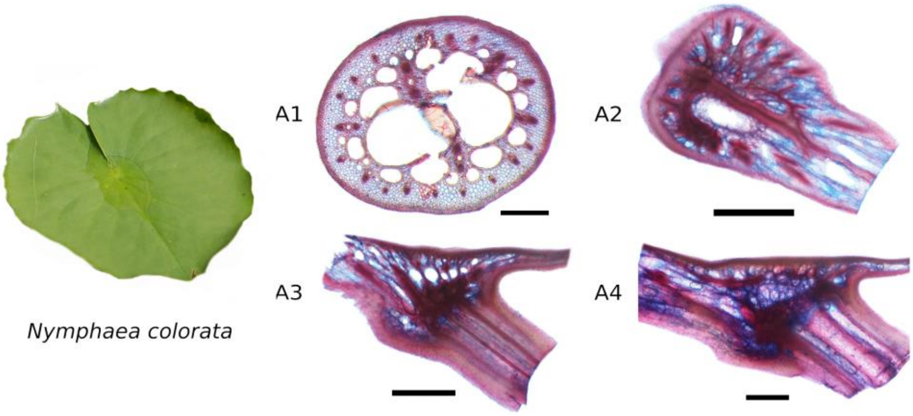


Figure S2: Leaf morphology and anatomy of *Nymphaea colorata* Peter. A1 – cross section of the petiole. A2 – cross section of the transition zone. A3, A4 – longitudinal section of the transition zone. Scales: 1 mm (A1), 2 mm (A2-4).

***Nymphaea lotus* L.** – Leaves were only used for measurements of fresh and dry weight.

***Victoria amazonica* (Poepp.) J.C. Sowerby** (Figure S3) **–** This species is known as the ´Queen of Water Lilies´ and very popular in botanical gardens as an ornamental plant. Habit: perennial water plant; very large peltate floating leaves, can be >2.5 m in diameter; leaves anchored to the base of the plant at the bottom by a long petiole, upper surface green with a waxy cuticula, underside magenta, prickled, with air filled supporting leaf veins, leaf margin bulge 4-6 cm upwards; flowers 25-40 cm wide, relatively short-lived; flower during first opening white, after pollination opens again the next day, appears purplish-red. Distribution: native to tropical South America; Ecology: in swamps, bays and backwaters of rivers in the Amazon basin, the Guianas and the Pantanal [1,4]. Petiole anatomy: large aerenchyma tubes, scattered vascular bundles (# of vascular bundles: 90), layers of collenchyma tissue below epidermis. Anatomy of the transition zone: parallel fibre strands from the petiole branch out in the transition zone forming a diffuse net of fibres in different sizes, fibres running into the lamina follow main veins and become increasingly finer, air channels are visible. Additional data: see Table 2.

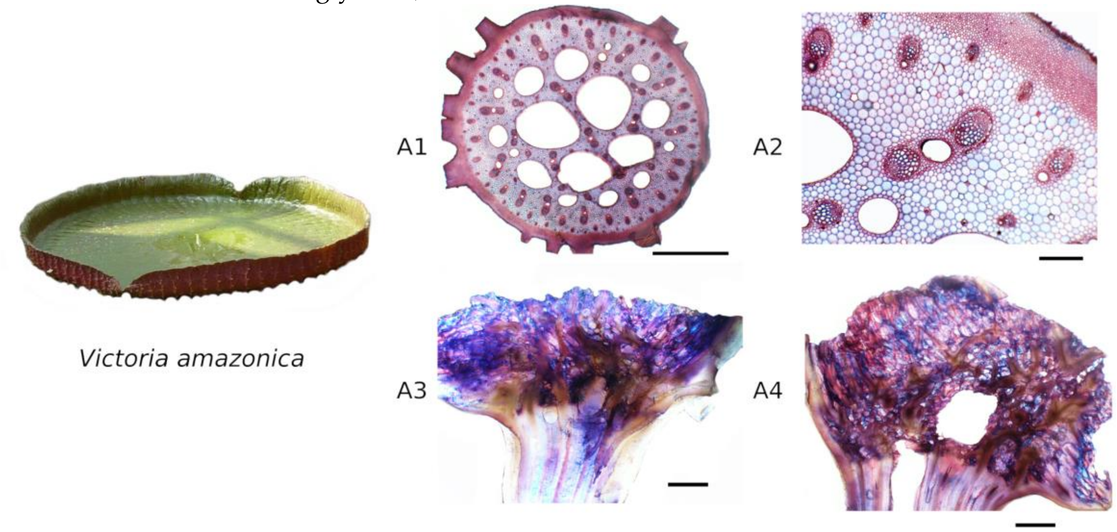


Figure S3: Leaf morphology and anatomy of *Victoria amazonica* (Poepp.) J.C. Sowerby. A1, A2 – cross section of the petiole. A3 – longitudinal section of the transition zone. A4 – cross section of the transition zone. Scales: 500 µm (A2), 5 mm (A1/A3-4).

***Peperomia argyreia* (Miq.) E. Morren** (Figure S4) – Habit: 10 to 20 cm long, red, glabrous petioles; lamina oviform to peltate with 9-11 veins; leaf centre dark green, otherwise alternate green with silver-white stripes, leaf under surface light green, veins rough and protruding. Distribution: Brazil (Bahia, Rio de Janeiro, São Paulo), introduced in Venezuela and Ecuador, cultivated in Colombia at an altitude of 2600 m (Andes) [1]. Petiole anatomy: circular arranged vascular bundles with one central bundle (# of vascular bundles: 8). Anatomy of the transition zone: fibre strands from the petiole spread out into lamina branching out minimally. Additional data: see Table 2.

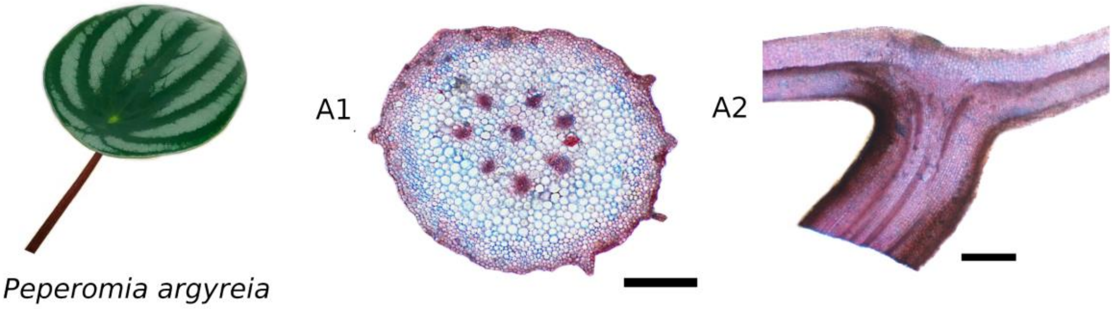


Figure S4: Leaf morphology and anatomy of *Peperomia argyreia* (Miq.) E. Morren. A1 – cross section of the petiole. A2 – longitudinal section of the transition zone. Scales: 500 µm (A1), 1 mm (A2).

***Peperomia cyclaminoides* A.W. Hill** – Petiole anatomy: vascular bundles arranged in a central ring. Anatomy of the transition zone: fibre strands spread out into the lamina branching out minimally. Additional data: see Table 2.

***Alocasia longiloba* Miq.** – Petiole anatomy: scattered vascular bundles and peripheral ring under epidermis. Anatomy of the transition zone: fibre strands from the petiole branch and proceed into leaf veins, some remain unbranched and follow main vein. Additional data: see Table 2.

***Caladium bicolor* (Aiton) Vent.** (Figure S5) **–** This species is widely cultivated as an ornamental plant with many varieties existing (plant from the Botanical Garden of Technische Universität Dresden is a hybrid). Habit: acaulescent herb with a tuber; leaves peltate, 13-37 cm long, 9-26 cm broad, adaxially green with white and red blotches. Distribution: Central America to Argentina, introduced to West Africa, parts of Asia. Ecology: altitude 30-2300 m [1]. Petiole anatomy: scattered vascular bundles and peripheral ring under epidermis (# of vascular bundles: 50), collenchyma strands associated with vascular bundles near the epidermis. Anatomy of the transition zone: fibre strands from the petiole branch and proceed into leaf veins, some remain unbranched and follow main vein. Additional data: see Table 2.

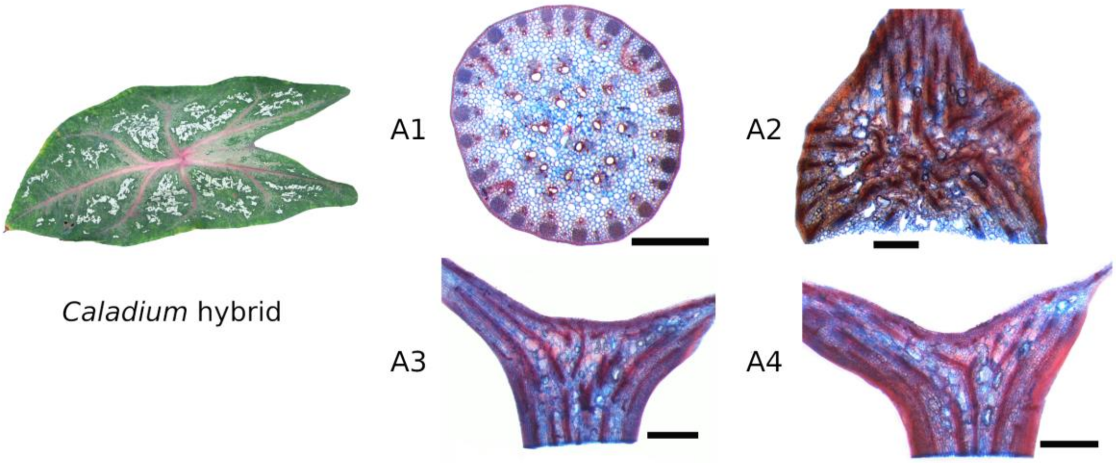


Figure S5: Leaf morphology and anatomy of *Caladium* hybrid. A1 – cross section of the petiole. A2 – cross section of the transition zone. A3, A4 – longitudinal section of the transition zone. Scales: 1 mm (A1-4).

***Remusatia vivipara* (Roxb.) Schott** (Figure S6) – Habit: Seasonally dormant herb, forms flattened globose tuber with 3-5 cm; 1-3 petioles (19-50 cm) grow from tuber with leaves 11-33 cm long, 7-19 cm wide, shiny on both sides, abaxially light green, adaxially green, leaf blade peltate or ovate, insertion of the petiole in the lower 1/3, at the base heart-shaped, at the tip pointed, primary veins and midrib very light, 4-7 primary lateral veins on each side, forming an angle of 45º with the midrib; flowers develop before leaves, flower stems 6-12 cm long with a yellow calyx of 6-9 cm length. Distribution: tropical & subtropical Old World, mostly in Africa, Southeast Asia and northeastern Australia. Ecology: forests, epiphytic, on rocks or cliff ledges, altitude 700-1900 m [1,5]. Petiole anatomy: scattered vascular bundles and peripheral ring under epidermis (# of vascular bundles: 28), collenchyma strands associated with vascular bundles near the epidermis. Anatomy of the transition zone: fibre strands from the petiole branch and proceed into leaf veins, some remain unbranched and follow main vein. Additional data: see Table 2.

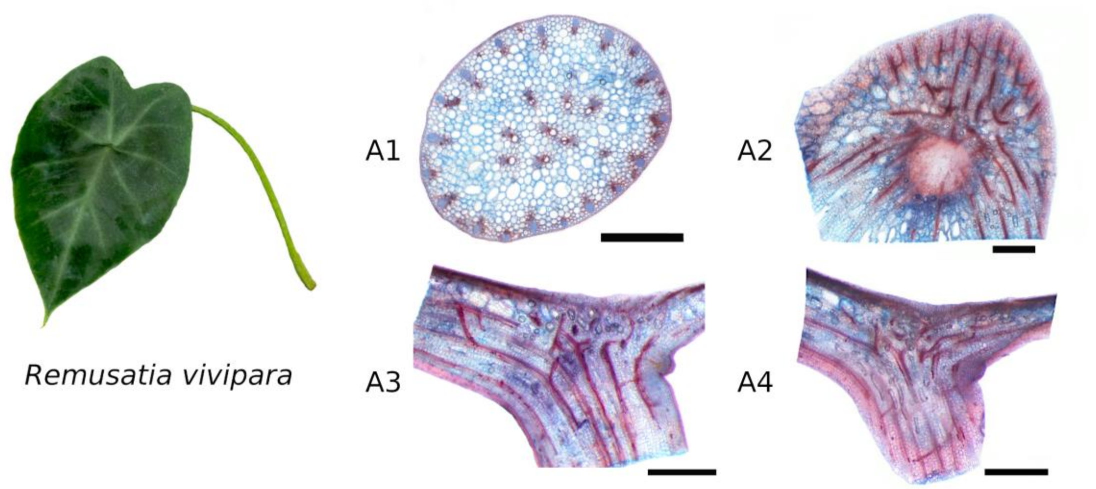


Figure S6: Leaf morphology and anatomy of *Remusatia vivipara* (Roxb.) Schott. A1 – cross section of the petiole. A2 – cross section of the transition zone. A3, A4 – longitudinal section of the transition zone. Scales: 1 µm (A1-4).

***Colocasia esculenta*** **(L.) Schott** (Figure S7) **–** This Species called "Elephant's-ear" or wild taro. The rhizomes, petioles and inflorescences are used as a vegetable; the rhizomes are also used for medical purposes. Habit: strong erect herb, up to 2 m height, forms a tuberous rhizome with 5-15 cm in diameter; two or more green petioles emerge directly from rhizome, leaves peltate, heart-shaped sagittate, margins entire, up to 45 cm long, up to 35 cm wide, basal lobes rounded, tip pointed, lamina greenish to dark green on top, sometimes dark violet adaxially, pale green or glaucous abaxially, 5-7 veins diverging from midrib, adaxially visible and adaxially slightly raised; flowering in late spring and late fall. Distribution: Southeast Asia, introduced in tropical America, Africa and Australia. Ecology: forms colonies along streams, swamps and other wet areas, altitude 0-2200 m [1,5,6]. Petiole anatomy: scattered vascular bundles and peripheral ring under epidermis (# of vascular bundles: 90), collenchymatic tissue associated with vascular bundles near the epidermis. Anatomy of the transition zone: fibre strands from the petiole branch and proceed into leaf veins, some remain unbranched and follow main vein. Additional data: see Table 2.

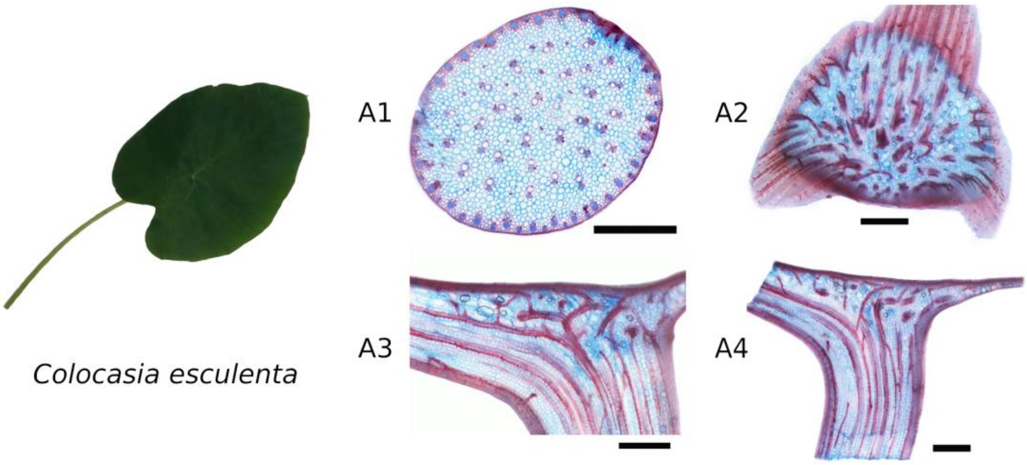


Figure S7: Leaf morphology and anatomy of *Colocasia esculenta* (L.) Schott. A1 – cross section of the petiole. A2 – cross section of the transition zone. A3, A4 – longitudinal section of the transition zone. Scales: 1 mm (A1-4).

***Begonia kellermanii*** **C. DC.** – Petiole anatomy: vascular bundles arranged ± in a ring. Anatomy of the transition zone: fibre strands from petiole branch out in the transition zone forming a net and merge into fibre bundles extending into the leaf veins. Additional data: see Table 2.

***Begonia sudjanae* C.-A. Jansson** – Leaves were only used for measurements of fresh and dry weight.

***Jatropha podagrica* Hook.** (Figure S8)**–** **C**ultivated for medicine and as an ornamental plant. Habit: erect shrub, up to 2 m in height, woody stem, swollen at the base, strong branches; petioles fleshy, glabrous, 8-20 cm long, lamina peltate, roundish to elliptical, 3 to 5 lobes, lobes oval to obovate, median lobe up to 20 cm long, up to 11 cm wide, laterals slightly smaller, leaves glabrous, green, leaf margin entire, 6-8 palmate veins; inflorescences terminal, long pedunculated, flowers orange and red. Distribution: southern Mexico to Nicaragua, introduced in Columbia, Africa and India [1,7]**.** Petiole anatomy: ring of vascular tissue with sclerenchymatic fibres on outer side of vascular bundles (# of vascular bundles: 10), central hollow medullary canal, laticifers present. Anatomy of the transition zone: highly branched network of fibre strands. Additional data: see Table 2.

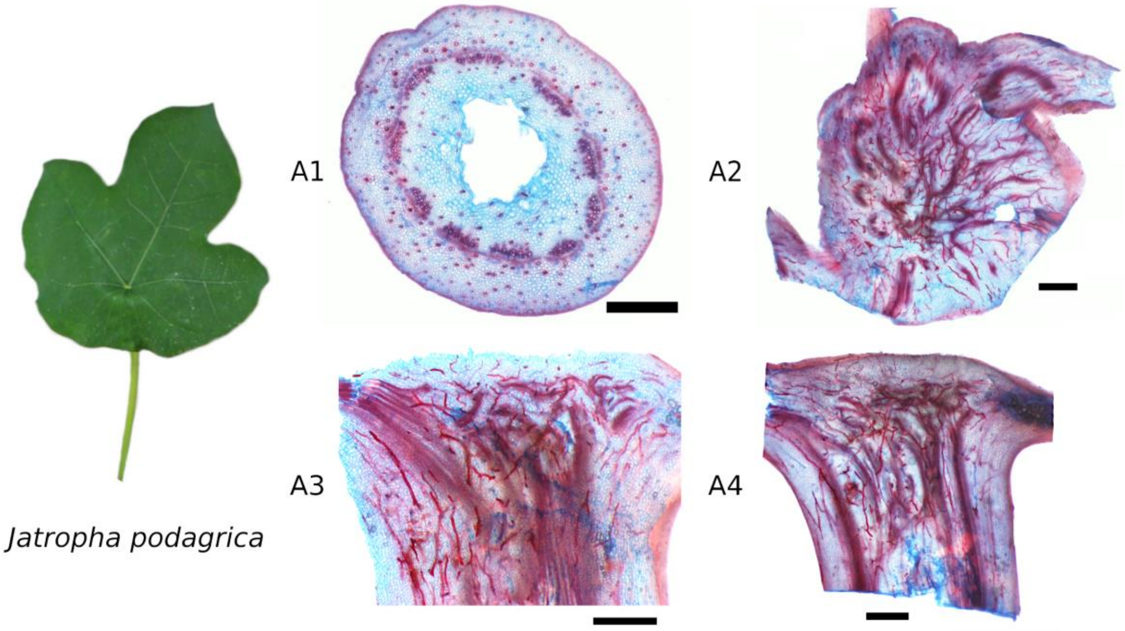


Figure S8: Leaf morphology and anatomy of *Jatropha podagrica* Hook. A1 – cross section of the petiole. A2 – cross section of the transition zone. A3, A4 – longitudinal section of the transition zone. Scales: 1 mm (A1-4).

***Stephania venosa* (Blume) Spreng**. (Figure S9) – Habit: slender climbing plant up to 20 m, stems herbaceous, glabrous; petioles glabrous, 6-22 cm long, lamina triangular ovate, leaf margins slightly lobed, lamina 6-20 cm in length and width, base slightly cordate, abaxially pale with dark venation, glabrous to papillose, adaxially darker, glabrous; inflorescences (male + female) cyme. Distribution: Southeast Asia. Ecology: on hillsides, plains, mountains, forests, up to 1600 m [1,8]. Anatomy of the transition zone: parallel fibre strands from petiole form a closed ring structure in transition zone, from this ring single strands branch into leaf veins.

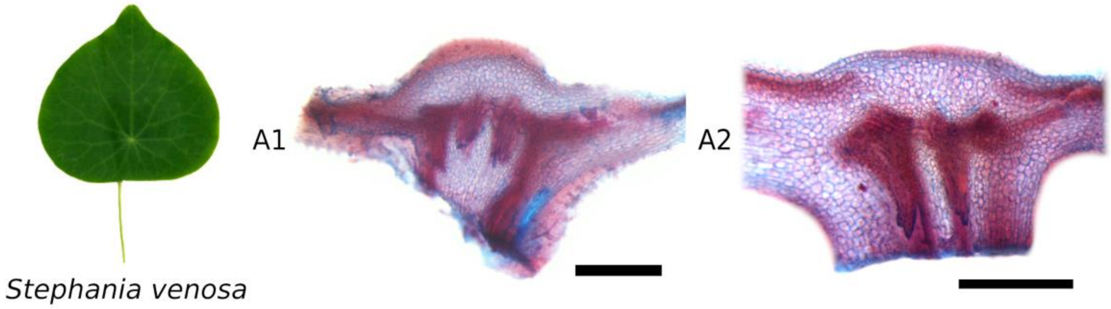


Figure S9: Leaf morphology and anatomy of *Stephania venosa* (Blume) Spreng.. A1, A2 – longitudinal section of the transition zone. Scales: 500 μm (A1-2).

Literature:


1. Royal Botanic Garden, Kew POWO, Plants of the World Online. Available online: http://www.plantsoftheworldonline.org/ (accessed on 29 September 2020).
2. Fassett, N.C. A Monograph of Cabomba. *Castanea* **1953**, *18*, 116–128.
3. Verdcourt, B. Nymphaeaceae. In *Flora of Tropical East Africa*, Polhill, R.M., A. A. Balkema: Rotterdam, **1989**.
4. Bärtels, A. *Tropenpflanzen: Zier- Und Nutzpflanzen*; Ulmer: Stuttgart, **2002**.
5. Wu, Z.; Raven, P.H.; Hong, D. *Flora of China: Acoraceae through Cyperaceae*; Science Press, Missouri Botanical Garden Press: Beijing, St. Louis, **1994**; Vol. 23.
6. Flora of North America Editorial Committee; Morin, N.R. *Flora of North America - Magnoliophyta: Alismatidae, Arecidae, Commelinidae(in Part), and Zingiberidae*; Oxford University Press, **2000**; Vol. 22.
7. Wu, Z.; Raven, P.H.; Hong, D. *Flora of China: Oxalidaceae through Aceraceae*; Science Press [u.a.]: Beijing, **2008**; Vol. 11.
8. van Stenis, C.G.G.J.; van Stenis-Kruseman, M.J. *Flora Malesiana: Series 1 - Spermatophyta, Systematic Revisions*; Martinus Nijhoff Publishers: Dodrecht, Boston, Lancaster, **1986**; Vol. 10, part 2.
