# Supplementary material for: Strengthening Structures in the Petiole–Lamina Junction of Peltate Leaves
Source: Biomimetics (Basel). 2021 Apr 2;6(2):25. doi: 10.3390/biomimetics6020025 (PMC8167582; doi:10.3390/biomimetics6020025)
Supplement: Supplementary file 1 [file biomimetics-06-00025-s001.zip › Table S1.pdf]

Table S1. List of peltate species among vascular plants. The species are sorted by clade and order/family (alphabetically). Further information shown: distribution, habit, habitat, floristic zone and kingdom, peltation. Abbreviations: in Clade: F (ferns), ANA (ANA grade), MAG (magnoliids), MON (monocots), EUD (eudicots), EUD-C (core eudicots), in Distribution: N (Northern), E (Eastern), S (Southern), W (Western), C (Central), in Habit: h (herbaceous), l (lignified), a (annual), p (perennial), in Habitat: a (aquatic), t (terrestrial), in Floristic kingdom: Hol (Holarctic), Pal (Palaeotropics), Neo (Neotropics), Aus (Australis), Cap (Capensis), Ant (Antarctic), circpol (all continents in the floral zone), in Floral zone: arct (arctic), b (boreal), temp (temperate), sm (submeridional), m (meridional), boreostrop (boreo-subtropical), strop (subtropical), austrostrop (austro-subtropical), trop (tropical), austr (austral), antarct (antarctic), in peltation: e (eccentric), c (central).

Clade	Order	Family	Species	Continent	Distribution	Habit		Habitat	Floral zone	Floristic kingdom	Peltation
Fern	Salviniales	Marsileaceae	*Marsilea aegyptiaca* Willd.	Africa, Asia, Europe	N Africa, S Africa, Arabian Peninsula, E Russia, China, India	h	p	a	t, sm, m, boreostrop, austrostrop	Hol, Pal, Cap	c
Fern	Salviniales	Marsileaceae	*Marsilea ancylopoda* A. Braun	North America, South America	Tropical America, Subtropical America	h	p	a	boreostrop, trop, austrostrop, austr	Neo	c
Fern	Salviniales	Marsileaceae	*Marsilea batardae* Launert	Europe	Portugal, Spain	h	p	a	sm, m	Hol	c
Fern	Salviniales	Marsileaceae	*Marsilea macrocarpa* C. Presl	Africa	C Africa, S Africa, Madagascar	h	p	a	trop, austrostrop	Pal	c
Fern	Salviniales	Marsileaceae	*Marsilea oligospora* Goodd.	North America	USA	h	p	a	t, sm, m	Hol	c
Fern	Salviniales	Marsileaceae	*Marsilea strigosa* Willd.	Europe	Mediterranean region, Russia, Kazakhstan	h	p	a	sm	Hol	c
ANA	Nymphaeales	Cabombaceae	*Brasenia schreberi* J.F.Gmel.	Africa, South America, Asia	worldwide	h	p	a	austr, strop, trop, m, sm, temp	Hol, Pal, Neo, Aus	c
ANA	Nymphaeales	Cabombaceae	*Cabomba aquatica* Aubl.	South America	South America	h	p	a	strop, trop	Neo	c
ANA	Nymphaeales	Nymphaeaceae	*Euryale ferox* Salisb. ex K.D.Koenig & Sims	Asia	Asia	h	a, p	a	boreostrop, m	Pal	c
ANA	Nymphaeales	Nymphaeaceae	*Nymphaea colorata* Peter	Africa	Tanzania	h	p	a	trop	Pal	e
ANA	Nymphaeales	Nymphaeaceae	*Nymphaea gigantea* Hook.	Australia and Oceania	Australia	h	p	a	austrostrop	Aus	e
ANA	Nymphaeales	Nymphaeaceae	*Nymphaea lotus* L.	Africa	Africa	h	p	a	strop, trop	Pal	e
ANA	Nymphaeales	Nymphaeaceae	*Victoria amazonica* (Poepp.) J.C. Sowerby	South America	S America	h	p	a	trop	Neo	c
ANA	Nymphaeales	Nymphaeaceae	*Victoria cruziana* Orb.	South America	S America	h	p	a	austrostrop	Neo	c
MAG	Laurales	Hernandiaceae	*Hernandia nymphaeifolia* (C.Presl) Kubitzki	Asia, Africa	SE Asia , Madagascar	l	p	t	trop	Pal	e
MAG	Laurales	Hernandiaceae	*Hernandia sonora* L.	North America	Mexico, Caribbean	l	p	t	boreostrop	Neo	e
MAG	Piperales	Piperaceae	*Peperomia albonervosa* G.Mathieu	South America	SW Mexico	h	p	t	boreostrop	Neo	c
MAG	Piperales	Piperaceae	*Peperomia argyreia* E.Morr.	South America	Brazil	h	p	t	austrostrop	Neo	e
MAG	Piperales	Piperaceae	*Peperomia arifolia* Miq.	South America	Argentina, Bolivia, Brazil, Paraguay	h	-	t	austr, austrostrop, trop	Neo	e
MAG	Piperales	Piperaceae	*Peperomia bracteata* A.W.Hill	South America	Guatemala, Mexico	h	p	t	boreostrop	Neo	c
MAG	Piperales	Piperaceae	*Peperomia claytonioides* Kunth	North America	Mexico, C America	h	p	t	boreostrop	Neo	e
MAG	Piperales	Piperaceae	*Peperomia cotyledon* Benth.	South America	Colombia, Ecuador	h	p	t	trop	Neo	e
MAG	Piperales	Piperaceae	*Peperomia cyclaminoides* A.W. Hill	South America	Bolivia	h	-	t	boreostrop	Neo	c
MAG	Piperales	Piperaceae	*Peperomia fugax* C. DC.	North America	Mexico	h	-	t	boreostrop	Neo	e
MAG	Piperales	Piperaceae	*Peperomia gracillima* S. Watson	North America	Mexico	h	p	t	boreostrop	Neo	c
MAG	Piperales	Piperaceae	*Peperomia hernandiifolia* (Vahl) A. Dietr.	North America, South America	C America, S America	h	p	t	strop, trop	Neo	e
MAG	Piperales	Piperaceae	*Peperomia macrandra* C. DC.	South America	Mexico, Guatemala	h	-	t	boreostrop	Neo	e
MAG	Piperales	Piperaceae	*Peperomia monticola* Miq.	South America	Mexico	h	p	t	boreostrop	Neo	e
MAG	Piperales	Piperaceae	*Peperomia polybotrya* Kunth	South America	S America	h	p	t	trop	Neo	e
MAG	Piperales	Piperaceae	*Peperomia schizandra* Trel.	South America	Mexico	h	p	t	boreostrop	Neo	c
MAG	Piperales	Piperaceae	*Peperomia sodiroi* C.DC.	South America	Ecuador	h	p	t	trop	Neo	e

Clade	Order	Family	Species	Continent	Distribution	Habit		Habitat	Floral zone	Floristic kingdom	Peltation
MAG	Piperales	Piperaceae	*Peperomia tuerckheimii*	North America	C America	h	-	t	boreostrop	Neo	e
MAG	Piperales	Piperaceae	*Piper fragile* Benth.	Asia	C Malesia, W Pacific	-	-	t	trop	Pal, Aus	e
MAG	Piperales	Piperaceae	*Piper peltatifolium* C.Y. Hao, H.S. Wu, Y.H. Tan	Asia	China	l	p	t	boreostrop	Pal	e
MAG	Piperales	Piperaceae	*Piper peltatum* L.	South America	S America, C America	h,l	-	t	strop, trop	Neo	e
MON	Alismatales	Araceae	*Alocasia acuminata* Schott	Asia	Bangladesh, China, India, Nepal, SE Asia	h	p	t	boreostrop, trop	Pal	e
MON	Alismatales	Araceae	*Alocasia baginda* Kurniawan & P.C. Boyce	Asia	Borneo	h	p	t	trop	Pal	e
MON	Alismatales	Araceae	*Alocasia chaii* P.C. Boyce	Asia	Borneo	h	p	t	trop	Pal	e
MON	Alismatales	Araceae	*Alocasia cuprea* K.Koch	Asia	Borneo	h	p	t	trop	Pal	e
MON	Alismatales	Araceae	*Alocasia fallax* Schott	Asia	Bangladesh, India	h	p	t	boreostrop	Pal	e
MON	Alismatales	Araceae	*Alocasia fornicata* (Kunth) Schott	Asia	India, Bangladesh, India, Sri Lanka, SE Asia	h	p	t	boreostrop, trop	Pal	e
MON	Alismatales	Araceae	*Alocasia infernalis* P.C. Boyce	Asia	Borneo	h	p	t	trop	Pal	e
MON	Alismatales	Araceae	*Alocasia longiloba* Miq.	Asia	SE Asia	h	p	t	boreostrop, trop	Pal	e
MON	Alismatales	Araceae	*Alocasia melo* A. Hay, P.C. Boyce & K.M. Wong	Asia	Borneo	h	p	t	trop	Pal	e
MON	Alismatales	Araceae	*Alocasia navicularis* (K. Koch & C.D. Bouché) K. Koch & C.D. Bouché	Asia	Bangladesh, China, India, Nepal, SE Asia	h	p	t	boreostrop, trop	Pal	e
MON	Alismatales	Araceae	*Alocasia peltata* M. Hotta	Asia	Borneo	h	p	t	trop	Pal	e
MON	Alismatales	Araceae	*Alocasia perakensis* Hemsl.	Asia	Malaysia, Thailand	h	p	t	boreostrop, trop	Pal	e
MON	Alismatales	Araceae	*Alocasia reginula* A.Hay	Asia	Borneo	h	p	t	trop	Pal	e
MON	Alismatales	Araceae	*Alocasia reversa* N.E. Br.	Asia	Borneo	h	p	t	trop	Pal	e
MON	Alismatales	Araceae	*Amorphophallus konjac* K.Koch	Asia	China, SE Asia	h	p	t	m	Hol	c
MON	Alismatales	Araceae	*Anthurium forgetii* N.E.Br.	South America	Colombia	h	p	t	trop	Neo	e
MON	Alismatales	Araceae	*Anthurium jureianum* Cath. & Olaio	South America	Brazil	h	p	t	austrostrop	Neo	e
MON	Alismatales	Araceae	*Ariopsis peltata* Nimmo	Asia	India	h	p	t	boreostrop, trop	Pal	e
MON	Alismatales	Araceae	*Ariopsis proanthera* N.E. Br.	Asia	Bangladesh, India, Myanmar, Nepal, Thailand	h	p	t	boreostrop, trop	Pal	e
MON	Alismatales	Araceae	*Arisaema caudatum* Engl.	Asia	India	h	p	t	boreostrop, trop	Pal	c
MON	Alismatales	Araceae	*Arisaema ciliatum* H.Li	Asia	China	h	p	t	m	Hol	c
MON	Alismatales	Araceae	*Arisaema concinnum* Schott.	Asia	Bhutan, China, India, Myanmar, Nepal, Tibet	h	p	t	m, boreostrop, trop	Hol, Pal	c
MON	Alismatales	Araceae	*Arisaema fischeri* Manudev & Nampy	Asia	India	h	p	t	boreostrop, trop	Pal	c
MON	Alismatales	Araceae	*Arisaema leschenaultii* Blume	Asia	India, Nepal, Sri Lanka	h	p	t	m, boreostrop, trop	Pal	e
MON	Alismatales	Araceae	*Arisaema nilamburense* Sivad.	Asia	India	h	p	t	boreostrop, trop	Pal	e
MON	Alismatales	Araceae	*Arisaema peltatum* C.E.C. Fisch.	Asia	India	h	p	t	boreostrop, trop	Pal	e
MON	Alismatales	Araceae	*Arisaema subulatum* Manudev & Nampy	Asia	India	h	p	t	boreostrop, trop	Pal	c
MON	Alismatales	Araceae	*Caladium bicolor* Vent.	South America	C America, S America	h	p	t	boreostrop, trop	Neo	e
MON	Alismatales	Araceae	*Caladium clavatum* Hett., Bogner & J. Boos	South America	Ecuador	h	p	t	trop	Neo	e
MON	Alismatales	Araceae	*Caladium humboldtii* (Raf.) Schott	South America	Brazil, Venezuela	h	p	t	trop	Neo	e
MON	Alismatales	Araceae	*Caladium smaragdinum* K. Koch & C.D. Bouché	South America	Colombia, Venezuela	h	p	t	trop	Neo	e
MON	Alismatales	Araceae	*Caladium steudnerifolium* Engl.	South America	Bolovia, Colombia, Ecuador, Peru	h	p	t	trop, austrostrop	Neo	e
MON	Alismatales	Araceae	*Caladium steyermarckii* G.S. Bunting	South America	Venezuela	h	p	t	trop	Neo	e
MON	Alismatales	Araceae	*Colocasia affinis* Schott	Asia	Bangladesh, China, Myanmar, Nepal	h	p	t	boreostrop, m	Pal	e

Clade	Order	Family	Species	Continent	Distribution	Habit		Habitat	Floral zone	Floristic kingdom	Peltation
MON	Alismatales	Araceae	*Colocasia boyceana* Gogoi & Borah	Asia	India	h	p	t	boreostrop, trop	Pal	e
MON	Alismatales	Araceae	*Colocasia esculenta* (L.) Schott	Asia	SE Asia	h	p	t	trop, borestrop, m	Pal	e
MON	Alismatales	Araceae	*Colocasia fallax* Schott	Asia	SE Asia	h	p	t	m, boreostrop	Pal	e
MON	Alismatales	Araceae	*Colocasia hassanii* H. Ara	Asia	Bangladesh	h	p	t	boreostrop	Pal	e
MON	Alismatales	Araceae	*Colocasia lihengiae* C.L. Long & K.M. Liu	Asia	Bangladesh, China, India, Vietnam	h	p	t	boreostrop, trop	Pal	e
MON	Alismatales	Araceae	*Colocasia mannii* Hook. f.	Asia	India, Bangladesh	h	p	t	boreostrop	Pal	e
MON	Alismatales	Araceae	*Colocasia mengalensis* J.T. Yin & H. Li & Z.F. Xu	Asia	China, Laos, Myanmar, Thailand, Vietnam	h	p	t	m, boreostrop, trop	Pal	e
MON	Alismatales	Araceae	*Colocasia oresbia* A. Hay	Asia	Bangladesh, Borneo, India	h	p	t	boreostrop, trop	Pal	e
MON	Alismatales	Araceae	*Remusatia hookeriana* Schott	Asia	China, India, Myanmar, Nepal, Thailand	h	p	t	m, boreostrop, trop	Hol, Pal	e
MON	Alismatales	Araceae	*Remusatia pumila* (D.Don) H.Li & A.Hay	Asia	China, Bangladesh, Nepal, Thailand	h	p	t	boreostrop	Pal	e
MON	Alismatales	Araceae	*Remusatia vivipara* (Roxb.) Schott	Africa, Asia, Australia and Oceania	Africa , Australia, SE Asia	h	p	t	strop, trop	Pal, Aus	e
MON	Alismatales	Araceae	*Remusatia yunnanensis* (H. Li & A. Hay) A. Hay	Asia	China, Taiwan	h	p	t	m, boreostrop	Hol, Pal	e
MON	Alismatales	Araceae	*Steudnera assamica* Hook. f.	Asia	India	h	p	t	boreostrop	Pal	e
MON	Alismatales	Araceae	*Steudnera capitellata* Hook. f.	Asia	Myanmar	h	p	t	boreostrop	Pal	e
MON	Alismatales	Araceae	*Steudnera colocasiifolia* K.Koch	Asia	SE Asia, China	h	p	t	m, boreostrop	Hol, Pal	e
MON	Alismatales	Araceae	*Steudnera colocasioides* Hook. f.	Asia	Bangladesh, India, Myanmar	h	p	t	boreostrop, trop	Pal	e
MON	Alismatales	Araceae	*Steudnera discolor* W. Bull	Asia	India, Bangladesh, Myanmar, Thailand	h	p	t	boreostrop	Pal	e
MON	Alismatales	Araceae	*Steudnera griffithii* (Schott) Hook. f.	Asia	China, India, Myanmar	h	p	t	m, boreostrop, trop	Hol, Pal	e
MON	Alismatales	Araceae	*Steudnera henryana* Engl.	Asia	China, Laos, Vietnam	h	p	t	m, boreostrop, trop	Hol, Pal	e
MON	Alismatales	Araceae	*Steudnera kerrii* Gagnep.	Asia	China, Laos, Thailand, Vietnam	h	p	t	boreostrop	Pal	e
MON	Alismatales	Araceae	*Steudnera virosa* (Roxb.) Prain	Asia	Bangladesh, India	h	p	t	boreostrop, trop	Pal	e
MON	Alismatales	Araceae	*Xanthosoma peltatum* G.S. Bunting	South America	Venezuela	h	p	t	trop	Neo	e
EUD	Proteales	Nelumbonaceae	*Nelumbo lutea* Willd.	North America	S USA	h	p	a	boreostrop, m	Hol	c
EUD	Proteales	Nelumbonaceae	*Nelumbo nucifera* Gaertn.	Asia	India	h	p	a	boreostrop	Pal	c
EUD	Ranunculales	Berberidaceae	*Podophyllum aurantiocaule* Hand.-Mazz.	Asia	China	h	p	t	m	Hol	c
EUD	Ranunculales	Berberidaceae	*Podophyllum delavayi* Franch.	Asia	China	h	p	t	m	Hol	e
EUD	Ranunculales	Berberidaceae	*Podophyllum difforme* Hemsl. & E.H.Wilson	Asia	China, Vietnam	h	p	t	m, boreostrop	Hol, Pal	c
EUD	Ranunculales	Berberidaceae	*Podophyllum glaucescens* J.M.H.Shaw	Asia	China	h	p	t	m	Hol	e
EUD	Ranunculales	Berberidaceae	*Podophyllum hemsleyi* J.M.H. Shaw & Stearn	Asia	China	h	p	t	m, boreostrop	Hol, Pal	c
EUD	Ranunculales	Berberidaceae	*Podophyllum peltatum* L.	North America	N America	h	p	t	b, temp, sm, m	Hol	c
EUD	Ranunculales	Berberidaceae	*Podophyllum pleianthum* Hance	Asia	China, Taiwan	h	p	t	m	Hol	c
EUD	Ranunculales	Berberidaceae	*Podophyllum versipelle* Hance	Asia	China, Vietnam	h	p	t	boreostrop	Pal	c
EUD	Ranunculales	Menispermaceae	*Cissampelos glaberrima* A. St.-Hil.	South America	Tropical S America	h,l	p	t	trop, austrostrop	Neo	e
EUD	Ranunculales	Menispermaceae	*Cissampelos grandifolia* Triana & Planch.	North America, South America	C America, Tropical S America	h,l	p	t	boreostrop, trop, austrostrop	Neo	e
EUD	Ranunculales	Menispermaceae	*Cissampelos hispida* Forman	Asia	Thailand	l	p	t	boreostrop, trop	Pal	e
EUD	Ranunculales	Menispermaceae	*Cissampelos owariensis* P. Beauv. ex DC.	Africa	Tropical Africa	h,l	p	t	boreostrop, trop	Pal	e

Clade	Order	Family	Species	Continent	Distribution	Habit		Habitat	Floral zone	Floristic kingdom	Peltation
EUD	Ranunculales	Menispermaceae	*Cissampelos rigidifolia* (Engl.) Diels	Africa	C African Republic, Sudan, Tanzania, Uganda, Zambia	l	p	t	boreostrop, trop, austrostrop	Pal	e
EUD	Ranunculales	Menispermaceae	*Cissampelos sympodialis* Eichler	South America	Brazil	h,l	p	t	trop, austrostrop	Neo	e
EUD	Ranunculales	Menispermaceae	*Coscinium blumeanum* Miers ex Hook.f. & Thomson	Asia	Malaysia, Thailand, Vietnam	l	p	t	boreostrop, trop	Pal	e
EUD	Ranunculales	Menispermaceae	*Cyclea cauliflora* Merr.	Asia	Philippines, Sulawesi	h,l	p	t	boreostrop, trop	Pal	e
EUD	Ranunculales	Menispermaceae	*Cyclea debiliflora* Miers	Asia	China, India, Vietnam	h,l	p	t	m, boreostrop, trop	Pal	e
EUD	Ranunculales	Menispermaceae	*Cyclea fissicalyx* Dunn	Asia	India	h,l	p	t	boreostrop, trop	Pal	e
EUD	Ranunculales	Menispermaceae	*Cyclea hypoglauca* (Schauer) Diels	Asia	China, Vietnam	h,l	p	t	m, boreostrop, trop	Pal	e
EUD	Ranunculales	Menispermaceae	*Cyclea laxiflora* Miers	Asia	Borneo, Malaysia, Sumatra, Tahiland	h,l	p	t	boreostrop, trop	Pal	e
EUD	Ranunculales	Menispermaceae	*Cyclea peltata* (Lam.) Hook. f. & Thomson	Asia	SE Asia, India	h,l	p	t	boreostrop	Pal	e
EUD	Ranunculales	Menispermaceae	*Cyclea varians* Craib	Asia	Thailand	h,l	p	t	boreostrop, trop	Pal	e
EUD	Ranunculales	Menispermaceae	*Disciphania calocarpa* Standl.	North America	C America	l	p	t	boreostrop	Neo	e
EUD	Ranunculales	Menispermaceae	*Disciphania contraversa* Barneby	South America	Brazil	-	p	t	austrostrop	Neo	e
EUD	Ranunculales	Menispermaceae	*Disciphania hernandia* (Vell.) Barneby	South America	Brazil	h, l	p	t	trop, austrostrop	Neo	e
EUD	Ranunculales	Menispermaceae	*Menispermum dauricum* DC.	Asia	China, Mongolia, Russia, Japan, Korea	h, l	p	t	b, temp, sm, m, boreostrop	Hol, Pal	e
EUD	Ranunculales	Menispermaceae	*Perichasma laetificata* Miers	Africa	midwest Africa	-	p	t	trop	Pal	e
EUD	Ranunculales	Menispermaceae	*Stephania abyssinica* (Quart.-Dill. & A.Rich.) Walp.	Africa	Africa	h	p	t	strop, trop	Pal	e
EUD	Ranunculales	Menispermaceae	*Stephania brevipes* Craib	Asia	Thailand, Vietnam	h	p	t	boreostrop, trop	Pal	e
EUD	Ranunculales	Menispermaceae	*Stephania capitata* (Blume) Spreng.	Asia	Borneo, Jawa, Sunda Islands, Malaysia, Sumatra, Thailand	h	p	t	boreostrop, trop	Pal	e
EUD	Ranunculales	Menispermaceae	*Stephania delavayi* Diels	Asia	China, Myanmar	h	p	t	boreostrop	Pal	e
EUD	Ranunculales	Menispermaceae	*Stephania formanii* Kundu & S. Guha	Australia and Oceania	New Guinea	h	p	t	trop	Pal	e
EUD	Ranunculales	Menispermaceae	*Stephania japonica* (Thunb.) Miers	Asia, Australia and Oceania	Tropical Asia, Subtropical Asia, Australia	h	p	t	boreostrop, trop, austrostrop	Pal, Aus	e
EUD	Ranunculales	Menispermaceae	*Stephania oblata* Craib	Asia	Thailand, Cambodia	h	p	t	boreostrop, trop	Pal	e
EUD	Ranunculales	Menispermaceae	*Stephania pierrei* Diels	Asia	Cambodia, Laos, Thailand, Vietnam	h	p	t	boreostrop, trop	Pal	e
EUD	Ranunculales	Menispermaceae	*Stephania rotunda* Lour.	Asia	India, Western SE Asia	h	p	t	boreostrop, trop	Pal	e
EUD	Ranunculales	Menispermaceae	*Stephania suberosa* Forman	Asia	Cambodia, Thailand	h	p	t	trop	Pal	e
EUD	Ranunculales	Menispermaceae	*Stephania venosa* Spreng.	Asia	SE Asia	h	p	t	boreostrop, trop	Pal	e
EUD	Ranunculales	Ranunculaceae	*Peltocalathos baurii* (MacOwan) Tamura	Africa	South Africa, Lesotho, Eswatini	h	p	t	austr	Cap	c
EUD	Ranunculales	Ranunculaceae	*Ranunculus clypeatus* (Ulbr.) Lourteig	South America	Peru	h	p	t	trop	Neo	e
EUD	Ranunculales	Ranunculaceae	*Ranunculus lyallii* Hook.f.	Australia and Oceania	New Zealand	h	p	t	austr	Ant	c
EUD	Ranunculales	Ranunculaceae	*Thalictrum ichangense* Lecoy. ex Oliv.	Asia	China, Corea, Vietnam	h	p	t	boreostrop, m	Hol, Pal	e
EUD	Ranunculales	Ranunculaceae	*Thalictrum pringlei* S. Watson	North America	Mexico	h	p	t	boreostrop	Neo	e
EUD	Ranunculales	Ranunculaceae	*Thalictrum pseudoichangense* Q.E. Yang & G.H. Zhu	Asia	China	h	p	t	m, boreostrop	Hol, Pal	e
EUD	Ranunculales	Ranunculaceae	*Thalictrum roseanum* B.Boivin	North America	Mexico	h	p	t	boreostrop	Neo	e
EUD	Ranunculales	Ranunculaceae	*Asteropyrum cavaleriei* H.Lév. & Vaniot	Asia	China	h	p	t	m	Hol	e
EUD	Ranunculales	Ranunculaceae	*Asteropyrum peltatum* (Franch.) J.R.Drumm. & Hutch.	Asia	China, Myanmar	h	p	t	boreostrop	Pal	e
EUD-C	Apiales	Apiaceae	*Klotzschia brasiliensis* Cham.	South America	Brazil	h	-	t	austrostrop	Neo	e

Clade	Order	Family	Species	Continent	Distribution	Habit		Habitat	Floral zone	Floristic kingdom	Peltation
EUD-C	Apiales	Apiaceae	*Klotzschia glaziovii* Urb.	South America	Brazil	l	p	t	austrostrop	Neo	e
EUD-C	Apiales	Apiaceae	*Klotzschia rhizophylla* Urb.	South America	Brazil	h	-	t	austrostrop	Neo	e
EUD-C	Apiales	Apiaceae	*Petagnaea gussonei* (Spreng.) Rauschert	Europe	Sicilia	h	p	t	m	Hol	c
EUD-C	Apiales	Araliaceae	*Hydrocotyle bonariensis* Lam.	South America	C America, S America	h	p	t	boreostrop, trop	Neo	c
EUD-C	Apiales	Araliaceae	*Hydrocotyle pusilla* R.Br. ex Rich.	South America	C America, S America	h	p	t	boreostrop, trop	Neo	c
EUD-C	Apiales	Araliaceae	*Hydrocotyle umbellata* L.	South America	C America, S America	h	p	t	boreostrop, trop	Neo	c
EUD-C	Apiales	Araliaceae	*Hydrocotyle verticillata* Thunb.	Africa, South America, North America	N America, S America, S Africa	h	p	a	strop, trop, m	Hol, Neo, Pal	c
EUD-C	Apiales	Araliaceae	*Hydrocotyle vulgaris* L.	Europe	Europe	h	p	a	m, sm, temp, b	Hol	c
EUD-C	Apiales	Araliaceae	*Hydrocotyle yanghuangensis* (Hieron.) Mathias	South America	Ecuador	h	p	t	trop	Neo	e
EUD-C	Apiales	Araliaceae	*Oplopanax japonicus* Nakai	Asia	Japan	l	p	t	t, sm	Hol	e
EUD-C	Apiales	Araliaceae	*Schefflera actinophylla* (Endl.) Harms	Australia and Oceania	Australia, New Guinea	l	p	t	trop, austrostrop	Pal, Aus	e
EUD-C	Apiales	Araliaceae	*Schefflera arboricola* (Hayata) Merr.	Asia	Taiwan	l	p	t	m	Hol	e
EUD-C	Apiales	Araliaceae	*Schefflera digitata* J.R. Forst. & G. Forst.	Australia and Oceania	New Zealand	l	p	t	austrostrop, austr	Aus	e
EUD-C	Asterales	Asteraceae	*Ligularia nelumbifolia* Hand.-Mazz.	Asia	China	h	p	t	sm, m, boreostrop	Hol, Pal	e
EUD-C	Asterales	Asteraceae	*Prenanthes subpeltata* Stebbins	Africa	Ethiopia, Kenya, Rwanda, Democratic Republic of Congo	h	p	t	boreostrop, trop	Pal	e
EUD-C	Asterales	Asteraceae	*Psacalium laxiflorum* Benth.	North America	Mexico	h	p	t	boreostrop	Neo	c
EUD-C	Asterales	Asteraceae	*Psacalium megaphyllum* Rydb.	North America	Mexico	h	p	t	boreostrop	Neo	e
EUD-C	Asterales	Asteraceae	*Senecio oxyriifolius* DC.	Africa	C Africa, S Africa	h	p	t	austr, austrostrop, trop	Cap, Pal	e
EUD-C	Asterales	Asteraceae	*Syneilesis aconitifolia* (Bunge) Maxim.	Asia	China, Japan, Korea, Russia	h	p	t	t, sm, m, boreostrop	Hol, Pal	c
EUD-C	Asterales	Asteraceae	*Syneilesis palmata* (Thunb.) Maxim.	Asia	Korea, Japan	h	p	t	sm	Hol	c
EUD-C	Asterales	Asteraceae	*Psacalium pachyphyllum* (Sch.Bip.) H.Rob. & Brettell	North America	Mexico	h	p	t	boreostrop	Neo	e
EUD-C	Asterales	Asteraceae	*Psacalium peltatum* Cass.	North America	Mexico	h	p	t	boreostrop	Neo	c
EUD-C	Asterales	Asteraceae	*Psacalium pinetorum* (Standl. & Steyerm.) Cuatrec.	North America	Guatemala	h	p	t	boreostrop	Neo	e
EUD-C	Asterales	Asteraceae	*Psacalium putlanum* B.L. Turner	North America	Mexico	h	p	t	boreostrop	Neo	e
EUD-C	Asterales	Asteraceae	*Roldana chapalensis* (S. Watson) H. Rob. & Bretell	North America	Mexico	l	p	t	boreostrop	Neo	e
EUD-C	Asterales	Asteraceae	*Roldana heterogama* (Benth.) H.Rob. & Brettell	North America	Costa Rica, Guatemala, Mexico, Panama	h, l	p	t	boreostrop	Neo	e
EUD-C	Asterales	Asteraceae	*Roldana subpeltata* (Sch. Bip.) H. Rob & Brettell	North America	Mexico	h, l	p	t	boreostrop	Neo	e
EUD-C	Brassicales	Caricaceae	*Jacaratia digitata* (Poepp. & Endl.) Solms	South America	Bolivia, Brazil, Ecuador, Venezuela	l	p	t	strop, trop	Neo	e
EUD-C	Brassicales	Caricaceae	*Jacaratia spinosa* (Aubl.) A.DC.	South America	C America, S America	l	p	t	strop, trop	Neo	e
EUD-C	Brassicales	Tropaeolaceae	*Tropaeolum brachyceras* Hook. & Arn.	South America	Chile	h	p	t	austrostrop	Neo, Ant	e
EUD-C	Brassicales	Tropaeolaceae	*Tropaeolum ciliatum* Ruiz & Pav.	South America	Chile	h	p	t	austrostrop	Neo, Ant	e
EUD-C	Brassicales	Tropaeolaceae	*Tropaeolum cirrhipes* Hook.	South America	Peru	h	-	t	trop	Neo	e
EUD-C	Brassicales	Tropaeolaceae	*Tropaeolum lindenii* Wallis	South America	Colombia, Ecuador	h	-	t	trop	Neo	e
EUD-C	Brassicales	Tropaeolaceae	*Tropaeolum majus* L.	South America	Peru	h	p	t	austrostrop	Neo	e
EUD-C	Brassicales	Tropaeolaceae	*Tropaeolum minus* L.	South America	Ecuador, Peru	h	a	t	austrostrop, trop	Neo	e
EUD-C	Brassicales	Tropaeolaceae	*Tropaeolum pentagonum* Hughes	South America	Bolivia, Venezuela	h	-	t	trop	Neo	e

Clade	Order	Family	Species	Continent	Distribution	Habit		Habitat	Floral zone	Floristic kingdom	Peltation
EUD-C	Brassicales	Tropaeolaceae	*Tropaeolum pentaphyllum* Lam.	South America	Argentina, Brazil, Paraguay, Uruguay	h	p	t	austrostrop	Neo	e
EUD-C	Brassicales	Tropaeolaceae	*Tropaeolum smithii* DC.	South America	N South America	h	p	t	strop, trop	Neo	e
EUD-C	Brassicales	Tropaeolaceae	*Tropaeolum stipulatum* Buchenau & Sodiro	South America	Ecuador, Peru	h	-	t	trop	Neo	e
EUD-C	Brassicales	Tropaeolaceae	*Tropaeolum tuberosum* Ruiz & Pav.	South America	Bolivia, Colombia, Ecuador, Peru	h	p	t	austrostrop, trop	Neo	e
EUD-C	Caryophyllales	Polygonaceae	*Coccoloba acapulcensis* Standl.	North America	C America	l	p	t	boreostrop	Neo	e
EUD-C	Caryophyllales	Polygonaceae	*Coccoloba tiliacea* Lindau	South America	Argentina, Bolivia	l	p	t	trop, austrostrop, austr	Neo	e
EUD-C	Caryophyllales	Polygonaceae	*Persicaria perfoliata* (L.) Gross	Europe, Asia	Turkey, India, E Asia, SE Asia	h	a	t	b, temp, sm, m, boreostrop, trop	Hol, Pal	e
EUD-C	Cornales	Loasaceae	*Nasa peltata* (Spruce ex Urb. & Gilg) Weigend	South America	Ecuador, Peru	h	-	t	trop	Neo	e
EUD-C	Cornales	Loasaceae	*Nasa peltiphylla* (Weigend) Weigend	South America	Colombia, Ecuador	-	-	t	trop	Neo	e
EUD-C	Cucurbitales	Begoniaceae	*Begonia adpressa* Sosef	Africa	Cameroon	h	p	t	trop	Pal	e
EUD-C	Cucurbitales	Begoniaceae	*Begonia atroglandulosa* Sosef	Africa	Congo, Gabon, Democratic Republic of Congo	h	p	t	trop	Pal	e
EUD-C	Cucurbitales	Begoniaceae	*Begonia caroliniifolia* Regel	North America	Mexico, Guatemala, Honduras	h	p	t	boreostrop	Neo	c
EUD-C	Cucurbitales	Begoniaceae	*Begonia clypeifolia* Hook. f.	Africa	WC Africa	h	p	t	trop	Pal	e
EUD-C	Cucurbitales	Begoniaceae	*Begonia conchifolia* A.Dietr.	South America	Costa Rica, El Salvador, Panamá	h	p	t	trop	Neo	e
EUD-C	Cucurbitales	Begoniaceae	*Begonia dodsonii* L.B.Sm. & Wassh.	South America	Ecuador	h,l	p	t	trop	Neo	e
EUD-C	Cucurbitales	Begoniaceae	*Begonia egregia* N.E.Br.	South America	Brazil	-	-	t	austrostrop	Neo	e
EUD-C	Cucurbitales	Begoniaceae	*Begonia ferramica* N.Hallé	Africa	Gabon	h	p	t	trop	Pal	e
EUD-C	Cucurbitales	Begoniaceae	*Begonia geminiflora* L.B.Sm. & Wassh.	South America	Ecuador	-	-	t	trop	Pal	e
EUD-C	Cucurbitales	Begoniaceae	*Begonia goegoensis*N.E.Br.	Asia	Sumatra	h	p	t	trop	Pal	e
EUD-C	Cucurbitales	Begoniaceae	*Begonia hitchcockii* Irmsch.	South America	Ecuador	h,l	p	t	trop	Neo	e
EUD-C	Cucurbitales	Begoniaceae	*Begonia josephi* A.DC.	Asia	Myanmar, Nepal, Tibet, S China	h	p	t	boreostrop	Pal	e
EUD-C	Cucurbitales	Begoniaceae	*Begonia kellermannii* C.DC.	South America	S America	h	p	t	trop	Neo	e
EUD-C	Cucurbitales	Begoniaceae	*Begonia lacunosa* Warb.	Africa	Cameroon, Congo, Zaïre	h	p	t	trop	Pal	e
EUD-C	Cucurbitales	Begoniaceae	*Begonia lubbersii* É.Morren	South America	Brazil	h,l	p	t	austrostrop	Neo	e
EUD-C	Cucurbitales	Begoniaceae	*Begonia marojejyensis* Humbert	Africa	Madagascar	-	-	t	trop, austrostrop	Pal	e
EUD-C	Cucurbitales	Begoniaceae	*Begonia microsperma* Warb.	Africa	Cameroon, Equatorial Guinea	h	p	t	trop	Pal	e
EUD-C	Cucurbitales	Begoniaceae	*Begonia nelumbiifolia* Cham. & Schltdl.	South America	South America	h	p	t	austrostrop	Neo	e
EUD-C	Cucurbitales	Begoniaceae	*Begonia peltata* Otto & Dietr.	South America	Guatemala, Honduras, Mexico	h,l	p	t	boreostrop	Neo	e
EUD-C	Cucurbitales	Begoniaceae	*Begonia quadrialata* Warb.	Africa	midwest Africa	h	p	t	trop	Pal	e
EUD-C	Cucurbitales	Begoniaceae	*Begonia rhizocaulis*(Klotzsch) A. DC.	South America	S America	h	p	t	trop	Neo	e
EUD-C	Cucurbitales	Begoniaceae	*Begonia scapigera* Hook.f.	Africa	midwest Africa	l	p	t	trop	Pal	e
EUD-C	Cucurbitales	Begoniaceae	*Begonia sudjanae* C.-A. Jansson	Asia	Sumatra	h	p	t	trop	Pal	e
EUD-C	Cucurbitales	Begoniaceae	*Begonia thiemei* C.DC.	North America	Mexico, Honduras, Guatemala	h	p	t	boreostrop	Neo	e
EUD-C	Ericales	Balsaminaceae	*Impatiens begonioides* Eb. Fisch. & Raheliv.	Africa	Madagascar	h	-	t	trop, austrostrop	Pal	e
EUD-C	Fabales	Fabaceae	*Lupinus albifrons* Benth.	North America	USA	h,l	p	t	sm, m, boreostrop	Hol	e
EUD-C	Fabales	Fabaceae	*Lupinus angustifolius* L.	Europe	Mediterranean area	h	a	t	sm, m	Hol, Pal	e
EUD-C	Fabales	Fabaceae	*Lupinus arboreus* Sims	North America	USA	h,l	p	t	sm, m	Hol	e

Clade	Order	Family	Species	Continent	Distribution	Habit		Habitat	Floral zone	Floristic kingdom	Peltation
EUD-C	Fabales	Fabaceae	*Lupinus cosentinii* Guss.	Europe	Mediterranean area	h	p,a	t	m	Hol	e
EUD-C	Fabales	Fabaceae	*Lupinus digitatus* Forssk.	Africa	Algeria, Egypt, Libya	h	a	t	m, boreostrop	Hol, Pal	e
EUD-C	Fabales	Fabaceae	*Lupinus elegans* Kunth	North America		h,l	p	t	boreostrop	Neo	e
EUD-C	Fabales	Fabaceae	*Lupinus gussoneanus* J.Agardh	Europe	Mediterranean area	h	a	t	sm, m	Hol	e
EUD-C	Fabales	Fabaceae	*Lupinus leucophyllus* Douglas ex Lindl.	North America	USA	h	p	t	temp, sm, m	Hol	e
EUD-C	Fabales	Fabaceae	*Lupinus luteus* L.	Europe	Italy, Portugal, Spain	h	a	t	sm, m	Hol	e
EUD-C	Fabales	Fabaceae	*Lupinus mutabilis* Sweet	South America	Bolivia, Colombia, Ecuador	h,l	p	t	trop	Neo	e
EUD-C	Fabales	Fabaceae	*Lupinus palaestinus* Boiss.	Europe	Israel, Egypt	h	a	t	m, boreostrop	Hol	e
EUD-C	Fabales	Fabaceae	*Lupinus perennis* L.	North America	USA	h	p	t	b, temp, sm	Hol	e
EUD-C	Fabales	Fabaceae	*Lupinus polyphyllus* Lindl.	North America	USA	h	p	t	b, temp, sm, m	Hol	e
EUD-C	Fabales	Fabaceae	*Lupinus subcarnosus* Hook.	North America	USA, Mexico	h	a	t	boreostrop	Hol, Neo	e
EUD-C	Fabales	Fabaceae	*Lupinus succulentus* Douglas ex K.Koch	North America	USA, Mexico	h	a	t	boreostrop, m	Hol, Neo	e
EUD-C	Gentianales	Apocynaceae	*Hoya imbricata* Decne.	Asia	Philippines, Sulawesi	h	p	t	boreostrop, trop	Pal	c
EUD-C	Gentianales	Apocynaceae	*Macropharynx abnorma* J.F. Morales, M.E. Endress & Liede	South America	Peru	h, l	p	t	trop	Neo	e
EUD-C	Gentianales	Apocynaceae	*Macropharynx colombiana* (Woodson) J.F. Morales, M.E. Endress & Liede	South America	Colombia, Venezuela	h, l	p	t	trop	Neo	e
EUD-C	Gentianales	Apocynaceae	*Macropharynx conflictiva* (J.F. Morales) J.F. Morales, M.E. Endress & Liede	South America	Peru	h, l	p	t	trop	Neo	e
EUD-C	Gentianales	Apocynaceae	*Macropharynx gigantea* (Woodson) J.F. Morales, M.E. Endress & Liede	South America	Peru, Bolivia	h, l	p	t	trop, austrostrop	Neo	e
EUD-C	Gentianales	Apocynaceae	*Macropharynx isthmica* (Woodson) J.F. Morales, M.E. Endress & Liede	North America, South America	Colombia, Costa Rica, Panamá, Venezuela	h, l	p	t	boreostrop, trop	Neo	e
EUD-C	Gentianales	Apocynaceae	*Macropharynx macrocalyx* (Müll. Arg.) J.F. Morales, M.E. Endress & Liede	South America	Brazil, Paraguay	h, l	p	t	trop, austrostrop	Neo	e
EUD-C	Gentianales	Apocynaceae	*Macropharynx peltata* (Vell.) J.F. Morales, M.E. Endress & Liede	South America	Argentina, Brazil, Paraguay	h, l	p	t	trop, austrostrop	Neo	e
EUD-C	Gentianales	Apocynaceae	*Macropharynx peruviana* (Woodson) J.F. Morales, M.E. Endress & Liede	South America	Peru	h, l	p	t	trop	Neo	e
EUD-C	Gentianales	Apocynaceae	*Macropharynx pulchra* (Miers) J.F. Morales, M.E. Endress & Liede	South America	Brazil	h, l	p	t	trop, austrostrop	Neo	e
EUD-C	Gentianales	Apocynaceae	*Macropharynx venusta*(J.F. Morales) J.F. Morales, M.E. Endress & Liede	South America	Ecuador	h, l	p	t	trop	Neo	e
EUD-C	Gunnerales	Gunneraceae	*Gunnera antioquensis* L.E. Mora	South America	Colombia	h	p	t	trop	Neo	e
EUD-C	Gunnerales	Gunneraceae	*Gunnera brephogea* Linden & André	South America	Colombia, Ecuador, Peru	h	p	t	trop	Neo	c
EUD-C	Gunnerales	Gunneraceae	*Gunnera peltata* Phil.	South America	Juan Fernández Is.	h	p	t	austr	Ant	c
EUD-C	Gunnerales	Gunneraceae	*Gunnera quitoensis* L.E. Mora	South America	Ecuador	h	p	t	trop	Neo	e
EUD-C	Gunnerales	Gunneraceae	*Gunnera silvioana* L.E. Mora	South America	Colombia, Ecuador	h	p	t	trop	Neo	e
EUD-C	Lamiales	Gesneriacaea	*Cyrtandra toviana* F. Br.	Australia and Oceania	Marquesas	l	p	t	trop	Pal	e
EUD-C	Lamiales	Gesneriacaea	*Cyrtandra wawrae* C.B. Clarke	Australia and Oceania	Hawaii	-	-	t	trop	Pal	e
EUD-C	Lamiales	Gesneriacaea	*Drymonia peltata* (Oliv.) H.E. Moore	North America	Costa Rica	-	-	t	boreostrop	Neo	e
EUD-C	Lamiales	Gesneriacaea	*Metapetrocosmea peltata* (Merr. & Chun) W.T. Wand	Asia	China	h	p	t	boreostrop	Pal	e
EUD-C	Lamiales	Gesneriacaea	*Paraboea peltifolia* D. Fang & L. Zeng	Asia	China	h	p	t	m, boreostrop	Hol, Pal	e
EUD-C	Lamiales	Gesneriacaea	*Paraboea yunfuensis* F. Wen & Y.G. Wie	Asia	China	h	p	t	m, boreostrop	Hol, Pal	e
EUD-C	Lamiales	Gesneriacaea	*Petrocosmea huanjiangensis* Yan Liu & W.B. Xu	Asia	China	h	p	t	m, boreostrop	Hol, Pal	e
EUD-C	Lamiales	Gesneriacaea	*Petrocosmea pubescens* D.J. Middleton & Triboun	Asia	Thailand	h	p	t	boreostrop, trop	Pal	e

Clade	Order	Family	Species	Continent	Distribution	Habit		Habitat	Floral zone	Floristic kingdom	Peltation
EUD-C	Lamiales	Gesneriacaea	*Sinningia tuberosa* (Mart.) H.E. Moore	South America	Brazil	h	p	t	austrostrop	Neo	e
EUD-C	Lamiales	Gesneriacaea	*Streptocarpus mandrerensis* Humbert	Africa	Madagascar	h	-	t	trop, austrostrop	Pal	e
EUD-C	Lamiales	Gesneriacaea	*Streptocarpus peltatus* Randrian., Phillipson, Lowry & Mich. Möller	Africa	Madagascar	h	p	t	trop, austrostrop	Pal	e
EUD-C	Lamiales	Gesneriacaea	*Trichodrymonia peltatifolia* (J.L. Clark & M.M. More) M.M. Mora & J.L. Clark	North America	Panamá	h	-	t	boreostrop	Neo	e
EUD-C	Lamiales	Lentibulariaceae	*Utricularia nelumbifolia* Gardn.	South America	Brazil	h	p	a	austrostrop	Neo	c
EUD-C	Lamiales	Lentibulariaceae	*Utricularia pubescens* Sm.	Africa, South America, Asia	S America, Africa, India	h	a	t	strop, trop	Neo, Pal	c
EUD-C	Malpighiales	Euphorbiaceae	*Endospermum moluccanum* (Teijsm. & Binn.) Kurz	Asia, Australia and Oceania	Indonesia, New Guinea, Solomon Islands	l	p	t	trop	Pal	e
EUD-C	Malpighiales	Euphorbiaceae	*Jatropha podagrica* Hook.	South America	C America	h, l	p	t	trop	Neo	e
EUD-C	Malpighiales	Euphorbiaceae	*Macaranga bancana* (Miq.) Müll.Arg.	Asia	Thailand, Malesia	l	p	t	boreostrop, trop	Pal	e
EUD-C	Malpighiales	Euphorbiaceae	*Macaranga cuspidata Boivin ex Baill.*	Africa	Madagascar	l	p	t	trop, austrostrop	Pal	e
EUD-C	Malpighiales	Euphorbiaceae	*Macaranga magna* Turrill	Australia and Oceania	Fiji	l	p	t	austrostrop	Aus	e
EUD-C	Malpighiales	Euphorbiaceae	*Macaranga tanarius* (L.) Mull.Arg.	Asia, Australia and Oceania	SE Asia, Australia	l	p	t	strop, trop	Pal, Aus	e
EUD-C	Malpighiales	Euphorbiaceae	*Macaranga thompsonii* Merr.	Asia	S Asia	l	p	t	trop	Pal	e
EUD-C	Malpighiales	Euphorbiaceae	*Mallotus floribundus* (Blume) Müll. Arg.	Asia	SE Asia	l	p	t	boreostrop, trop	Pal	e
EUD-C	Malpighiales	Euphorbiaceae	*Mallotus lackeyi* Elmer	Asia	Borneo, Philippines	l	p	t	trop	Pal	e
EUD-C	Malpighiales	Euphorbiaceae	*Mallotus peltatus* (Geiseler) Müll. Arg.	Asia	China, India, SE Asia	l	p	t	m, boreostrop, trop	Pal	e
EUD-C	Malpighiales	Euphorbiaceae	*Mallotus surculosus* P.I. Forst.	Australia and Oceania	Australia	l	p	t	austrostrop	Aus	e
EUD-C	Malpighiales	Euphorbiaceae	*Mallotus thorelii* Gagnep.	Asia	Cambodia, China, Laos, Thailand, Vietnam	l	p	t	m, boreostrop, trop	Pal	e
EUD-C	Malpighiales	Euphorbiaceae	*Manihot fabianae* M. Mend.	South America	Bolivia	l	p	t	trop, austrostrop	Neo	e
EUD-C	Malpighiales	Euphorbiaceae	*Manihot mirabilis* Pax	South America	Paraguay	l	p	t	trop, austrostrop	Neo	e
EUD-C	Malpighiales	Euphorbiaceae	*Manihot peltata* Pohl	South America	Brazil	l	p	t	trop, austrostrop	Neo	e
EUD-C	Malpighiales	Euphorbiaceae	*Megistostigma peltatum* (J.J. Sm.) Croizat	Asia	Jawa, Sumatra	l	p	t	trop	Pal	e
EUD-C	Malpighiales	Euphorbiaceae	*Meineckia peltata* (Hutch.) G.L. Webster	Africa	Madagascar	l	p	t	trop, austrostrop	Pal	e
EUD-C	Malpighiales	Euphorbiaceae	*Ricinus communis* L.	Africa	Eritrea, Ethiopia, Somalia	h, l	a, p	t	boreostrop	Pal	e
EUD-C	Malpighiales	Euphorbiaceae	*Endospermum peltatum* Merr.	Asia	South East Asia	l	p	t	trop	Pal	e
EUD-C	Malpighiales	Euphorbiaceae	*Homalanthus grandifolius* Ridl.	Asia	Borneo, Sumatra	l	p	t	trop	Pal	e
EUD-C	Malpighiales	Euphorbiaceae	*Homalanthus macradenius* Pax & K. Hoffm.	Asia	Philippines	l	p	t	boreostrop, trop	Pal	e
EUD-C	Malpighiales	Euphorbiaceae	*Jatropha hernandiifolia* Vent.	North America	Dominican Republic, Haiti, Puerto Rico	l	p	t	boreostrop	Neo	e
EUD-C	Malpighiales	Euphorbiaceae	*Jatropha nudicaulis* Benth.	South America	Colombia, Ecuador	l	p	t	trop	Neo	e
EUD-C	Malpighiales	Euphorbiaceae	*Jatropha peltata* Sessé	North America	Mexico	h, l	p	t	boreostrop	Neo	e
EUD-C	Malpighiales	Euphorbiaceae	*Jatropha weberbaueri* Pax & K. Hoffm.	South America	Peru	-	-	t	trop	Neo	e
EUD-C	Malpighiales	Passifloraceae	*Adenia penangiana* (Wall. ex G. Don) W.J. de Wilde	Asia	SE Asia	h	p	t	boreostrop, trop	Pal	e
EUD-C	Malpighiales	Passifloraceae	*Passiflora coriacea* Juss.	South America	C America, S America	h	p	t	boreostrop, trop	Neo	e
EUD-C	Malpighiales	Passifloraceae	*Passiflora guatemalensis* S. Watson	North America, South America	C America, Colombia, Venezuela	h	p	t	boreostrop, trop	Neo	e
EUD-C	Malpighiales	Passifloraceae	*Passiflora rubrotincta* Killip	South America	Bolivia	h	p	t	trop, austrostrop	Neo	e
EUD-C	Malpighiales	Passifloraceae	*Passiflora spectabilis* Killip	South America	Peru	h	p	t	trop	Neo	e
EUD-C	Malpighiales	Phyllanthaceae	*Astrocasia peltata* Standl.	North America	Costa Rica, Mexico	l	p	t	boreostrop	Neo	e
EUD-C	Malpighiales	Salicaceae	*Xylosma peltata* (Sleumer) Lescot	Australia and Oceania	New Caledonia	l	p	t	austrostrop	Aus	e

Clade	Order	Family	Species	Continent	Distribution	Habit		Habitat	Floral zone	Floristic kingdom	Peltation
EUD-C	Malvales	Dipterocarpaceae	*Shorea peltata* Symington	Asia	Borneo, Malaysia, Sumatra	l	p	t	Trop	Pal	e
EUD-C	Malvales	Malvaceae	*Brownlowia ferruginea* Kosterm.	Asia	Borneo	l	p	t	trop	Pal	e
EUD-C	Malvales	Malvaceae	*Brownlowia havilandii* Stapf	Asia	Borneo	l	p	t	trop	Pal	e
EUD-C	Malvales	Malvaceae	*Brownlowia helferiana* Pierre	Asia	Malaysia, Myanmar, Thailand	l	p	t	boreostrop, trop	Pal	e
EUD-C	Malvales	Malvaceae	*Brownlowia peltata* Benth.	Asia	Borneo, Myanmar, Thailand, Vietnam	l	p	t	boreostrop, trop	Pal	e
EUD-C	Malvales	Malvaceae	*Brownlowia stipulata* Kosterm.	Asia	Borneo	l	p	t	trop	Pal	e
EUD-C	Malvales	Malvaceae	*Pterospermum acerifolium (L.) Willd.*	Asia	India, SE Asia	h	p	t	boreostrop, trop	Pal	e
EUD-C	Myrtales	Melastomataceae	*Catanthera peltata* M.P. Nayar	Asia	Borneo	-	-	t	trop	Pal	e
EUD-C	Myrtales	Melastomataceae	*Conostegia peltata* (Almeda) Kriebel	North America	Panama	l	p	t	trop	Neo	e
EUD-C	Myrtales	Melastomataceae	*Gravesia peltata* H. Perrier	Africa	Madagascar	h	p	t	trop, austrostrop	Pal	e
EUD-C	Myrtales	Melastomataceae	*Leandra peltata* Wurdack	South America	Peru	l	p	t	trop	Neo	e
EUD-C	Myrtales	Melastomataceae	*Phyllagathis beccariana* (Cogn.) M.P. Nayar	Asia	Borneo	h	p	t	trop	Pal	e
EUD-C	Myrtales	Melastomataceae	*Phyllagathis peltata* Stapf ex Ridl.	Asia	Borneo	h	p	t	trop	Pal	e
EUD-C	Oxalidales	Oxalidaceae	*Oxalis adenophylla* Gillies ex Hook. & Arn.	South America	Argentina, Chile	h	p	t	austrostrop, austr	Neo	c
EUD-C	Oxalidales	Oxalidaceae	*Oxalis articulata* Savigny	South America	Argentina, Brazil, Uruguay	h	p	t	austrostrop	Neo	c
EUD-C	Oxalidales	Oxalidaceae	*Oxalis bowiei* W.T.Aiton ex G.Don	Africa	South Africa	h	p	t	austr	Cap	c
EUD-C	Oxalidales	Oxalidaceae	*Oxalis carolina* J. Suda & Sudová	Africa	South Africa	h	p	t	austr	Cap	c
EUD-C	Oxalidales	Oxalidaceae	*Oxalis corniculata* L.	South America	C America	h	a	t	trop	Neo	c
EUD-C	Oxalidales	Oxalidaceae	*Oxalis decaphylla* Kunth	North America	Mexico	h	p	t	boreostrop	Neo	c
EUD-C	Oxalidales	Oxalidaceae	*Oxalis latifolia* Kunth	North America, South America	USA, Mexico, C America, N South America	h	p	t	m, boreostrop, trop, austrostrop	Hol, Neo	c
EUD-C	Oxalidales	Oxalidaceae	*Oxalis leucolepis* Diels	Asia	China, Nepal	h	p	t	boreostrop, m	Pal	c
EUD-C	Oxalidales	Oxalidaceae	*Oxalis nelsonii* (Small) R. Kunth	North America	Honduras, Mexico	h	p	t	boreostrop	Neo	c
EUD-C	Oxalidales	Oxalidaceae	*Oxalis stricta* L.	Asia, North America	N America, E Asia	h	a	t	m, sm, temp	Hol	c
EUD-C	Oxalidales	Oxalidaceae	*Oxalis triangularis* A.St.-Hil.	South America	S America	h	p	t	austrostrop, trop	Neo	c
EUD-C	Oxalidales	Oxalidaceae	*Oxalis tuberosa* Molina	South America	Argentina, Bolivia, Colombia, Ecuador, Peru	h	p	t	trop, austrostrop, austr	Neo	c
EUD-C	Rosales	Moraceae	*Dorstenia belizensis* C.C. Berg	North America	Belize	h	p	t	boreostrop	Neo	e
EUD-C	Rosales	Moraceae	*Dorstenia erythrandra* C. Wright ex Griseb.	North America	Cuba, Dominican Republic, Haiti	h	p	t	boreostrop	Neo	e
EUD-C	Rosales	Moraceae	*Dorstenia flagellifera* Urb. & Ekman	North America	Haiti	h	p	t	boreostrop	Neo	e
EUD-C	Rosales	Moraceae	*Dorstenia jamaicensis* Britton	North America	Jamaica	h	p	t	boreostrop	Neo	e
EUD-C	Rosales	Moraceae	*Dorstenia nummularia* Urb. & Ekman	North America	Cuba	h	p	t	boreostrop	Neo	e
EUD-C	Rosales	Moraceae	*Dorstenia peltata* Spreng.	North America	Cuba, Dominican Republic	h	p	t	boreostrop	Neo	e
EUD-C	Rosales	Moraceae	*Dorstenia petrae* C. Wright ex Griseb.	North America	Cuba	h	p	t	boreostrop	Neo	e
EUD-C	Rosales	Moraceae	*Dorstenia roigii* Britton	North America	Cuba	h	p	t	boreostrop	Neo	e
EUD-C	Rosales	Urticaceae	*Cecropia albicans* Trécil	South America	Peru	l	p	t	trop	Neo	e
EUD-C	Rosales	Urticaceae	*Cecropia distachya* Huber	South America	N South America	l	p	t	trop, austrostrop	Neo	e
EUD-C	Rosales	Urticaceae	*Cecropia elongata* Rusby	South America	Bolivia	l	p	t	trop, austrostrop	Neo	e
EUD-C	Rosales	Urticaceae	*Cecropia ficifolia* Warb. ex Snethl.	South America	N South America	l	p	t	trop, austrostrop	Neo	e
EUD-C	Rosales	Urticaceae	*Cecropia latiloba* Miq.	South America	N South America	l	p	t	trop, austrostrop	Neo	e
EUD-C	Rosales	Urticaceae	*Cecropia obtusifolia* Bertol.	North America, South America	C America, Colombia, Ecuador	l	p	t	boreostrop, trop	Neo	e

Clade	Order	Family	Species	Continent	Distribution	Habit		Habitat	Floral zone	Floristic kingdom	Peltation
EUD-C	Rosales	Urticaceae	*Cecropia palmata* Willd.	South America	N South America	l	p	t	trop, austrostrop	Neo	e
EUD-C	Rosales	Urticaceae	*Cecropia peltata* L.	South America	C America	l	p	t	boreostrop, trop	Neo	e
EUD-C	Rosales	Urticaceae	*Cecropia virgusa* Cuatrec.	South America	Colombia, Ecuador, Panamá	l	p	t	boreostrop, trop	Neo	e
EUD-C	Rosales	Urticaceae	*Dendrocnide moroides* (Wedd.) Chew	Asia, Australia and Oceania	Australia, Lesser Sunda Islands, Vanuatu	l	p	t	trop, austrostrop, austr	Pal, Aus	e
EUD-C	Rosales	Urticaceae	*Dendrocnide peltata* (Blume) Miq.	Asia, Australia and Oceania	New Guinea, Malesia	l	p	t	trop	Pal	e
EUD-C	Rosales	Urticaceae	*Elatostema muluense* Rodda & A.K. Monro	Asia	Borneo	h	p	t	trop	Pal	e
EUD-C	Rosales	Urticaceae	*Elatostema peltifolium* (Ridl.) H.J.P. Winkl.	Australia and Oceania	New Guinea	h	-	t	trop	Pal	e
EUD-C	Rosales	Urticaceae	*Musanga cecropioides* R. Br. ex Tedlie	Africa	W and C Africa	l	p	t	trop	Pal	c
EUD-C	Rosales	Urticaceae	*Musanga leo-errerae* Hauman & J. Léonard	Africa	C Africa	l	p	t	trop	Pal	c
EUD-C	Rosales	Urticaceae	*Pilea nonggangensis* Y.G. Wei, L.F. Fu & A.K. Monro	Asia	China	h	p	t	m, boreostrop	Hol, Pal	e
EUD-C	Rosales	Urticaceae	*Pilea panzhihuaensis* C.J. Chen, A.K. Monro & L. Chen	Asia	China	h	p	t	m, boreostrop	Hol, Pal	e
EUD-C	Rosales	Urticaceae	*Pilea peltata* Hance	Asia	China, Vietnam	h	p	t	m, boreostrop, trop	Hol, Pal	e
EUD-C	Rosales	Urticaceae	*Pilea peperomioides* Diels	Asia	China	h	p	t	m	Hol	e
EUD-C	Rosales	Rosaceae	*Rubus peltatus* Maxim.	Asia	China, Japan	h	p	t	sm	Hol	e
EUD-C	Saxifragales	Crassulaceae	*Kalanchoe beharensis* Drake	Africa	Madagascar	h	p	t	trop, austrostrop	Pal	e
EUD-C	Saxifragales	Crassulaceae	*Kalanchoe nyikae* Engl.	Africa	Kenya, Tanzania	h	p	t	trop	Pal	e
EUD-C	Saxifragales	Crassulaceae	*Umbilicus botryoides* Hochst. ex A.Rich.	Africa	Africa	h	p	t	boreostrop, trop	Pal	c
EUD-C	Saxifragales	Crassulaceae	*Umbilicus horizontalis* DC.	Africa, Europe	S Europe, N Africa	h	p	t	m	Hol	c
EUD-C	Saxifragales	Crassulaceae	*Umbilicus luteus* Webb & Berthel.	Europe	Mediterranean region	h	p	t	m, sm	Hol	c
EUD-C	Saxifragales	Crassulaceae	*Umbilicus rupestris* (Salisb.) Dandy	Africa, Europe	Europe, Africa	h	p	t	m, sm	Hol	c
EUD-C	Saxifragales	Saxifragaceae	*Astilboides tabularis* Engl.	Asia	China	h	p	t	sm, temp	Hol	c
EUD-C	Saxifragales	Saxifragaceae	*Chrysosplenium peltatum* Turcz.	Asia	Mongolia, Russia	h	p	t	t, sm, m	Hol	e
EUD-C	Saxifragales	Saxifragaceae	*Darmera peltata* (Torr.) Voss	North America	USA	h	p	t	m	Hol	c
EUD-C	Saxifragales	Saxifragaceae	*Peltoboykinia tellimoides* (Maxim.) H. Hara	Asia	China, Japan	h	p	t	t, sm, m	Hol, Pal	e
EUD-C	Saxifragales	Saxifragaceae	*Peltoboykinia watanabei* (Yatabe) H. Hara	Asia	Japan	h	p	t	t, sm, m	Hol	e
EUD-C	Saxifragales	Saxifragaceae	*Rodgersia aesculifolia* Batalin	Asia	China, Mongolia	h	p	t	sm, m, boreostrop	Hol, Pal	c
EUD-C	Saxifragales	Saxifragaceae	*Rodgersia podophylla* A.Gray	Asia	Japan, Korea	h	p	t	sm	Hol	c
EUD-C	Solanales	Convolvulaceae	*Decalobanthus elmeri* (Merr.) A.R. Simoes & Staples	Asia	Borneo	l	p	t	trop	Neo	e
EUD-C	Solanales	Convolvulaceae	*Decalobanthus peltatus* (L.) A.R. Simoes & Staples	Asia, Australia and Oceania	SE Asia, Australia, Madagascar	l	p	t	trop, austrostrop	Pal, Aus	e
EUD-C	Solanales	Solanaceae	*Nothocestrum peltatum* Skottsb.	Australia and Oceania	Hawaii	l	p	t	trop	Pal	e
