# Supplementary figures and images for: Strengthening Structures in the Petiole–Lamina Junction of Peltate Leaves
Source: Biomimetics (Basel). 2021 Apr 2;6(2):25. doi: 10.3390/biomimetics6020025 (PMC8167582; doi:10.3390/biomimetics6020025)

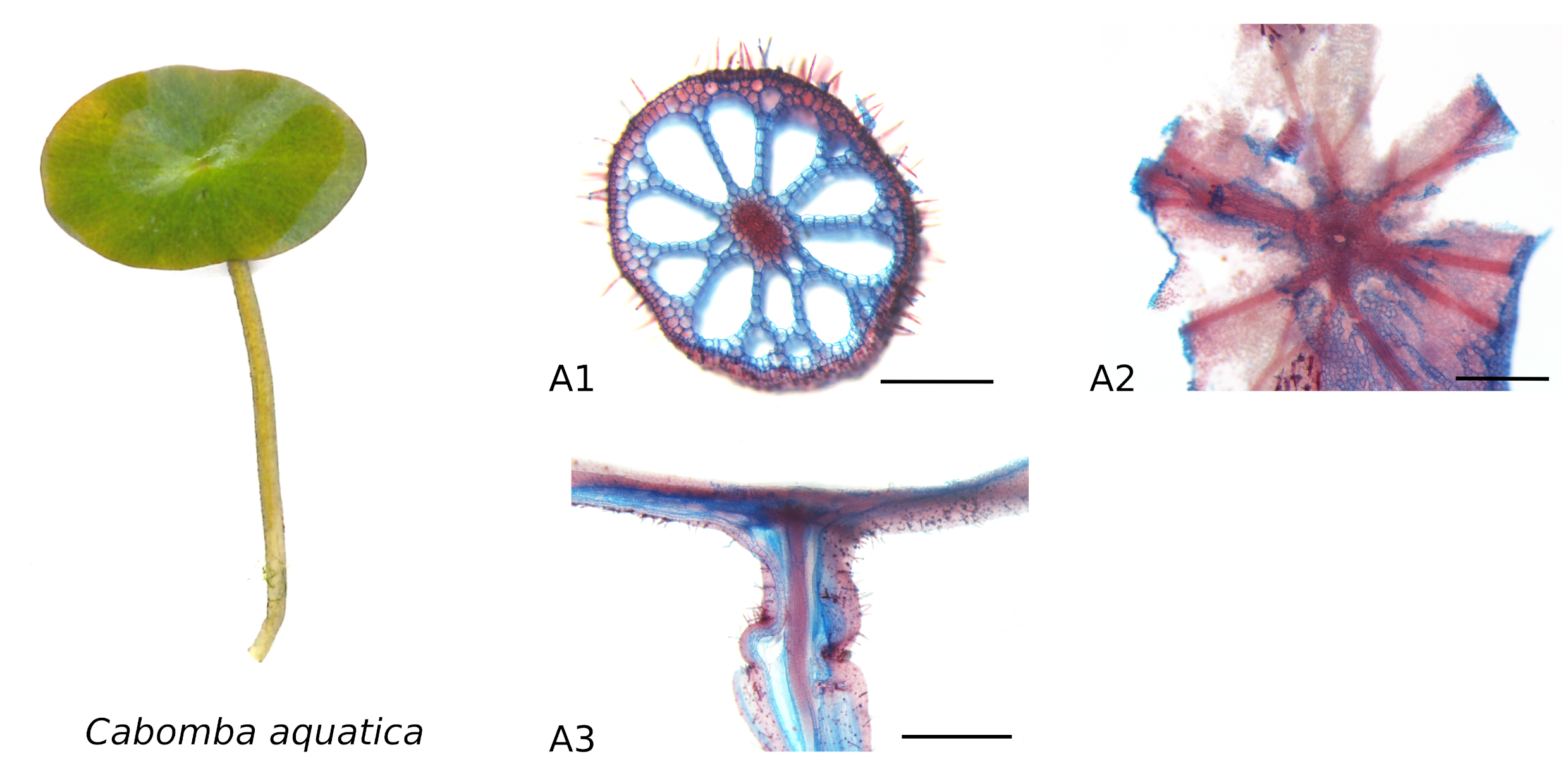

Supplement: Supplementary file 1 [file biomimetics-06-00025-s001.zip › Figure S1.tif]

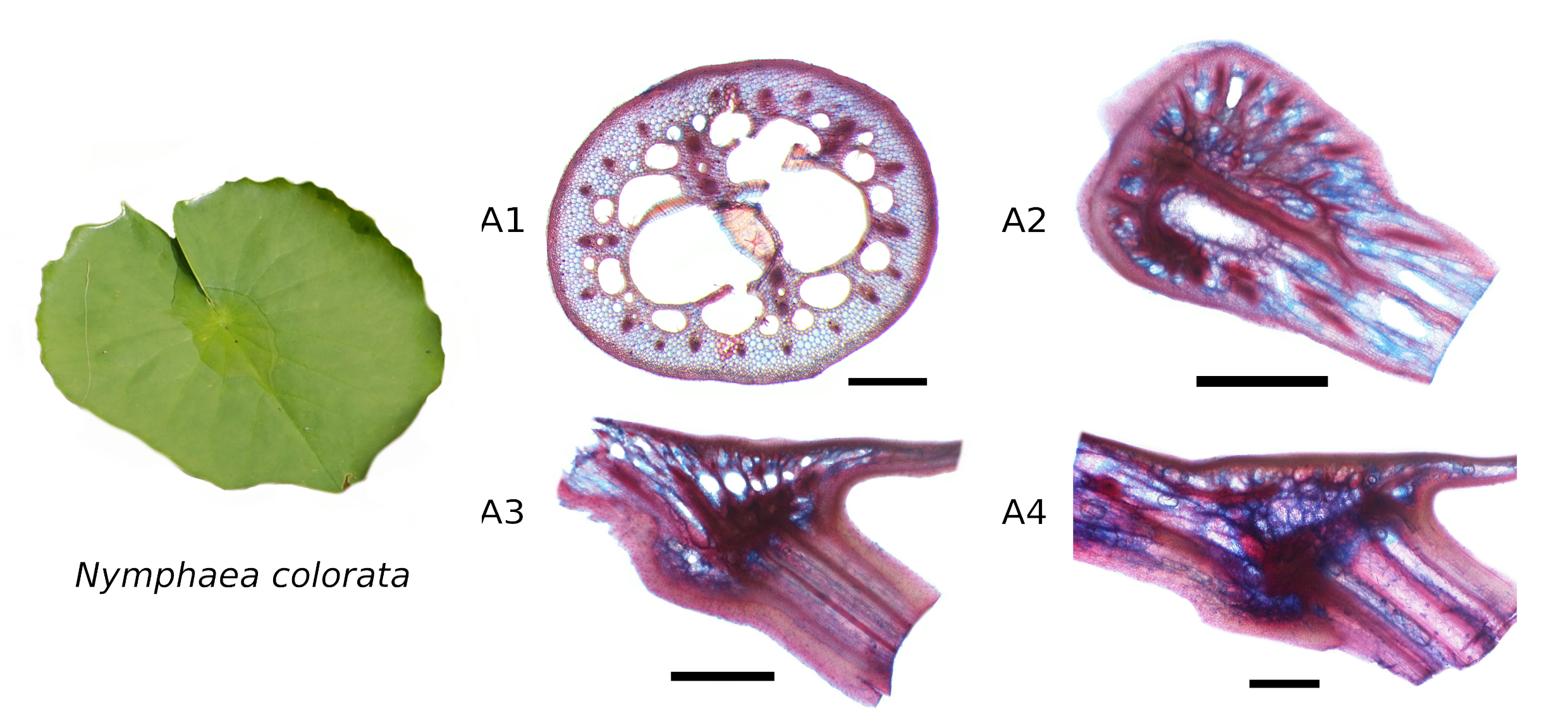

Supplement: Supplementary file 1 [file biomimetics-06-00025-s001.zip › Figure S2.tif]

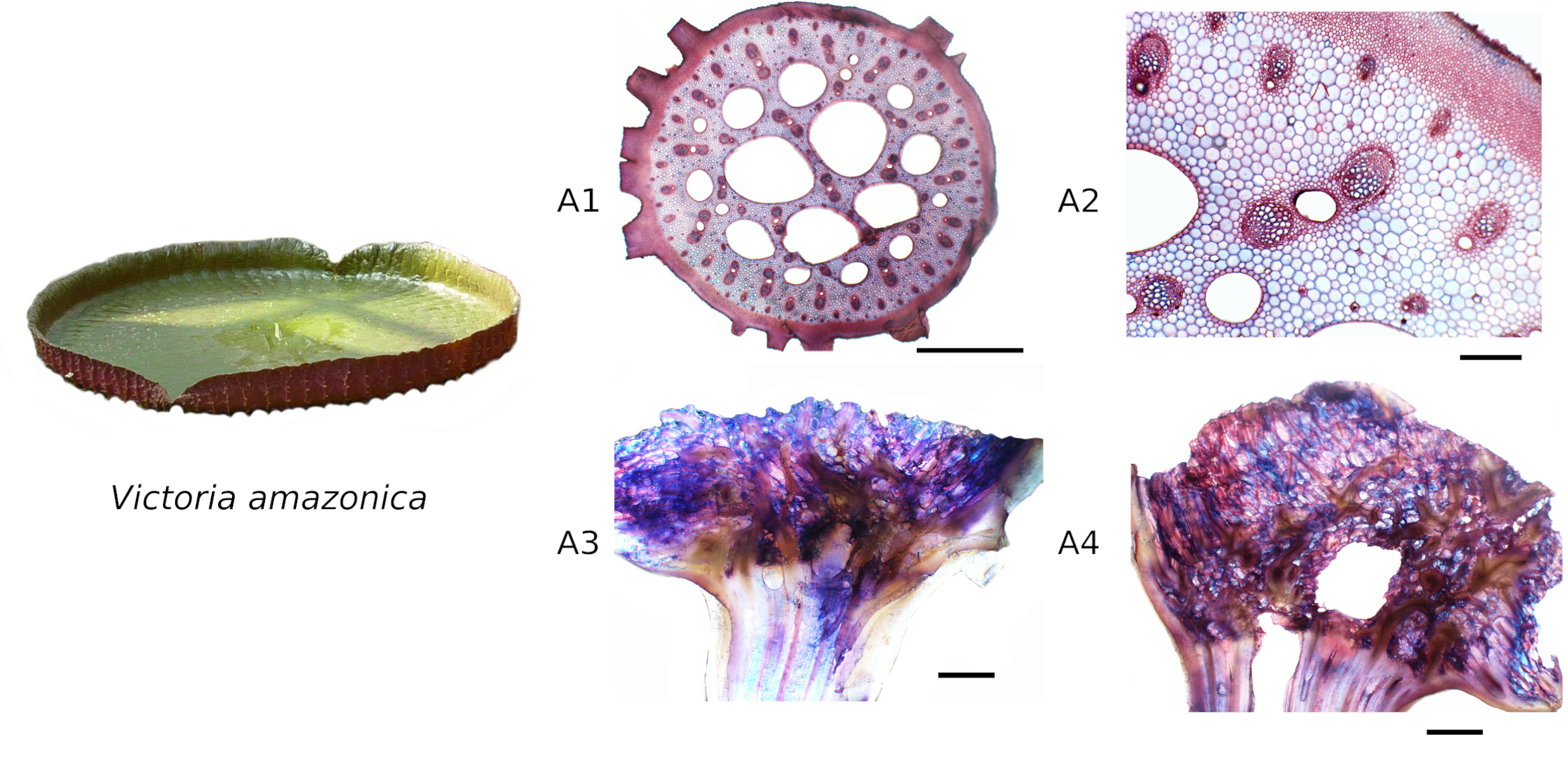

Supplement: Supplementary file 1 [file biomimetics-06-00025-s001.zip › Figure S3.tif]

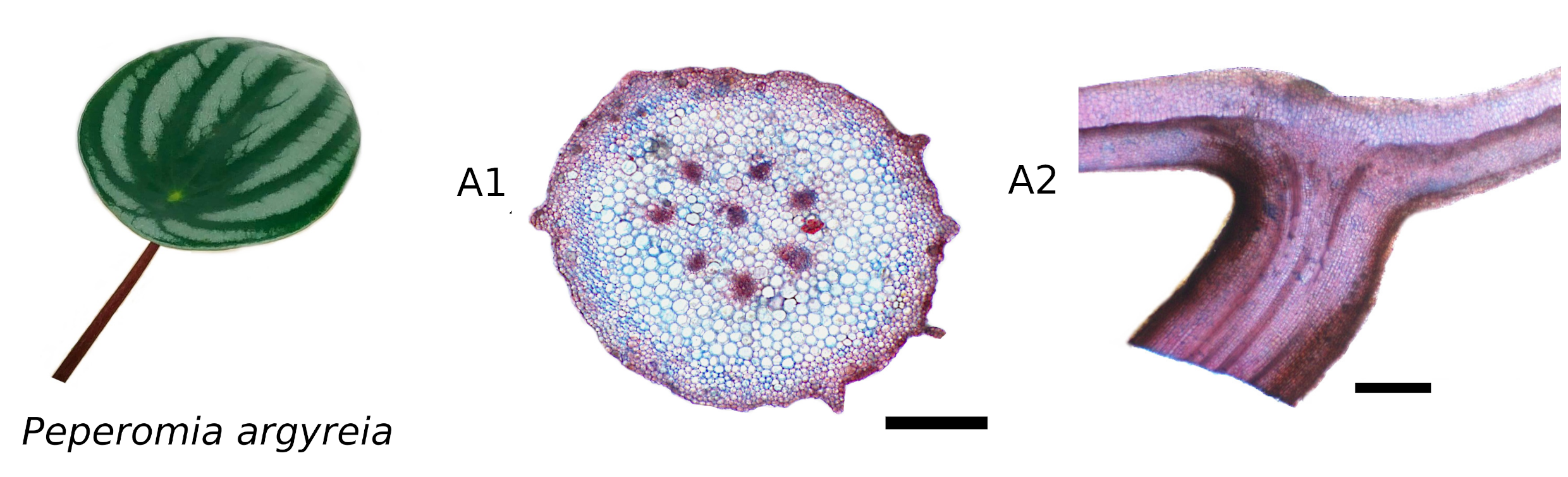

Supplement: Supplementary file 1 [file biomimetics-06-00025-s001.zip › Figure S4.tif]

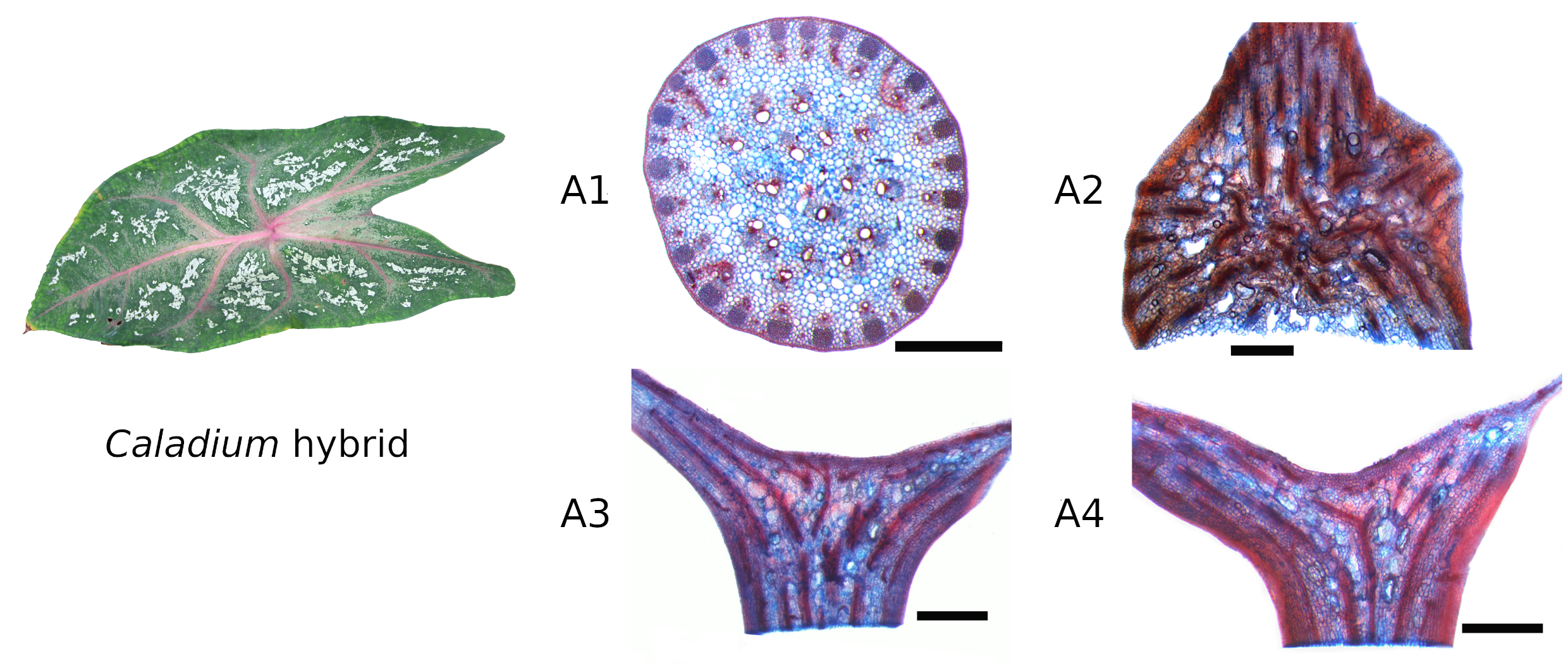

Supplement: Supplementary file 1 [file biomimetics-06-00025-s001.zip › Figure S5.tif]

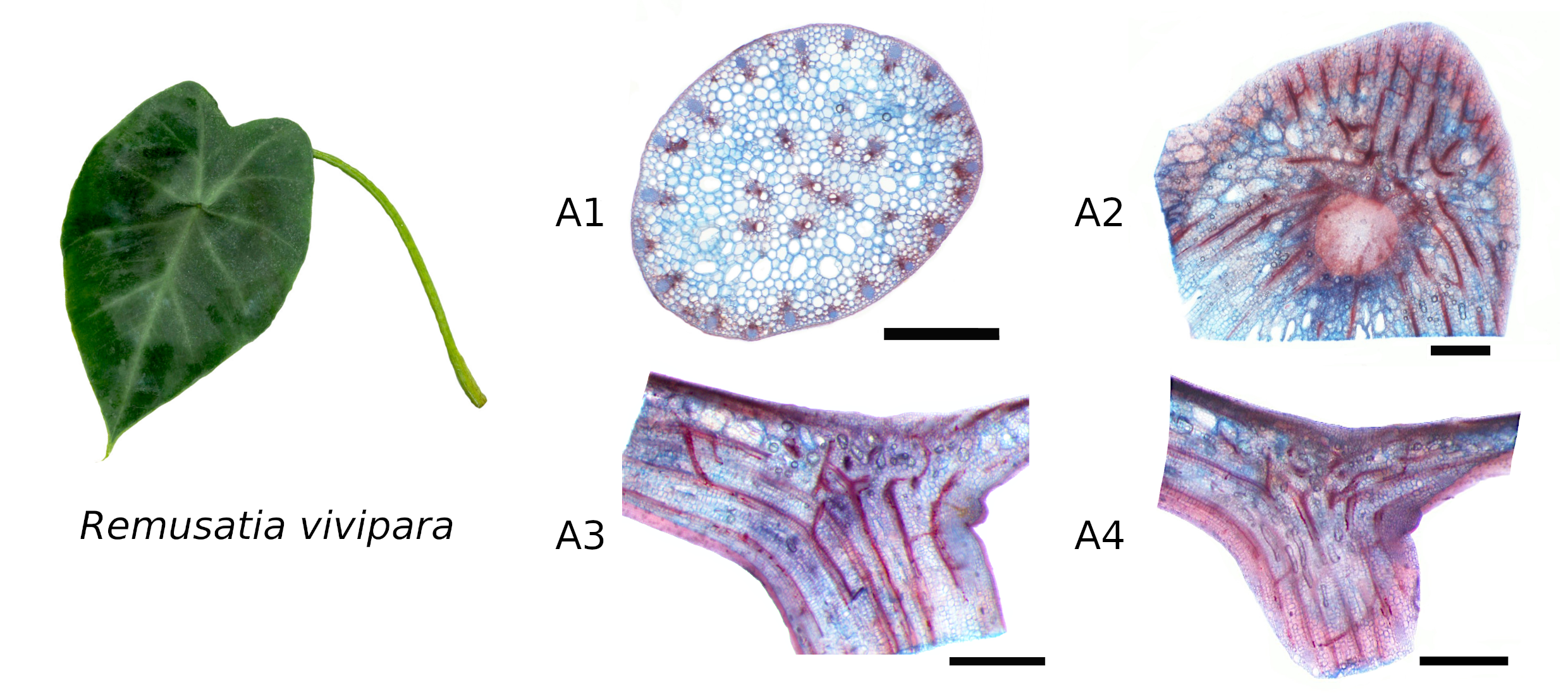

Supplement: Supplementary file 1 [file biomimetics-06-00025-s001.zip › Figure S6.tif]

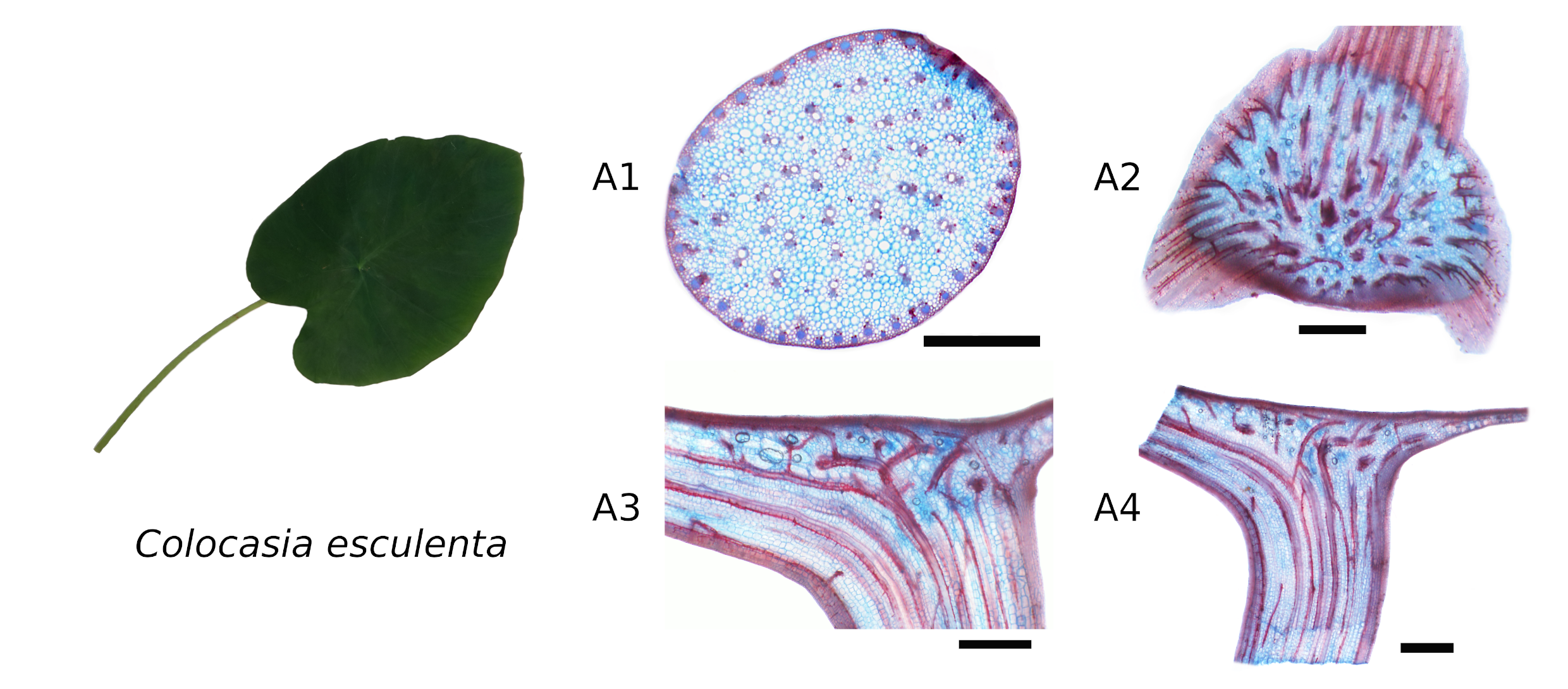

Supplement: Supplementary file 1 [file biomimetics-06-00025-s001.zip › Figure S7.tif]

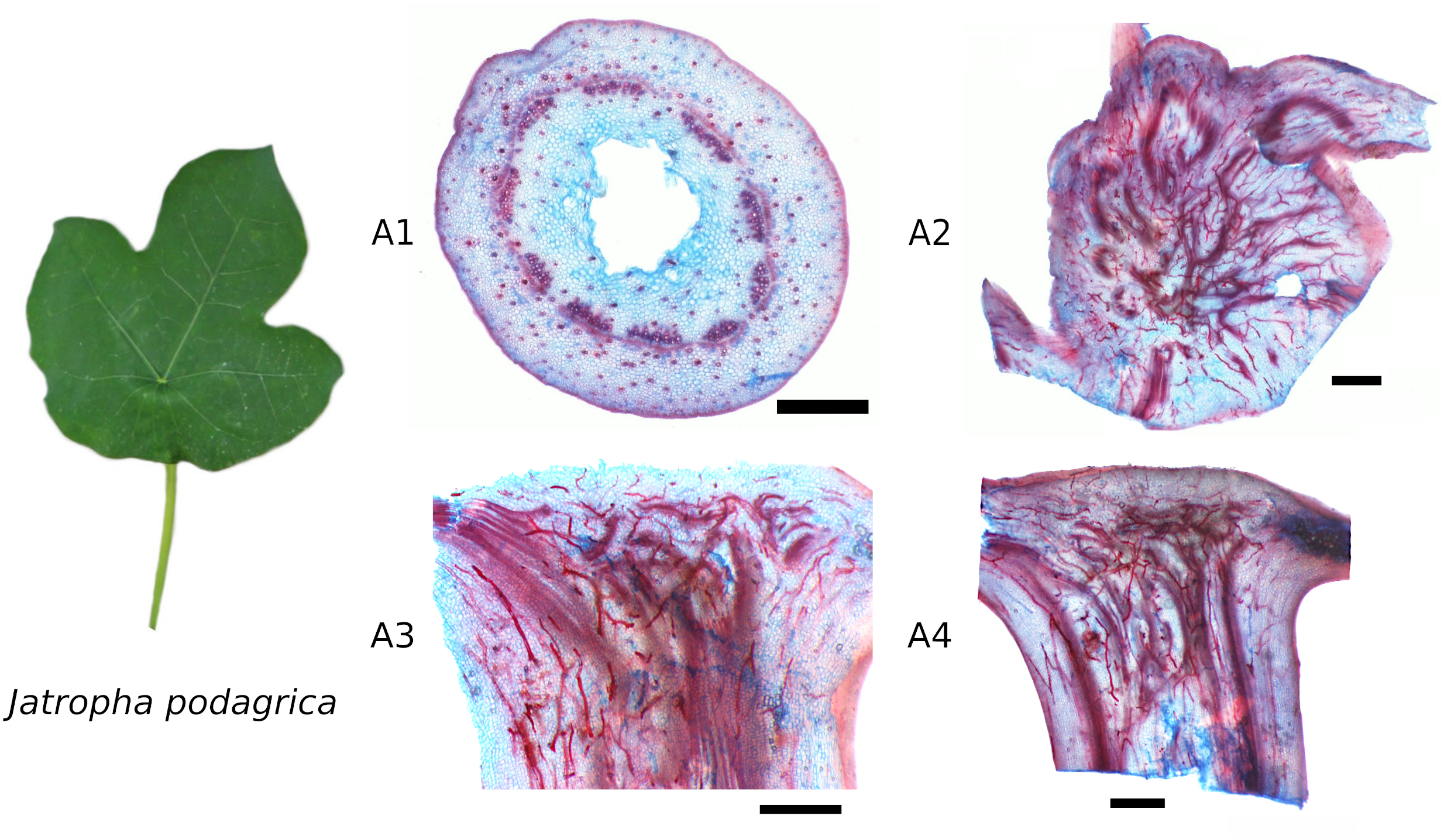

Supplement: Supplementary file 1 [file biomimetics-06-00025-s001.zip › Figure S8.tif]

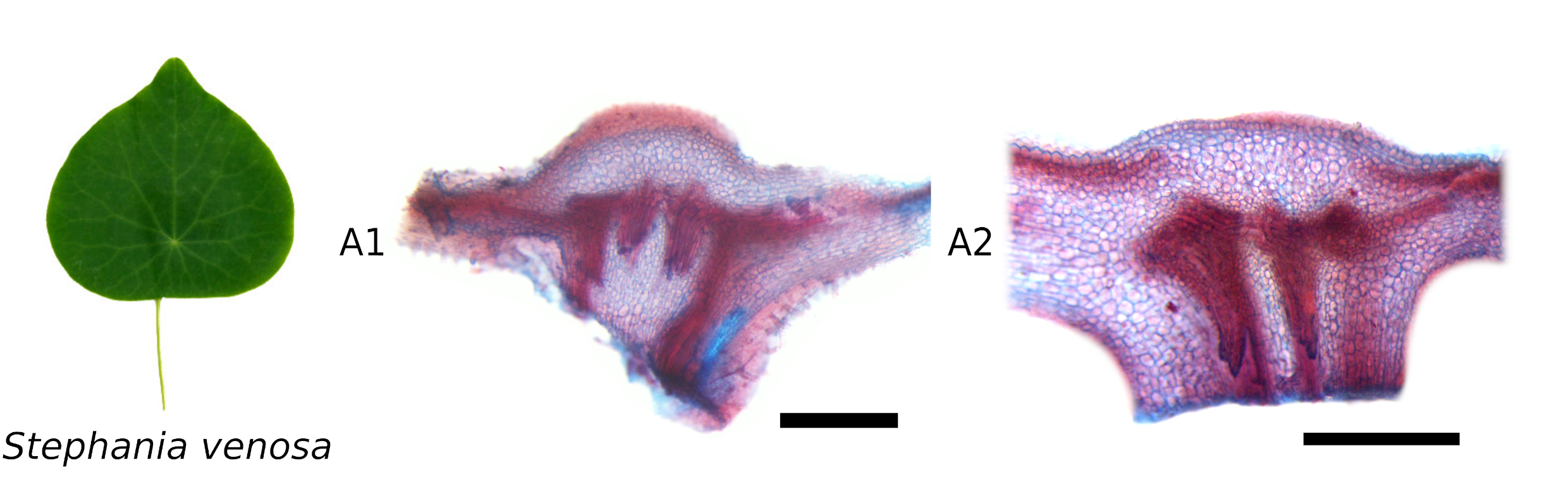

Supplement: Supplementary file 1 [file biomimetics-06-00025-s001.zip › Figure S9.tif]
